# Remodeling of pSK1 Family Plasmids and Enhanced Chlorhexidine Tolerance in a Dominant Hospital Lineage of Methicillin-Resistant *Staphylococcus aureus*

**DOI:** 10.1128/AAC.02356-18

**Published:** 2019-04-25

**Authors:** Sarah L. Baines, Slade O. Jensen, Neville Firth, Anders Gonçalves da Silva, Torsten Seemann, Glen P. Carter, Deborah A. Williamson, Benjamin P. Howden, Timothy P. Stinear

**Affiliations:** a Department of Microbiology & Immunology, The University of Melbourne at The Peter Doherty Institute for Infection & Immunity, Melbourne, Victoria, Australia; b Microbiology and Infectious Diseases, School of Medicine, Ingham Institute for Applied Medical Research, University of Western Sydney, Sydney, New South Wales, Australia; c School of Life and Environmental Sciences, University of Sydney, Sydney, New South Wales, Australia; d Microbiological Diagnostic Unit Public Health Laboratory, Department of Microbiology & Immunology, The University of Melbourne at The Peter Doherty Institute for Infection & Immunity, Melbourne, Victoria, Australia; e Doherty Applied Microbial Genomics, Department of Microbiology & Immunology, The University of Melbourne at The Peter Doherty Institute for Infection & Immunity, Melbourne, Victoria, Australia; f Infectious Diseases Department, Austin Health, Melbourne, Victoria, Australia

**Keywords:** *Staphylococcus aureus*, antibiotic resistance, chlorhexidine, genomics, plasmids

## Abstract

Staphylococcus aureus is a significant human pathogen whose evolution and adaptation have been shaped in part by mobile genetic elements (MGEs), facilitating the global spread of extensive antimicrobial resistance. However, our understanding of the evolutionary dynamics surrounding MGEs, in particular, how changes in the structure of multidrug resistance (MDR) plasmids may influence important staphylococcal phenotypes, is incomplete. Here, we undertook a population and functional genomics study of 212 methicillin-resistant S. aureus (MRSA) sequence type 239 (ST239) isolates collected over 32 years to explore the evolution of the pSK1 family of MDR plasmids, illustrating how these plasmids have coevolved with and contributed to the successful adaptation of this persistent MRSA lineage. Using complete genomes and temporal phylogenomics, we reconstructed the evolution of the pSK1 family lineage from its emergence in the late 1970s and found that multiple structural variants have arisen. Plasmid maintenance and stability were linked to IS*256*- and IS*257*-mediated chromosomal integration and disruption of the plasmid replication machinery. Overlaying genomic comparisons with phenotypic susceptibility data for gentamicin, trimethoprim, and chlorhexidine, it appeared that pSK1 has contributed to enhanced resistance in ST239 MRSA isolates through two mechanisms: (i) acquisition of plasmid-borne resistance mechanisms increasing the rates of gentamicin resistance and reduced chlorhexidine susceptibility and (ii) changes in the plasmid configuration linked with further enhancement of chlorhexidine tolerance. While the exact mechanism of enhanced tolerance remains elusive, this research has uncovered a potential evolutionary response of ST239 MRSA to biocides, one of which may contribute to the ongoing persistence and adaptation of this lineage within health care institutions.

## Abstract (precis)

Staphylococcus aureus is a significant human pathogen whose evolution and adaptation have been shaped in part by mobile genetic elements (MGEs), facilitating the global spread of extensive antimicrobial resistance. However, our understanding of the evolutionary dynamics surrounding MGEs, in particular, how changes in the structure of multidrug resistance (MDR) plasmids may influence important staphylococcal phenotypes, is incomplete.

## INTRODUCTION

Mobile genetic elements (MGEs) play a central role in microbial evolution, serving as a mechanism by which genetic material can be transferred, disseminated, and rearranged, allowing for rapid adaptation to new and changing environments. Nowhere is this more apparent than in the global dissemination of genes encoding mechanisms of antimicrobial resistance and virulence in populations of clinically significant bacteria (1–4). Staphylococcus aureus is a leading cause of bacterial infections in humans, and invasive staphylococcal disease is associated with significant morbidity and mortality (5, 6).

One of the oldest pandemic lineages of S. aureus is multilocus sequence type 239 (ST239), a multidrug-resistant, health care-associated (HA) methicillin-resistant S. aureus (MRSA) clone first identified in the late 1970s (7–9). Multiple studies have used genomics to explore the evolution of ST239 MRSA, providing insight into its global spread, extensive antimicrobial resistance repertoire, and persistence in health care environments (10–15). In Australia, ST239 has been a dominant HA-MRSA lineage for nearly 4 decades, and although its prevalence is on the decline, ST239 is still regularly identified as a cause of invasive disease in Australia, according to national surveillance reports (http://agargroup.org.au/agar-surveys/). We have previously described the long-term evolution of ST239 MRSA in eastern Australian hospitals to be one of convergent and adaptive evolution of two genetically distinct ST239 clades (termed the Australian and Asian-Australian clades) toward increased antimicrobial resistance at the cost of attenuated virulence (15). This initial work largely focused on changes occurring within conserved regions of the genome, with exploration of the accessory genome being limited.

Early ST239 MRSA isolates were first recognized in Australia because they displayed resistance to gentamicin (16–18), encoded by a bifunctional acetyltransferase-phosphotransferase gene [*aac(6′)-aph(2ʺ)*] commonly carried on pSK1-like plasmids as part of the composite transposon Tn*4001* (19, 20). pSK1 represents a family of intermediate-sized (20- to 40-kb), theta-replicating staphylococcal plasmids that have been recovered in Australia and the United Kingdom (19, 21–24). In addition to *aac(6′)-aph(2ʺ)*, other antibiotic resistance elements are variably carried by these plasmids, including *blaZ* (encoding a penicillinase), carried as part of Tn*552* (22, 23), and *dfrA* (encoding an insensitive dihydrofolate reductase conferring trimethoprim resistance), carried as part of Tn*4003*, which represents a cointegrated remnant of a pSK639-like plasmid (25, 26). Further, harbored by pSK1-like plasmids is a *qacA* gene encoding a quaternary ammonium compound (QAC) multidrug efflux pump (27), which can mediate tolerance to cationic biocides, most notably, chlorhexidine (28). An outbreak of ST239 MRSA in the United Kingdom in the early 2000s was attributed to the presence of a plasmid-borne *qacA* gene and predicted biocide tolerance (29, 30).

The divalent cationic biocide chlorhexidine digluconate (CHX) was first described in 1954 and is a fundamental component of infection control practices to prevent nosocomial infections (31, 32). It is one of the most widely used antiseptic agents because of its broad spectrum of activity against bacteria, fungi, and enveloped viruses, as well as its good safety record and general tolerability (33–35). Resistance to in-use concentrations, typically, a 0.2% to 4.0% CHX solution in water, has not been reported in S. aureus; however, the phenomenon of enhanced tolerance appears to be increasingly common (36, 37) and is reviewed elsewhere (38). CHX tolerance has been associated with the acquisition or mutation of biocide-active efflux pumps, with the most commonly reported being the QAC efflux systems (28, 39, 40). In S. aureus this is primarily *qacA* (28, 38). Most reports of staphylococcal populations evolving CHX tolerance either have demonstrated a phenotypic shift in susceptibility or have detected an increase in the prevalence of genes encoding efflux systems (38, 41). While several studies have combined phenotypic and molecular data, this work has largely been performed on clonally diverse or genomically undefined populations, and thus, the evolutionary mechanisms responsible are often unclear (36, 41).

Here we present a detailed exploration of the evolutionary dynamics surrounding the pSK1 family of plasmids, focusing on a single well-defined staphylococcal lineage, the ST239 MRSA population circulating in Australia. The primary aims of this work were to (i) describe the evolution of the pSK1 plasmid family in ST239 MRSA, with a specific focus on the emergence of plasmid structural variants, and (ii) explore the phenotypic consequences of plasmid evolution on the ST239 population, with a particular interest in the development of enhanced CHX tolerance.

## RESULTS AND DISCUSSION

**FIG 1:**
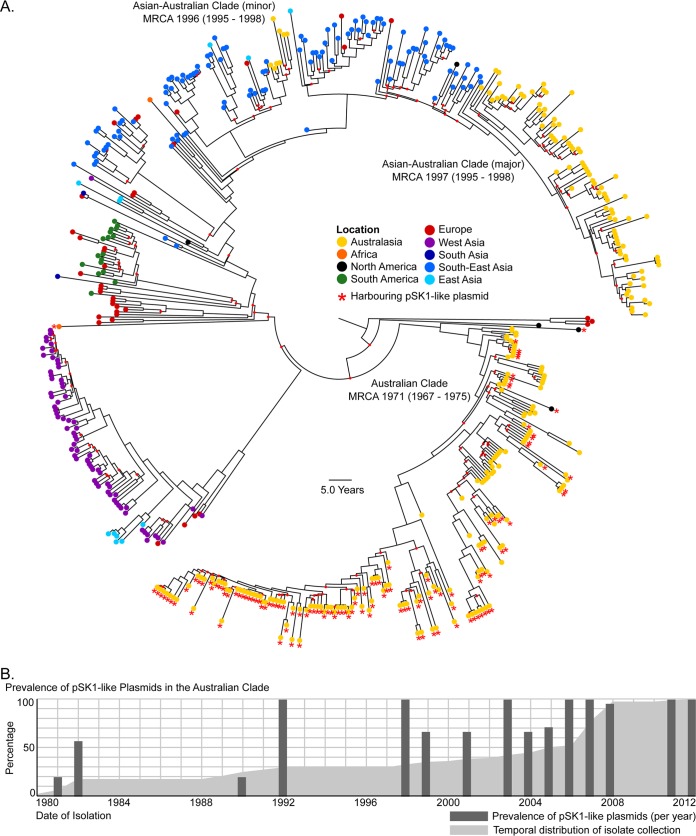
Global population structure of ST239 S. aureus isolates and prevalence of pSK1-like plasmids. (A) Illustrated is a maximum clade credibility tree inferred from the whole-genome sequence alignment of the 531 international ST239 S. aureus isolates. Tips are colored based on location (refer to the key). Internal nodes with <95% posterior support are indicated by a small red dot. The Australian and two Asian-Australian clades (major and minor) and estimates for the most recent common ancestor (MRCA) are indicated and are displayed as the median year (95% highest posterior density interval). The presence of a pSK1-like plasmid is indicated by a red star. (B) Temporal distribution of the Australian isolates included in the study (the cumulative total is indicated by the light gray region). The proportion of isolates harboring a pSK1-like plasmid (per year sampled) is indicated by the dark gray bars.

An *in silico* examination of the temporally diverse, global collection of 531 ST239 isolates looking for orthologs of the pSK1 genes revealed that pSK1-like plasmids have been maintained in the ST239 population over time but are largely harbored by a single clade, the Australian clade (Fig. 1A). The earliest isolates found to harbor pSK1 were identified in 1980, consistent with when this clone first appeared in the region (16, 17, 18). This suggested the extended coevolution of the pSK1 plasmid family and the Australian ST239 clade and subsequently indicated that this population could serve as a model in which to study the evolution of multidrug resistance (MDR) staphylococcal plasmids and the impact that extended plasmid maintenance may have on antimicrobial resistance and biocide tolerance.

### Prevalence of pSK1-like plasmids in ST239 MRSA.

Of the 124 Australian clade isolates, 92 (74.1%) carried a pSK1-like plasmid, based on the presence of plasmid gene orthologues that were largely syntenic (Fig. S1B). A comparison of plasmid carriage with the year of isolation suggested that the prevalence of pSK1-like plasmids had increased over time (Fig. 1B). While this finding may be biased by the limited number of historical isolates available, the nearly ubiquitous presence of pSK1-like plasmids in contemporary isolates (98.4% of isolates recovered after 2005) strongly suggested an evolutionary benefit for the acquisition and maintenance of these plasmids in this population.

There was no evidence of plasmid sharing between the Australian and Asian-Australian clades (see Fig. S1 in the supplemental material). However, this finding was not surprising due to the geographic separation of the two clades; the Australian clade has been predominantly recovered in the states of New South Wales and Queensland since the early 2000s, and the newer Asian-Australian clade has almost exclusively been recovered in Victoria since its introduction (15) (Fig. 1 and S1A). Further, a large proportion of the Asian-Australian clade isolates (67/88 isolates, 76.1%) harbored a different MDR plasmid, specifically, a pTW20_1-like plasmid (29) (Fig. S2). While both pSK1 and pTW20_1 share the same mode of replication (they are theta-replicating, RepA_N plasmids [43]), it is unclear whether these plasmids are incompatible, as the nucleotide sequence homology of the replication initiation A (*repA*) genes is only 74.8%.

### Structural variants of pSK1.

A closer examination of the 92 isolates in which a pSK1-like plasmid was identified showed considerable variation in the presence of plasmid gene orthologs (Fig. S1B). When aligned to a phylogenetic tree of the Australian ST239 population, it became apparent that the variation observed was phylogenetically correlated and suggested the emergence or acquisition of a limited number of pSK1-like structural variants (SVs) with subsequent clonal expansion (Fig. S1). To characterize these pSK1-like SVs, representatives for the six most common orthologue patterns underwent long-read sequencing and complete genome assembly. Additionally, the short-read *de novo* assemblies of all isolates were mined for unique plasmid features to determine the prevalence of each SV in the wider ST239 population (see the Results section in the supplemental material). Using this approach, eight distinct pSK1-like plasmids were identified, having arisen largely through the actions of IS*256* and/or IS*257*. These changes can be classified into three categories, described below.

### (i) IS-mediated gain/loss of the composite transposons Tn*4001* and Tn*4003*.

**FIG 2:**
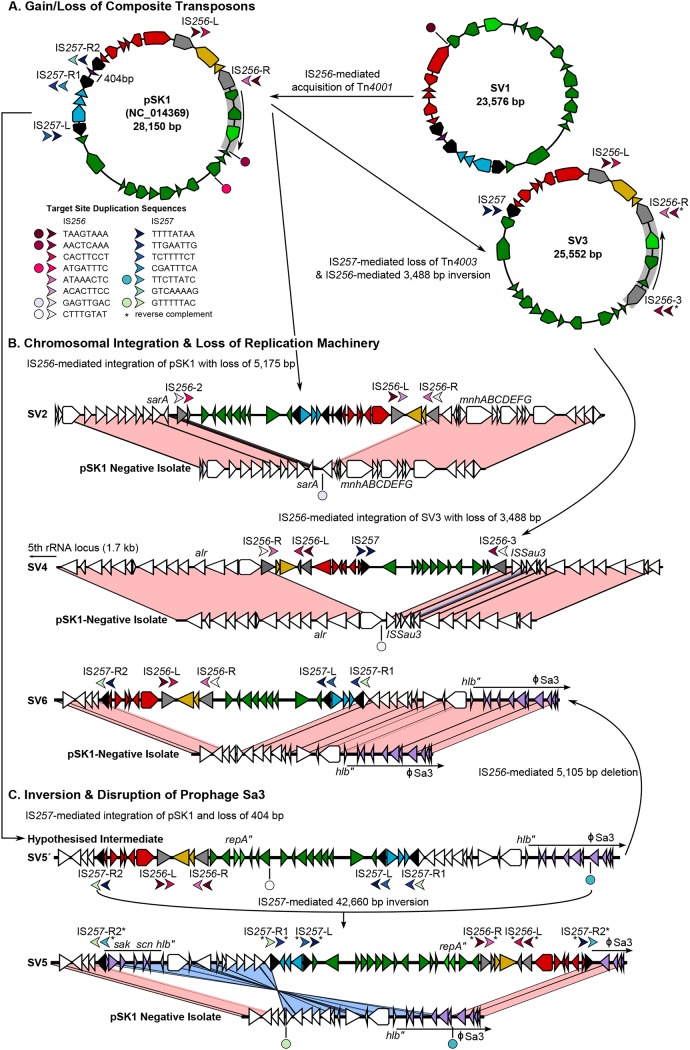
pSK1-like structural variants. Illustrated is a schematic of the structure and development of the pSK1-like plasmid variants. Plasmid genes are colored based on defined pSK1 regions (see Fig. S2A in the supplemental material), IS*256* is gray, and IS*257* is black. All chromosomal genes are white, and those encompassed within φSa3 are lilac. Arrows denote upstream/downstream target site duplications (TSD), and the direction denotes the IS orientation. Circles represent a single copy of a TSD not adjacent to an IS element. Arrows and circles are colored to reflect unique sequences (refer to the key), and an asterisk has been used to indicate a TSD present in the reverse complement to what was expected. In the structural comparisons, connected regions share ≥98% nucleotide sequence identity and are colored pink or blue to indicate the matching or reverse orientation, respectively. (A) Emergence of pSK1-like variants through IS-mediated loss/gain of the composite transposons; (B) chromosomal integration with loss/disruption of the plasmid replication machinery; (C) inversion with fragmentation of φSa3.

The structural change consisting of the insertion sequence (IS)-mediated gain/loss of the composite transposons Tn*4001* and Tn*4003* was observed in three SVs, termed SV1, SV3, and SV4 (Fig. 2). The first (SV1) was found to lack the aminoglycoside resistance-conferring Tn*4001* and was structurally equivalent to the previously identified pSK7 (Fig. 2A) (22, 44). With only a single copy of the IS*256* target site duplications (TSD) that flank Tn*4001* in pSK1 being identified, it was unclear whether SV1 had lost Tn*4001* or had never acquired it. However, as an SV1-like plasmid was recovered only from historic isolates located close to the root of a time-aware phylogenetic tree for the Australian clade (see the Results section in the supplemental material), SV1 likely represents a progenitor of pSK1 prior to gaining Tn*4001*. Both SV3 and SV4 were found to lack Tn*4003* (Fig. 2A and B). Instead, they contained only a single copy of IS*257* with the same TSD that flanked this region in pSK1 in a configuration equivalent to that in pSK14 (22, 44). Unlike SV1, the phylogenetic location of isolates carrying SV3 and SV4 strongly supports deletion of Tn*4003* (see the Results section in the supplemental material).

### (ii) IS-mediated chromosomal integration and disruption of the plasmid replication machinery.

Chromosomal integration was observed in five pSK1-like SVs. The first (SV2) emerged from IS*256*-mediated integration of pSK1 adjacent to the staphylococcal accessory regulator A (*sarA*) gene (Fig. 2B). Likewise, SV4 emerged from IS*256*-mediated integration of SV3 adjacent to a predicted aerobactin biosynthesis gene (Fig. 2B). In both cases, the formation of circular intermediates that excluded segments of the plasmid backbone is likely responsible for the loss of six and three syntenic genes in SV2 and SV4, respectively. The three remaining SVs had all arisen from a single IS*257*-mediated chromosomal integration event. Only SV5 and SV6 were represented in the complete plasmids; therefore, a hypothesized structure has been proposed for the other SV, termed SV5′ (Fig. 2B and C). In this case, chromosomal integration of pSK1 had occurred 9.2 kb upstream of a disrupted β-hemolysin gene (with the disruption resulting from integration of prophage Sa3), with the formation of a shortened circular intermediate likely being responsible for the partial deletion of Tn*4003* (Fig. 2C). SV5 (and likely SV5′) had an additional 45-bp deletion in *repA*, specifically occurring within the Rep binding boxes, which are considered essential for a functional protein (45), and SV6 had an IS*256*-mediated deletion of six syntenic genes from the plasmid backbone (Fig. 2B).

### (iii) IS-mediated inversion.

There were two inversion events identified. The first was an IS*257*-mediated inversion of a 42.7-kb region encompassing SV5′, giving rise to the structure termed SV5 (Fig. 2C). This inversion reversed the orientation of all SV5′ genes and split φSa3 (Fig. 2C). A second smaller 3.5-kb IS*256*-mediated inversion event was identified in SV3, reversing the orientation of three genes, including *repA* and the plasmid partitioning (*par*) gene (Fig. 2A). This is the same region deleted in SV4. While the exact consequences of these inversions are unknown, the division and partial inversion of φSa3 should prevent future excision of the prophage.

### Convergent evolution of pSK1-like plasmids.

**FIG 3:**
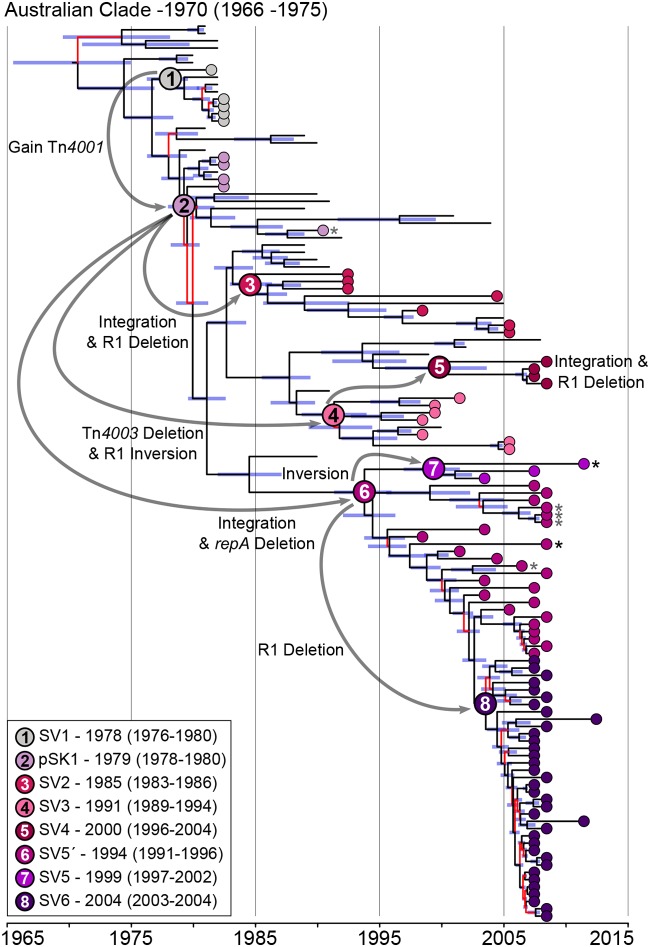
Evolutionary history of pSK1-like plasmids in the Australian ST239 clade. Illustrated is a maximum clade credibility tree inferred from the whole-genome sequence alignment of the Australian clade (*n* = 124). Branches with <95% posterior support are colored red, and the 95% highest posterior density interval for node heights is represented by the blue bars. Isolates identified as harboring a pSK1-like plasmid are indicated by a circle located at the branch tip and colored based on the SV identified (refer to the key). The most recent common ancestor for each SV is indicated by the larger node circle, with black numbers indicating an extrachromosomal SV and white numbers indicating a chromosomally integrated SV. Temporal estimates for these nodes have been provided and are displayed as SV-median year (95% highest posterior density interval). Gray arrows illustrate the likely order and type of structural change that has occurred during the evolution of the pSK1-like plasmid population. Isolates which demonstrated a plasmid gene orthologue pattern suggestive of further structural changes are indicated by an asterisk (black for deletion of the plasmid replication machinery and gray for the loss of Tn*4003*).

Analysis of unique plasmid features in the short-read *de novo* assemblies enabled assignment of all isolates harboring a pSK1-like plasmid to one of the eight plasmid SVs (see the Results section in the supplemental material). When overlaid onto a time-aware phylogenetic tree, these data demonstrated a clear pattern of stepwise change (Fig. 3); once a novel pSK1-like plasmid emerged, it was maintained and structurally remodeled during clonal expansion but rarely, if ever, horizontally transferred between ST239 isolates. Based on these data, we propose the following evolutionary history for the pSK1 plasmid family in the Australian ST239 population (illustrated in Fig. 3).

The first pSK1-like plasmids to have appeared in the Australian ST239 clade were SV1 and pSK1, which appeared in the late 1970s, consistent with the first identification of a gentamicin-resistant MRSA isolate in Australia (16–18). It was estimated that pSK1 emerged in 1979 (95% highest posterior density interval [HPDI], 1978 to 1980), following the acquisition of Tn*4001* into the ancestral SV1 plasmid. In the following 3 decades there was the emergence of SV3 in about 1991 (HPDI, 1989 to 1994), in which Tn*4003* was lost and the replication machinery was inverted. There had been at least three chromosomal integration events, resulting in the emergence of SV2 in about 1985 (HPDI, 1983 to 1986), SV5′ in about 1994 (HPDI, 1991 to 1996), and SV4 in about 2000 (HPDI, 1996 to 2004). These events likely involved pSK1 as the ancestral plasmid or SV3 as the immediate ancestor of SV4. Inversion of SV5′ in 1999 (HPDI, 1997 to 2002) led to the emergence of SV5.

In addition to this stepwise change, there is also evidence for convergent evolution in the plasmid population. The three chromosomal integration events all occurred independently. Examination of all 92 isolates harboring a pSK1-like plasmid identified a further two independent deletion events (Fig. 3). There is a clear role for chromosomal integration in improving the maintenance of plasmid genes in a population, and the subsequent deletion or disruption of the plasmid replication machinery is needed for stability of the integrant as it removes interference with chromosomal replication (46). In the Australian clade, all but two isolates that descended from an ancestral genome with an integrated pSK1-like structure have maintained it (Fig. 3). These data strongly suggest that the emergence of these phylogenetically distinct but structurally similar SVs is the result of a significant but unknown evolutionary pressure acting on the plasmid population. It is plausible that the introduction and/or increased use of antimicrobial agents and disinfectants, specifically, those for which mechanisms of resistance or tolerance are carried by these plasmids, could be contributing to the evolutionary pressure promoting plasmid maintenance and driving chromosomal integration.

### Impact of plasmid evolution on antimicrobial resistance and disinfectant tolerance.

To explore the impact of plasmid evolution on antimicrobial resistance and disinfectant tolerance, 211 isolates underwent testing for susceptibility to gentamicin, trimethoprim, and chlorhexidine. Isolates representing both the Australian and Asia-Australian clades were tested to enable comparisons between the two clades and discern which trends may be associated with the acquisition and/or evolution of the pSK1-like plasmids.

### (i) Gentamicin.

Phenotypic resistance to gentamicin was detected in 185 (87.7%) isolates. Three acquired genes encoding aminoglycoside-modifying enzymes (AMEs) were identified (47) (Table 1): (i) the bifunctional *aac(6′)-aph(2ʺ)*, found in Tn*4001* and other chromosomal and phage locations (48–50); (ii) an adenyltransferase gene [*aadD*, also known as ANT(4′)-Ia] (51, 52); and (iii) a phosphotransferase gene [*aph(3′)-IIIa*] (53), which has previously been found to be colocated with *aac(6′)-aph(2ʺ)* in an integrated φSPβ-like phage in ST239 isolates closely related to those in the Asian-Australian clade (15, 29). The bifunctional AME was common among both the Australian and the Asian-Australian clades (79.7% and 93.2%, respectively). Conversely, the distribution of the monofunctional AMEs was more distinct (Table 1). Phenotypically, the Asian-Australian clade demonstrated a significantly higher average MIC of gentamicin than the Australian clade (*P* < 0.0001; Table S1). However, as most isolates harbored multiple AMEs, phenotypic resistance could not be attributed to a single gene. With that being said, the distribution of these genes suggested that *aph(3′)-IIIa* is likely responsible for the high-level resistance observed in the Asian-Australian clade, with *aac(6′)-aph(2ʺ)* and *aadD* contributing only low-level resistance in the Australian clade (Table S1).

**TABLE 1:** Population distributions of antimicrobial and biocide resistance genes and phenotypic resistance profiles^*d*^

Isolate	Median (range) MIC (mg/liter)			Median (range) chlorhexidine MBC (mg/liter)^*b*^	Acquired resistance gene(s)^*c*^ (no. of isolates)		
	Gentamicin^*a*^	Trimethoprim^*a*^	Chlorhexidine^*b*^		AME genes	*dfr* genes	*qac* genes
All ST239 isolates (*n* = 211)	32 (0.023–256)	>32	3 (1–6)	6 (2–16)	*aac(6′)-aph2ʺ* (180), *aadD* (72), *aph(3′)-III* (95)	*dfrA* (76), *dfrG* (88)	*qacA* (156), *qacC* (2)
Asian-Australian clade (*n* = 88)	256 (0.38–256)		3 (1.5–4)	6 (3–12)	*aac(6′)-aph(2ʺ)* (82), *aph(3′)-III* (87)	*dfrG* (88)	*qacA* (67)
Australian clade (*n* = 123)	16 (0.023–256)		4 (1–6)	8 (2–16)	*aac(6′)-aph2”* (98), *aadD* (72), *aph3′-III* (8)	*dfrA* (76)	*qacA* (89), *qacC* (2)
pSK1 plasmid (*n* = 91)	24 (0.032–256)		4 (1–6)	8 (2–16)	*aac(6′)-aph(2ʺ)* (85), *aadD* (67), *aph3′-III* (5)	*dfrA* (76)	*qacA* (89)

a Phenotypic susceptibility to gentamicin and trimethoprim was determined by Etest.

b Phenotypic susceptibility to chlorhexidine was determined by broth microdilution.

c Identification of resistance genes was determined by local alignment; a minimum alignment of 70% of the gene length and >95% nucleotide sequence identity were required to call a match.

d Abbreviations: AME, aminoglycoside-modifying enzyme; MBC, minimum bactericidal concentration.

To further investigate this phenotype, linear models were developed to look for temporal trends in the data. These models identified a significant increase in the median gentamicin MIC for the ST239 population over time (*P* < 0.001). However, this trend was observed only in the Australian clade when the two clades were modeled separately (Fig. S3). This finding is similar to what has previously been observed in the ST239 population with reduced glycopeptide and daptomycin susceptibility, hypothesized to be the result of two evolutionary phenomena: (i) the introduction of the more resistant Asian-Australian clade into the region with successful local expansion and (ii) adaptive evolution within the Australian clade, collectively shifting the phenotype of the population over time (15). These same phenomena are likely contributing here. The Asian-Australian clade had already developed high-level gentamicin resistance, prior to arriving in Australia, through the acquisition of an φSPβ-like phage carrying both *aac(6′)-aph(2ʺ)* and *aph(3′)-IIIa*. Concurrently, the Australian clade had undergone adaptive evolution, with the acquisition of *aac(6′)-aph(2ʺ)* and/or *aadD* contributing to a significant increase in the MIC (Table S1) and with the uptake of a pSK1-like plasmid serving as one mechanism by which *aac(6′)-aph(2ʺ)* could have been acquired. Within the pSK1-like plasmid-harboring population, there were no discernible temporal trends (Fig. S3), which suggested that the emergence of the pSK1-like SVs has had no additional impact on the gentamicin MIC.

### (ii) Trimethoprim.

Phenotypic resistance to trimethoprim was detected in all 211 ST239 isolates, for which the MIC was >32 mg/liter. Genotypically, 75/123 isolates (61.0%) of the Australian clade and all 88 isolates of the Asian-Australian clade were found to harbor a mutated or acquired *dfr* gene (Table 1). A likely mechanism of resistance could not be identified in the remaining 48 isolates. The lack of phenotypic variation detected suggested that both the acquisition and changes in the configuration of the pSK1-like plasmids, including the deletion or alteration of Tn*4003*, had not impacted trimethoprim resistance due to an unidentified genotypic redundancy.

### (iii) Chlorhexidine.

Phenotypic tolerance to CHX, defined as an MIC of >2 mg/liter, was detected in 150 (71.1%) isolates (38). A total of 156 (73.9%) isolates were found to harbor *qacA*, and 2 were found to harbor *qacC* (Table 1). QacC is not active against CHX (38). The presence of *qacA* was significantly associated with an elevated MIC and minimum bactericidal concentration (MBC) of CHX (*P* < 0.0001) and was invariably associated with carriage of a pSK1-like plasmid (Australian clade) or a pTW20_1-like plasmid (Asian-Australian clade) (Table 2). Although *qacA* was equally prevalent in both the Australian and Asian-Australian clades (72.4% and 76.1%, respectively), the former population demonstrated a significantly higher average MIC (*P* < 0.0001, Table 2). This phenotypic disparity remained when isolates that did not carry either plasmid were excluded from the comparison. Further, no mutations in *qacAR* or variations in gene copy number that could explain this phenotypic variation were detected (see the Results section in the supplemental material), suggesting that genotypic differences occurring outside of *qacAR* were responsible.

**TABLE 2:** Investigation of chlorhexidine tolerance in Australian ST239 MRSA^*c*^

Isolate population	Group 1		Group 2		*P* values for MICs, MBCs in group 1 vs group 2
	Characteristic (no. of isolates)	Median MIC, MBC (mg/liter)	Characteristic (no. of isolates)	Median MIC, MBC (mg/liter)	
All ST239 (211)	*qacA* negative (55)	0.90, 2.30	*qacA* positive (156)	3.70, 8.00	<0.0001, <0.0001
	Australian clade (123)	3.40, 7.30	Asian-Australian clade (88)	2.30, 6.70	<0.0001, NS
	pSK1-like plasmid (91)	4.20, 8.60	pTW20_1-like plasmid (67)	3.00, 7.20	<0.0001, 0.0021
Asian-Australian clade (88)	*qacA* negative (21)	1.90, 5.20	*qacA* positive (67)	3.00, 7.20	<0.0001, <0.0001
Australian clade (123)	*qacA* negative (34)	1.80, 4.60	*qacA* positive (89)	4.30, 8.60	<0.0001, <0.0001
	Extrachromosomal SVs (17)	3.20, 7.80	Integrated SVs (74)	4.50, 8.74	0.0086, NS
	Integrated SVs + *repA* deletion (25)^*a*^	3.70, 8.50	Integrated SV + MG deletion (49)^*b*^	4.70, 8.60	NS, NS

a The population includes SV5′ and SV5.

b The population includes SV2, SV4, and SV6.

c Abbreviations: MBC, minimum bactericidal concentration; MG, multigene; SV, structural variant; NS, not significant.

**FIG 4:**
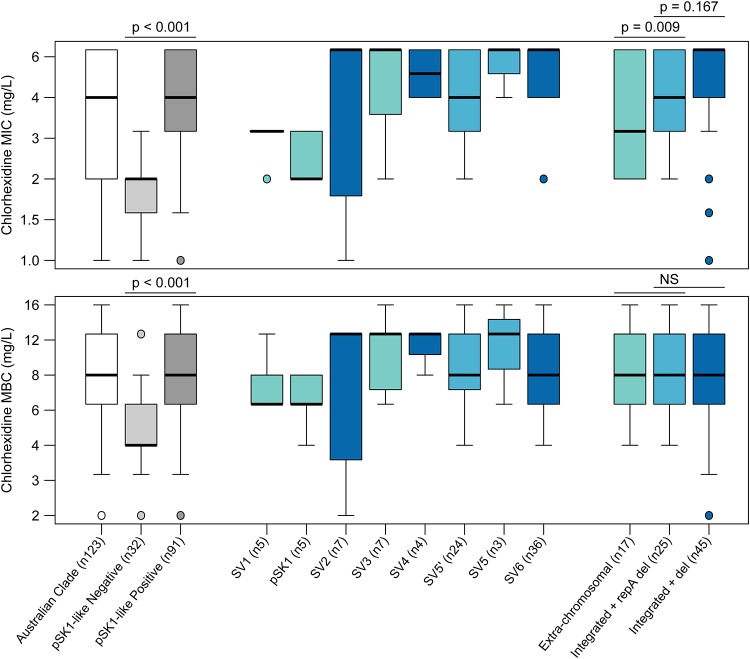
Phenotypic variation in chlorhexidine tolerance. Graphs illustrate the distribution of chlorhexidine MIC (top) and MBC (bottom) values in the Australian clade. Box plot features represent the population median (central black line), upper and lower quartiles (box), and range (bars), excluding outliers (circles). Box plots representing SVs are colored to reflect a plasmid structural feature: an extrachromosomal plasmid (teal) and a chromosomally integrated plasmid with either an internal *repA* deletion (del) (light blue) or a multigene deletion (dark blue).

As with gentamicin, linear models were developed to investigate temporal trends in CHX susceptibility (Fig. S3). These models identified a significant increase in the median CHX MIC and MBC of the ST239 population over time (*P* < 0.001). Again, this trend was observed in the Australian clade but not the Asian-Australian clade when modeled separately (Fig. S3). Further, this trend remained when only the pSK1-like plasmid-harboring population was modeled. This suggested that, in contrast to the evolutionary phenomena facilitating the development of reduced antibiotic susceptibility, adaptive evolution in the Australian clade appeared to be the primary contributor to enhanced CHX tolerance in the ST239 population. Comparison of the average MIC and MBC between the pSK1-like plasmids suggested that the more recently emerged SVs (SV2 to SV6) may be associated with enhanced tolerance (Fig. 4; Table S2). When isolates were grouped based on their carriage of structurally similar SVs, chromosomal integration was significantly associated with an increased CHX MIC (*P* = 0.0086; Fig. 4; Table 2). Furthermore, a multigene deletion in the plasmid backbone appeared to be associated with a further increase in the MIC, although this was statistically nonsignificant (Fig. 4; Table 2). It is unclear why these structural changes were associated only with a shift in the MIC, with all groups having demonstrated highly consistent average MBC values (Fig. 4; Table S2), but this finding may reflect the specific mechanisms mediating enhanced CHX tolerance or the method utilized to measure susceptibility.

### Independent association of CHX tolerance with pSK1-like plasmid evolution.

**FIG 5:**
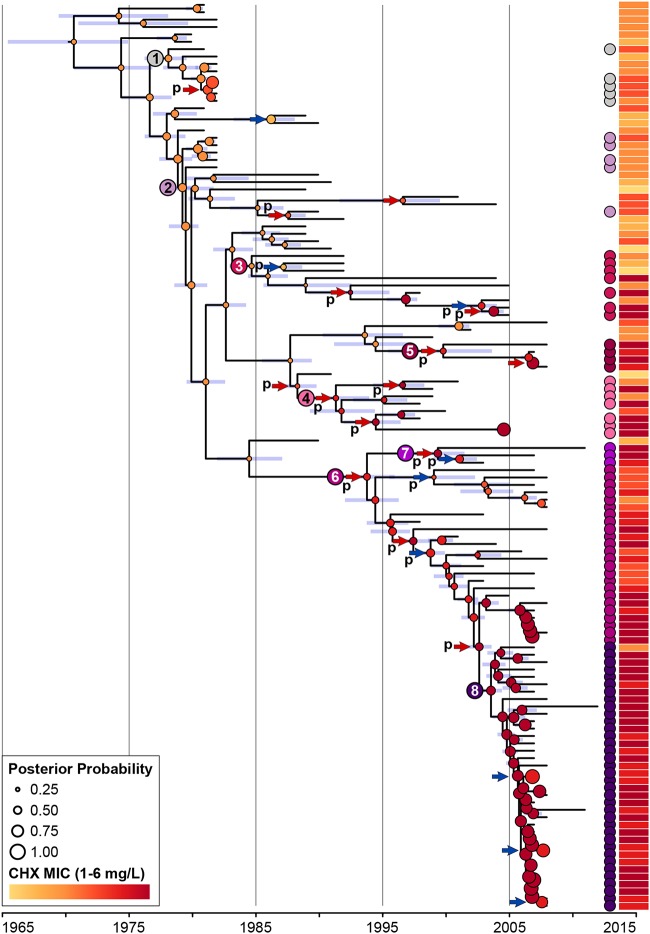
Bayesian phylogenetic model associating chlorhexidine tolerance with pSK1-like plasmid evolution. Illustrated is a maximum clade credibility tree inferred from the whole-genome sequence alignment of the Australian clade (*n* = 124). Isolates identified as harboring a pSK1-like plasmid are indicated by a circle located adjacent to the tree and colored based on the SV identified. Blue bars represent the 95% highest posterior density interval for the node heights. The ancestral nodes in which each SV is estimated to have emerged are indicated by a number (the numbers are the same as the numbers in Fig. 3). The estimated CHX MIC for all ancestral nodes is indicated by a circle, colored based on the MIC value and sized according to the posterior probability for the estimate (refer to the key). The 24 nodes with a predicted shift in the MIC are indicated by an arrow, colored blue or red for a decrease or increase in the MIC compared to that in the preceding node, respectively. Those that have been tentatively associated with the changes in plasmid configuration are labeled with “p.” The aligned heat map illustrates the phenotypic MIC values attained for each isolate.

To examine the strength of the association between plasmid evolution and the development of enhanced CHX tolerance, in addition to exploring other possible mechanisms that may be contributing to this phenotype, three statistical genomic techniques were utilized. A series of genome-wide association studies (GWAS) and discriminant analysis of principal components (DAPC) studies was performed, but none identified any significantly associated genotypes, outside of the genotype resulting from the presence of *qacA*-harboring plasmids, that could be responsible for enhanced CHX tolerance (see the Results section in the supplemental material). We therefore performed a modified Bayesian phylogeographic analysis, modeling the CHX phenotype rather than the geographic location of the ancestral genomes. The hypothesis was that if the development of CHX tolerance was a consequence of plasmid evolution, then the ancestral nodes (ANs) from which the SVs were estimated to have emerged should correlate with those in which a shift in MIC is predicted (Fig. 5).

In this model, a shift in MIC was predicted at the expected ANs or at the directly preceding node for the emergence of the newer SVs (SV3, SV4, SV5′, SV5, and SV6; Fig. 5). In the older SVs, a shift in MIC was modeled to have occurred a few nodes following the emergence of the SV (SV1, pSK1, and SV2; Fig. 5). This discrepancy is likely due to the higher proportion of isolates not harboring a pSK1-like plasmid in close proximity to these ANs, with a shift in MIC being predicted at the ANs for subclades of isolates harboring these older SVs (Fig. 5). Overall, this model did suggest that plasmid evolution was associated with shifts in CHX tolerance, but it is unlikely the sole contributing mechanism. Of the 24 nodes (19.5% of all nodes) in which a shift in MIC was detected, only 18 appeared to be associated with a change in plasmid configuration. Further lab-based experimentation is required to confirm these findings.

Chlorhexidine is one of the most commonly used biocides in the health care environment and a fundamental component of infection control initiatives shown to be effective in reducing the rates of invasive staphylococcal disease (54, 55). Therefore, it is plausible that its widespread use may be contributing to the evolutionary pressures promoting maintenance of the pSK1-like plasmid family in ST239 MRSA. However, the *qacA*-encoded efflux pump is active against a range of organic cations (28, 38), and this detected increase in tolerance may extend to other biocides, making them potential drivers of plasmid evolution. In a separate finding, all pSK1-like plasmids maintained an ∼9.3-kb segment of the plasmid backbone, demonstrating >95% nucleotide sequence identity to S. warneri plasmid pPI-1 (GenBank accession number AB125341.3) (44, 56). The genes encompassed within this region encoded protein products predicted to be cell envelope associated and involved in membrane transport and, potentially, iron acquisition, with one gene encoding a putative Fst-like toxin as part of a type I toxin-antitoxin system (44). The ubiquitous conservation of this region in the pSK1-like plasmid population suggested a potential benefit of these encoded products to the ST239 population and the possibility that they also contribute to the evolution and maintenance of this plasmid population (44).

### Conclusions.

Plasmids and other MGEs play a central role in the successful evolution and adaptation of bacterial populations but are often overlooked because of the challenges with examining some plasmid DNA sequences using short-read data. Here, we have provided a comprehensive analysis of the evolution of the pSK1-like plasmid population that has coevolved with the Australian ST239 MRSA lineage over multiple decades. Within this ST239 population, there are at least eight structurally distinct pSK1-like plasmids, having arisen largely through the actions of IS*256* and IS*257*. Further, this plasmid population has undergone convergent evolution, with the repeated emergence of chromosomally integrated SVs. In investigating how plasmid evolution may alter phenotypic susceptibility to antibiotics and biocides, it was identified that plasmid acquisition is associated with a significant increase in gentamicin resistance and CHX tolerance. Further, carriage of a chromosomally integrated plasmid variant was associated with a further increase in CHX tolerance. While the mechanism mediating tolerance remains unclear, we speculate that it is linked to altered regulation of the *qacAR* efflux system due to movement of the IS elements. Collectively, these findings support the idea that the widespread and increasing use of cationic biocides may potentially be contributing to the ongoing evolution of the pSK1-like plasmid population. Although the levels of CHX at which isolates show reduced susceptibility observed in this study remain well below the in-use concentrations of CHX, they do illustrate an evolutionary response in ST239 MRSA, one that may provide an adaptive advantage for this lineage in health care institutions.

## MATERIALS AND METHODS

### Bacterial isolates.

This study utilized a temporal (1980 and 2012) and geographically diverse collection of 212 Australian ST239 S. aureus isolates. Two isolates (including reference strain JKD6008) were recovered in New Zealand (57). All isolates represented cases of clinical infection. To establish the global phylogenetic context for the collection, we supplemented these data with the WGS data for a further 319 international ST239 S. aureus isolates. Further information about the selection of isolates and relevant demographic information can be found in the Materials section in the supplemental material.

### WGS and sequence data.

The whole-genome sequencing (WGS) data for 368/531 isolates (73/212 Australian isolates) have been previously published. Seven isolates were subjected to long-read sequencing to enable complete genome assembly. Information about the generation of novel sequence data and relevant WGS information can be found in the Materials section in the supplemental material.

### Bioinformatic analysis.

The bioinformatic analyses performed for this study have been explained in detail in the Materials section in the supplemental material and are briefly outlined here. Short-read sequence data were mapped to reference strain S. aureus JKD6008 (GenBank accession number CP002120 [58]) using Snippy (v3.2) (https://github.com/tseemann/snippy). Maximum likelihood phylogenetic trees were generated with IQ-TREE (v1.6.1) (59), and maximum clade credibility trees were generated with BEAST (v2.4.7) (60). Trees were visualized in FigTree (v1.4.3) (http://tree.bio.ed.ac.uk/software/figtree/), and figures were assembled in Inkscape (v0.91) (https://inkscape.org/). Short-read sequence data were *de novo* assembled using SPAdes (v3.11.0) (61) and annotated with Prokka (v1.12) (62). Long-read sequence data were *de novo* assembled using the SMRT analysis system (v2.3.0.140936) (Pacific Biosciences), circularized and reoriented in Geneious (v8.1.5) (Biomatters), and polished with Snippy (v3.2). Plasmid structural comparisons were conducted using the Artemis Comparison Tool (63). Ortholog clustering was performed using Roary (64).

### Antimicrobial susceptibility testing.

Testing for phenotypic susceptibility to trimethoprim and gentamicin was performed using Etests (bioMérieux), and the results were interpreted using CLSI guidelines (65). Susceptibility to CHX was performed using a modified broth microdilution method (see the Materials section in the supplemental material). All isolates were tested in biological triplicate, and the median values were used for statistical analysis, performed in R (v3.4.2) software (http://www.R-project.org/).

### Accession number(s).

All sequence data and complete genomes novel to this study have been submitted to the European Nucleotide Archive under project accession number PRJEB29322.

## Supplementary Material

Supplemental file 1
