# Supplementary material for: Remodeling of pSK1 Family Plasmids and Enhanced Chlorhexidine Tolerance in a Dominant Hospital Lineage of Methicillin-Resistant Staphylococcus aureus
Source: Antimicrob Agents Chemother. 2019 Apr 25;63(5):e02356-18. doi: 10.1128/AAC.02356-18 (PMC6496109; doi:10.1128/AAC.02356-18)
Supplement: Supplemental file 1 [file AAC.02356-18-s0001.pdf]

Supplementary Materials

1. **Supplementary Methods.** This file contains additional information about materials and methods used in this study.
2. **Supplementary Results.** This file contains additional analyses and results relevant to this study. Part A: Description of the pSK1-like plasmid variants; Part B: Investigation of the association between plasmid evolution and biocide tolerance.
3. **Supplementary Dataset.** This file summarises the relevant isolates demographics and WGS information including sequence data accession numbers for all isolates included in this study.
4. **Supplementary Tables.** This file contains additional tables which provide information about the distribution of gentamicin and chlorhexidine susceptibility in the ST239 MRSA population.
5. **Supplementary Figure S1. pSK1 plasmid gene presence and synteny.** (A) Maximum likelihood phylogenetic tree inferred from 3,883 core genome SNPs illustrates the population structure of ST239 *S. aureus* in Australia. Tips are coloured based on location (refer to key). Branches with < 70% bootstrap support are coloured red. (B) Coloured blocks represent the identification of a pSK1 gene orthologue, using a 95% amino acid identity threshold (excluding insertion sequences). Box length is reflective of gene length and ordered based on pSK1 (Figure S2A). Boxes are linked if orthologs were found to be syntenic. Coloured boxes reflect the six identified patterns.
6. **Supplementary Figure S2. Structure of pSK1, pTW20_1 and pBPH2003 plasmids.** Plasmid genes have been coloured based on defined plasmid regions. Insertion sequences (IS) are coloured grey (IS*256*) and black (IS*257*), with target site duplications (TSD) illustrated: arrows indicate upstream/downstream sequences, orientation, and are coloured to represent unique sequences (refer to key). (A) pSK1; sequence and annotations are that previously published, accession NC_014369 (1). (B) pTW20_1; sequence and annotations are that previously published, accession NC_017352 (2). (C) pBPH2003; a pTW20_1-like plasmid representative of that harboured in isolates from the Asian-Australian ST239 clade.
7. **Supplementary Figure S3. Modelling temporal-association in phenotypic susceptibility data.** Graphs depict linear models developed to explore the potential association between gentamicin MIC and chlorhexidine MIC and MBC with the year in which isolates were recovered. The dotted line indicates the smoothed mean MIC and the bold line indicates the fitted linear model. Four populations were tested (from top to bottom): (i) All ST239 MRSA (n = 211), (ii) the Asian-Australian clade (n = 88), (iii) the Australian clade (n = 123), and (iv) the pSK1-like plasmid harbouring population (n = 91). Note: reference JKD6008 (Australian clade) was not tested.
8. **Supplementary Figure S4. Exploration of gene presence/absence signatures associated with phenotypic chlorhexidine tolerance.** The graphs illustrate the findings from two Discriminant Analysis of Principle Components (DAPC) models, used to investigate genetic signatures in the ST239 MRSA population associated with chlorhexidine MIC. The first model (top panel) examined all accessory gene orthologues (clustered at 95% amino acid homology), and in the second model (bottom

panel) gene orthologues associated with either pSK1 or pBPH2003 (representative of the pTW20_1-like plasmid recovered from the Asian-Australian clade) were excluded. The prior hypothesised sub-divisions represent the phenotypic CHX MIC values for the population, as indicated in the key. (A) Scatter plots illustrate the clustering of isolates across the two most discriminant functions. The density plots illustrate the same data in a one-dimensional format for the first (B) and second (C) most discriminant functions. (D) Loading plots illustrate the contributions of all gene orthologues to the DAPC models, with the most highly contributing variables (> 0.01) coloured based on whether they represent a plasmid-associated (green) or chromosomal gene orthologue (blue). (E) Membership graphs illustrate the membership profiles for all isolates, aligned to a maximum likelihood phylogenetic tree for the ST239 population. The probability index indicates the likelihood of assigning an isolate back to one of the hypothesised sub-divisions.

9. **Supplementary Figure S5. Exploration of mutations associated with phenotypic chlorhexidine tolerance.** (A) Manhattan plot illustrates the results of three Genome Wide Association Studies (GWAS) conducted to identify core genome SNPs associated with CHX MIC, tested at three thresholds: MIC ≥ 3 mg/L, ≥ 4 mg/L or = 6 mg/L. Red line indicates the threshold for significance (with Bonferroni correction). (B) Maximum likelihood phylogenetic tree is that from Figure S1, and adjacent are the phenotypic CHX MIC and MBC values (coloured yellow to red with increasing value). (C) The presence (blue) or absence (white) of the 50 most significantly associated mutations detected at each phenotypic threshold are illustrated (refer to key).

Supplementary Methods

Bacterial Isolates. This study utilised a collection of 212 Australian ST239 *S. aureus*, selected from available collections for temporal and geographic diversity. Isolates were recovered between 1980 and 2012 from the Australian states of Victoria (n = 123), New South Wales (n = 62), Queensland (n = 22), South Australia (n = 2), and Western Australia (n = 1). Two isolates, including reference *S. aureus* JKD6008, were recovered in New Zealand (3). Bacterial isolates and corresponding whole genome sequence (WGS) data were sourced from:

- The complete genome of JKD6008 was sourced from (4).
- 73 isolates were sourced from (5). Six isolates from the original study were excluded because they represented secondary samples from patients already represented in the collection. These isolates have only been included in the generation of the global phylogenetic model (described below).
- 104 isolates were sourced from the collection of the Australian and New Zealand Cooperative on Outcome in Staphylococcal Sepsis (ANZCOSS) study (6). All ST239 from unique patients selected for the ANZCOSS sub-study were included (7).
- 16 isolates were sourced from the collection of the Vancomycin Efficacy in Staphylococcal Sepsis in Australasia (VANESSA) study (8). All ST239 isolates from unique patients were included.
- 18 isolates were sourced from the historic collections (1989-1994) housed at the Microbiological Diagnostic Unit Public Health Laboratory, Melbourne, Australia. All ST239 isolates from unique patients in which a viable bacterial isolate could be recovered were included.

To generate a global phylogenetic model for the ST239 lineage the Australian ST239 collection was supplemented with the publicly available WGS data for 319 globally diverse ST239 isolates, selected to maximise temporal, geographic and genetic diversity of the collection. The WGS data for these isolates was sourced from:

- The complete genomes of eight ST239 isolates were sourced from (2, 9-15).
- 174 isolates were sourced from the collections of (16-22). All ST239 isolates that could be accessed from these studies were included.
- 78 isolates were sourced from the collections of (23, 24). A limited number of isolates were included from both studies, selected to maximise the temporal and geographic diversity of these collections
- 29 isolates were sourced from the 2015 Australian Staphylococcal Sepsis Outcome Program (ASSOP). Sequence data for all ST239 isolates was kindly provided by the Australian Group on Antimicrobial Resistance (http://www.agargroup.org/).
- A further 24 isolates were sourced from historic collections (1989 – 1994) housed at the Microbiological Diagnostic Unit Public Health Laboratory, Melbourne, Australia.

Whole Genome Sequencing & Sequence Data. The WGS data for 42 isolates is novel to this study (Supplementary Dataset). Genomic DNA was extracted with the JANUS Automated

Workstation, using the Chemagic DNA/RNA kit (Perkin Elmer). Unique dual index libraries were prepared using the Nextera XT DNA preparation kit (Illumina). Libraries were sequenced on a NextSeq platform (Illumina) with 2 x 150 bp chemistry. Long read sequencing was conducted on seven isolates (BPH2003 [pTW20_1-like], BPH2019 [SV3], BPH2056 [SV1], BPH2070 [SV2], BPH2869 [SV6], BPH3244 [SV4], and JKD6009 [SV5]). Genomic DNA was extracted using the DNeasy Blood & Tissue extraction kit (Qiagen) and sequencing was conducted on the Pacific Biosciences RS-II platform with P6-C4 chemistry. WGS data accession numbers can be found in the Supplementary Dataset.

Genome Assembly. Long-read sequence data was *de novo* assembled in the SMRT Analysis System v2.3.0.140936 (Pacific Biosciences), using the protocol previously described (25). Briefly, raw sequence data was first assembled using the HGAP v3 protocol, with a minimum seed read length of 10,000 bp, genome size of 3 Mb, target coverage of 10 and an overlapper error rate of 0.04. Contigs underwent a second round of error correction using Quiver v1, then were circularised with the overlap removed and orientated to *dnaA* or *repA* in Geneious v8.1.5 (Biomatters). Long reads were aligned to the assembly using BridgeMapper v1 and manually checked for assembly errors. The consensus sequence from this alignment was further error corrected with high quality short-read Illumina data using Snippy v3.2 (https://github.com/tseemann/snippy/), performed in an iterative process until no variants were detected. The final sequences were annotated with Prokka v1.12. Structural comparison of the complete plasmid sequences were conducted using the Artemis Comparison Tool (26), filtering out hits < 100 bp in length.

Short read sequence data was *de novo* assembled using SPAdes v3.11.0 (27), excluding the pre-assembly error correction ("--only-assembler"), including post-assembly error correction ("--careful") and a minimum coverage threshold of five ("--cov-cutoff") to remove potential contaminants. Assemblies were annotated using Prokka v1.12 (28) and contigs < 200 bp in length were excluded. Contig coverage information was extracted from the assembly data, with the ratio of coverage between chromosome and plasmid associated contigs used to approximate plasmid and gene copy number.

Orthologue Clustering & Gene Synteny. The presence/absence of plasmid gene orthologues was used to screen short-read assemblies for the presence of a pSK1-like plasmids. The pangenome pipeline Roary (29) was used to perform the ortholog clustering. The protein coding sequences (CDS) identified in the annotated complete genomes and short-read *de novo* assemblies where clustered into orthologous groups at different thresholds of amino acid homology (75%, 80%, 85%, 90%, and 95%), without splitting paralogs (option "-s"). No significant variation was observed in the clustering across the different homology thresholds (data not shown), subsequently all analyses published here use a 95% amino acid homology threshold. Genes were deemed to be syntenic if the CDS were identified as adjacent on a single contig.

Phylogenetic Analysis. The Illumina short-read sequence data for the 211 Australian ST239 *S. aureus* were mapped to the complete genome of reference JKD6008 (CP002120, (4)), using Snippy v3.2 (https://github.com/tseemann/snippy/). An alignment of core genome single

nucleotide polymorphisms (SNP) was generated using a minimum coverage of 10 reads, and base-call stringency of 90%. SNPs were deemed core if a base was called for every isolate in the alignment at that site. This alignment was used as the input to generate a maximum likelihood phylogenetic tree with IQ-TREE v1.6.1 (30), modelled with a General Time Reversible (GTR) nucleotide substitution model, empirical base frequencies, a correction for the absence of invariant sites, use of a discrete Gamma model with four rate categories (model option "GTR+F+I+G4"), and 1,000 ultrafast bootstrap replicates (option "-bb 1000"). Two additional mapped alignments were generated: (i) to represent the global ST239 collection (n = 531, mapped to JKD6008), and (ii) to represent the Australian ST239 clade (n = 124, mapped to JKD6008) using the process described above. Recombination was assessed using ClonalFrameML v.1.11 (31). However, as very few recombinant events were detected (data not shown) masking these sites prior to temporal phylogenetic analysis was deemed unnecessary. The package BEAST v2.4.7 (32) was to construct the time-aware phylogenetic models. From the whole genome alignments, the core genome SNPs were extracted, grouped into a single partition and modelled with the following parameters. The year of isolation was provided as a prior. The GTR model of nucleotide substitution was selected, with the substitution rate estimated using four gamma categories. A relaxed lognormal clock model and a coalescent constant population model were selected (based on previous ST239 studies (5, 33)). Invariant sites were included as a constant pattern, weighted based on the nucleotide proportions of reference JKD6008. For the Australian clade, chlorhexidine MIC data was modelled as a discrete trait. Five 50 million step iterations were run and examined for convergence in Tracer v1.6 (http://tree.bio.ed.ac.uk/software/tracer/). The burn-in was adjusted for each run to a point at which a stable likelihood was achieved. All runs converged, and none were discarded, based on the comparisons of Bayes Factors, calculated from the harmonic means of the run likelihoods, and the attainment of effective sample sizes (> 200) in all measurements. Runs were joined and re-sampled at a frequency that would achieve ~10,000 trees using LogCombiner. Maximum clade credibility (MCC) trees with median node heights were generated in TreeAnnotator.

Genome Wide Association Study (GWAS). Allelic association was performed using the GWAS tool Plink (34). Core genomes SNPs (identified as described above) were used as the input. Three phenotypic divisions were tested, with the tolerant populations defined as a MIC ≥ 3, ≥ 4 or 6 mg/L. A Bonferroni correction was used to account for multiple hypothesis testing, the significance threshold determined as 0.05/number of SNPs tested.

Discriminant Analysis of Principal Components (DAPC). DAPC was implemented in R v3.4.2 (http://www.R-project.org/) using the package adegenet (35). Presence or absence of the CDS ortholog groups (identified as described above) were used as the input for the DAPC models, filtering out core ortholog groups (present in ≥ 210 isolates) and singletons groups (present in ≤ 3 isolates). Two models were generated: (i) all CDS ortholog groups that met the input criteria, and (ii) the same data set excluding all plasmid-associated CDS. In both models, isolates were assigned to prior populations based on chlorhexidine MIC values (1, 1.5, 2, 3, 4, and 6 mg/L). DAPC models were assessed individually to determine the optimum number of

principle components to be retained, based on the a-score and cross validation functions in adegenet.

Antimicrobial Susceptibility Testing. Phenotypic susceptibility testing for gentamicin and trimethoprim was performed using E-tests (bioMérieux), following the manufacturer's recommendations. *S. aureus* isolate ATCC29213 was used as a control. All results were read in triplicate and interpreted using CLSI guidelines (M100S, 26th Ed).
Phenotypic susceptibility testing to chlorhexidine (CHX) was performed by broth microdilution, as per the CLSI protocol for antimicrobial susceptibility testing (M07A10E, January 2015). Chlorhexidine digluconate was sourced from Sigma-Aldrich (#C9394) and diluted in sterile H_2O to 128 mg/L. In microtiter trays (Costar, #3799), serial dilutions of 100µl of CHX in cation adjusted Muller Hinton (CaMH) broth (Oxoid) were generated to a final concentration range of 1.0 - 32 mg/L, tested in 1.5x increments. A second concentration range of 0.25 - 8 mg/L was also used to test isolates found to have MIC values < 2 mg/L. Bacterial isolates, from a fresh overnight plate culture, were resuspended in saline and adjusted to a starting concentration equivalent to a 0.5 McF solution, then diluted 1:100 in CaMH broth. In a 1:1 ratio, 100µl of the bacterial suspension was added to each well, and 200µl to an empty growth control well. Microtiter plates were incubated for 24 hours in a static condition at 37°C. The MIC was interpreted as the well of lowest concentration with no visible growth.
To assess viability post CHX exposure (approximate MBC determination), after the MIC was read 10µl from each well in the microtiter plate was sub-cultured to a CaMH agar plate (Oxoid). No CHX inhibitors were added. Plates were incubated for 24 hours at 37°C. The MBC was interpreted as the lowest concentration in which no growth was observed, growth being defined as ≥ 2 colonies. All isolates were tested in biological triplicate. A fourth BMD was performed if the first three replicates were found to have a range in excess of 2 wells in the MIC, or 4 wells in the MBC. The median values were used for statistical analysis. Controls were tested regularly and a minimum of twice for every batch performed from a fresh dilution of CHX. Controls included *S. aureus* isolate ATCC29213, JKD6008 and culture-negative wells.
All statistical analyses were performed in R v3.4.2 (http://www.R-project.org/). Prior to significance testing all MIC and MBC values were transformed to a log2 scale. Comparison of MIC or MBC values between two population were conducted using a Welsh two sample t-test. Simple linear models were generated to assess temporal trends, comparing year of isolation against log transformed MIC and MBC data using the function "lm". Significance was determined as a p value ≤ 0.05.

Supplementary Results

Part A: Description of the pSK1-like plasmid variants

A description of the pSK1-like plasmid variants, as identified through long-read sequencing, has been described in the main text and Figure 2. Provided here is further detail about these plasmid structures and how the insertion sequence (IS) features, specifically the non-coding regions (NCRs), the terminal inverted repeats (TIRs), the target site duplications (TSD), and their location respective to plasmid and chromosomal genes were used to identify which pSK1-like SV was likely carried by each ST239 isolate.

SV1: Absence of Tn4001. In pSK1, Tn*4001* is flanked by inverted copies of IS*256* with perfect 8 bp TSD (TAAGTAAA). In SV1 only a single copy of this TSD sequence was identified in isolation from an IS*256* NCR at the expected plasmid location for Tn*4001* (Figure 2A). Tn*4001* is highly mobile and has been found at multiple chromosomal and plasmid locations (36, 37). In BPH2056 (SV1 representative genome), a chromosomal copy of Tn*4001* was identified, flanked by perfect 8 bp TSD (CAAAATCA), located downstream of a hypothetical protein in close proximity to the DNA-directed DNA polymerase III alpha subunit (*dnaE*) gene. Using this variation in TSD patterns flanking Tn*4001*, it was identified that of the ten short-read assemblies which demonstrated an orthologue pattern consistent with SV1 (Figure S1), five likely carried a plasmid consistent with pSK1 (with Tn*4001* located in the plasmid), and the other five likely carried a plasmid consistent with SV1, with a chromosomal copy of Tn*4001* identified in four isolates.

SV2: IS256-Mediated Chromosomal Integration & Loss of 5,175 bp. Chromosomal integration of SV2 was mediated by IS*256*, with two inverted copies flanking the 24,299 bp region, termed IS*256*-R and IS*256*-2. A third copy (IS*256*-L) is present as part of Tn*4001* (Figure 2B). Additionally, SV2 has a 5,175 bp deletion with loss of six genes when compared to pSK1 (Figure 2B). The upstream TSD for IS*256*-2 and IS*256*-R (external to SV2) were identical and represented a novel sequence (GAGTTGAC). A second novel TSD was identified downstream of IS*256*-2 (internal to SV2, ATGATTTC), with a single copy of the same sequence identified in pSK1 (not adjacent to an IS*256* NCR) at the boundary of the region absent in SV2 (Figure 2B). This TSD pattern was identified in all seven short-read assemblies which demonstrated an orthologue pattern consistent with SV2, suggesting that they all carried this integrated plasmid variant.

SV3: IS257-Mediated Deletion of Tn4003 & IS256-Mediated Inversion. In pSK1, Tn*4003* is flanked by direct copies of IS*257* with perfect 8 bp TSD (TTTTATAA). In SV3, only a single copy of IS*257* was identified, flanked by the same TSD (Figure 2A). Additionally, SV3 also had a 3,488 bp inversion compared to pSK1, flanked by IS*256*-R and IS256-3 (Figure 2A). The upstream TSD for IS*256*-R (internal to the inversion) and the downstream TSD for IS*256*-3 (external to the inversion) represented a novel sequence (TTTGAGTT), the former was identified in the reverse complement. A single copy of the same sequence (TTTGAGTT) was identified at the boundary for the inverted segment in pSK1 (Figure 2A, indicated by the dark red circle). The upstream TSD for IS*256*-3 (internal to the inversion, TTTACTTA) was the

same sequence as the upstream TSD for IS*256*-R in pSK1 (TAAGTAAA), but in the reverse complement (Figure 2A). Utilising the variation in TSD pattern, including the change in sequence orientation and adjacent plasmids genes, it was identified that only seven of the eleven short-read assemblies which demonstrated an orthologue pattern consistent with SV3 were likely to carry this plasmid variant.

SV4: IS256-Mediated Chromosomal Integration & Loss of 3,488 bp. Chromosomal integration of SV4 was mediated by IS*256*, with two inverted copies flanking the 22,047 bp region. A 3,488 bp region, specifically the region inverted in SV3 was absent (Figure 2B). All internal TSDs where found to correspond with those identified in SV3. The TSD upstream of IS*256*-R and IS*256*-3 (external to SV4) were novel (CTTTGTAT) (Figure 2B). This TSD pattern was identified in all four short-read assemblies which demonstrated an orthologue pattern consistent with SV4.

SV5': IS257-Mediated Chromosomal Integration & Loss of 404 bp, **SV5:** ***IS257-Mediated Inversion & Disruption of Replication Machinery & SV6: IS256-Mediated Deletion of 5,105 bp.*** A hypothesised structure has been developed for SV5', which represents an intermediate structure in the development of SV5 and SV6 (Figure 2). Chromosomal integration in SV5' would likely be mediated by IS*257*, inserting 9,233 bp upstream of a disrupted copy of the β-haemolysin gene. For compatibility with the recovered SV5 structure, this region would be flanked by IS*257*-R1 and IS*257*-R2 from pSK1, located in the same orientation, with absence of the intervening 404 bp region (Figure 2C). All TSD that are internal to SV5' would be unaltered from the sequences identified in pSK1. The TSD downstream of IS*257*-R2 and IS*257*-R1 (external to SV5') would represent a novel perfect 8 bp TSD (GTTTTTAC).

In SV5, the intermediate SV5' has undergone a 42,660 bp inversion (Figure 2C). This would likely be the result of intramolecular replicative transposition in the inverse orientation (38), and result in the creation of a novel pair of 8 bp TSD (TTCTTATC), located adjacent (in opposite orientations) to both copies of IS*257*-R2*, one external to the inverted region (adjacent to the unaffected region of φSa3) and the other one internal (adjacent to the inverted region of φSa3) (Figure 2C). In addition to the inversion, a 45 bp deletion in *repA* was detected in SV5, causing fragmentation of the gene annotation.

In a separate event, the intermediated SV5' structure has undergone an IS*256*-mediated 5,105 bp deletion event, resulting in the 22,520 bp structure SV6 (Figure 2B). All TSD are identical to those presented in the hypothesised SV5' except for one; the upstream TSD for IS*256*-R (AAATTGTG) is novel. A single copy of this TSD was identified in pSK1, not adjacent to an IS*256* NCR at the boundary for the deletion (Figure 2A).

Of the remaining 63 short-read assemblies, four had an orthologue pattern consistent with SV3, 21 with SV5, and 38 with SV6. Using the variation in TSD patterns described above, it was identified that 24 isolates likely harboured an integrated plasmid consistent with SV5', three with the inverted SV5, and 36 with the shortened SV6. All 63 isolates formed a single monophyletic clade from the expected ancestral node in which SV5' is predicted to have emerged (Figure 3). Further, these classifications were completely congruent with the

phylogenetic tree, consistent with the proposed development of these plasmid variants (Figure 3). All isolates harbouring an SV5'-like or SV5-like plasmid variant appeared to have disrupted plasmid replication machinery, the large majority demonstrating a fragmented *repA* gene annotation (Figure S1). Two isolates had more extensive deletions, with loss of multiple genes similar to SV6 (Figure 3 & Figure S1). These two isolates appear to represent two further independent IS*256*-mediated deletion events that have occurred following chromosomal integration. Similarly, four isolates appeared to have undergone an IS*257*-mediated deletion of Tn*4003*, with their location in the phylogeny suggestive of two independent events (Figure 3 & Figure S1).

Part B: Investigation of the association between plasmid evolution and biocide tolerance

Provided here are the additional investigations undertaken to explore the potential association between the emergence of the pSK1-like plasmid SVs and the development of chlorhexidine tolerance in the ST239 population.

Mutations in qacAR. A comparison of all *qacAR* sequences (n = 89 pSK1-like, n = 67 pTW20_1-like) to reference JKD6008 (Australian clade) identified six SNPs resulting in missense mutations: five in *qacA* and one in *qacR*. The most common mutation resulted in a QacA $T_{358}I$ amino acid (aa) change. This SNP was identified in all 67 *qacA*-containing Asian-Australian clade isolates and none of the *qacA*-containing Australian clade isolates. As this mutation was invariably associated with one plasmid type, irrespective of MIC, it represented the distinct evolutionary histories of the *qacA* genes in these different plasmid populations and was unlikely to be contributing to phenotypic variation. The only other two commonly observed mutations were identified in the Australian clade and resulted in a QacA $H_{164}Q$ or a QacA $V_{408}I$ aa change. These mutations were phylogenetically correlated, QacA $H_{164}Q$ was identified in 14/18 isolates that harboured SV2, SV3 or SV4, and QacA $V_{408}I$ was identified in 8/13 isolates that harboured SV3 or SV4. The presence of these mutations did not correlate with CHX phenotype, suggesting that these SNPs were most likely markers of clonal evolution and not significant contributors to the larger shift in CHX tolerance.

Plasmid and qacA copy number. Intermediate sized staphylococcal plasmids like pSK1 are generally considered to be low copy number plasmids (1). Multiple copies of *qacA*, resulting from increased plasmid or gene copy number could potential be contributing to the phenotypic variation observed between the Australian and Asian-Australian clades. To investigate this, *qacA* gene copy number was estimated by comparing the mapped sequence read depth of contigs representing the plasmid regions (as defined in Figure S2) to those representing the chromosome. The ratio of mapped-reads in the plasmid contigs representing region 4 (containing *qacAR*) and region 1 (plasmid backbone) were 0.89 (± 0.18) in the pSK1-like population and 1.12 (± 0.41) in the pBPH2003-like (pTW20_1-like) population. This suggested that each plasmid copy contained only a single copy of *qacA*. The ratio of mapped-reads in these same contigs compared to contigs representing the chromosome (specifically those containing *dnaA*) were 0.68 (± 0.28) and 0.77 (± 0.24) in the pSK1-like population, and 2.81 (± 1.32) and 2.69 (± 1.33) in the pBPH2003-like population for region 4 and region 1

respectively. These ratios suggested that the pSK1-like plasmids were typically single copy in the Australian clade isolates, consistent with most isolates harbouring a chromosomally integrated variant. No significant differences were observed between isolates that harboured an extra-chromosomal or integrated pSK1-like variant. Conversely, the Asian-Australian isolates carried one to four copies of a pBPH2003-like plasmid. As this clade has a lower average MIC to chlorhexidine (Table 2), plasmid copy number did not appear to be contributing to the variation observed in phenotypic CHX susceptibility.

Discriminant Analysis of Principle Components. DAPC is a multivariate technique that can be used to reconstruct population subdivisions hypothesised to be present using genomic data; it finds the smallest number of principle components to build discriminant functions that minimise within group variation and maximise between group variation (39). This approach was utilised to identify potential associations between phenotypic CHX MIC and the presence and/or absence of specific genes. In this analysis the population subdivisions hypothesised to be present in the dataset were the CHX MIC values (1, 1.5, 2, 3, 4, and 6 mg/L). Two models were generated: (i) one dataset representing all accessory genes identified in the ST239 collection (n = 212), and (ii) the same dataset with the pSK1 and pBPH2003 (pTW20_1-like) genes excluded.

In the first model, broad clusters were able to be resolved that reflected the low (1, 1.5, and 2 mg/L), medium (3 mg/L) and high (4 and 6 mg/L) MIC values (Figure S4A, B, C). The top contributing variables in this separation were *qacAR* and the co-located genes in plasmid region 4 in the first discriminant function, and the genes specific to pBPH2003 in the second discriminant function (Figure S4D). This finding is consistent with the increase in MIC that was associated with plasmid acquisition and the distinct CHX susceptibility profiles of the pSK1-like and pBPH2003-like populations (Table 2). Furthermore, as all but one of the *qacA*-negative isolates belonged to the Australian clade, the second discriminant function was also reflective of the phylogenetic division in the ST239 population (Figure S4E).

In the second model the MIC-reflective clusters were less defined (Figure S4A, B, C). There was some separation based on chromosome-associated gene orthologues, none of which appeared to be associated with efflux systems based on gene annotations (not shown). The membership profiles of the isolates in this model (a measure of assignment probability to the prior hypothesised subdivisions) appeared to be strongly correlated with the phylogeny (Figure S4E), which suggested that this DAPC model was detecting the underlying phylogenetic divisions within the population, which correlated with both CHX MIC and emergence of the pSK1-like plasmid variants.

Genome Wide Association Studies. A GWAS analysis was utilised to explore whether mutation in the core genome were associated with CHX MIC. This dataset was tested at three different phenotypic divisions, with the tolerant population defined as a MIC $\geq$ 3, $\geq$ 4 or 6 mg/L. Numerous mutations were identified as significantly associated with MIC after correcting for multiple hypothesis testing (Figure S5A). Examination of the 50 most significant SNPs for each phenotypic division found all to be strongly correlated with the phylogenetic model for the ST239 population (Figure S5B). GWAS approaches are highly susceptible to

phylogenetically correlated phenotypes and even if a phylogenetic penalty was applied, it may not be possible to detect a SNP in this dataset that is responsible for increased CHX MICs using this approach.

The efflux system encoded by *norA* can actively export CHX and mutations in this gene, and a homologue *norB*, have been associated with enhanced CHX tolerance (40-42). Mutations were detected in both *norA* and *norB* in this dataset, however none were significantly associated with CHX MIC in any phenotypic division tested.

Supplementary Tables

Table S1. Investigation of Gentamicin Resistance in Australian ST239 MRSA

Isolate Populations (n)	Median (mg/L) MIC		Isolate Populations (n)	Median (mg/L) MIC	*P* value
All ST239 (211)					
**Australian Clade (123)**	10.30	vs	**Asian-Australian Clade (88)**	179.80	< 0.0001
**No AME (91)**	0.38	vs	***aac(6')-aph(2")* (181)**	58.20	< 0.0001
		vs	***aadD* (73)**	15.20	< 0.0001
		vs	***aph(3')-III* (95)**	190.50	< 0.0001
Australian Clade (123)					
**No AME (19)**	0.39	vs	***aac(6')-aph(2")* (99)**	21.90	< 0.0001
		vs	***aadD* (73)**	15.20	< 0.0001
		vs	***aph(3')-III* (8)**	164.90	< 0.0001
Asian-Australian Clade (88)					
**No AME (1)[a]**	0.38	vs	***aac(6')-aph(2")* (82)**	189.70	-
		vs	***aph(3')-III* (87)**	193.00	-

Abbreviations: AME, aminoglycoside modifying enzyme; MIC, minimum inhibitory concentration.
[a] Statistical analysis was not performed as only a single isolate was identified harbouring no AME genes.

Table S2. Gentamicin Resistance and Chlorhexidine Tolerance Profiles

	Median (mg/L)		
**SK1 Plasmids (n)**	**Gentamicin MIC**	**Chlorhexidine MIC**	**Chlorhexidine MBC**
Structural Variants			
**No SK1 Plasmid (32)**	1.80	1.80	4.60
**SV1 (5)**	142.10*	2.80	7.30
**pSK1 (5)**	9.60	2.40	6.20
**SV2 (7)**	9.20	3.30	7.30
**SV3 (7)**	15.90	4.40	9.70
**SV4 (4)**	15.30	4.90	10.80
**SV5' (3)**	15.10	5.20	10.50
**SV5 (24)**	16.00	4.00	8.90
**SV6 (36)**	21.50	5.00	8.80
Structural Changes Grouped			
**Extra-chromosomal (17)[a]**	26.10	3.20	7.80
**Integrated + *repA* deletion (25)[b]**	19.10	3.70	8.50
**Integrated + MG deletion (49)[c]**	18.60	4.70	8.60

Abbreviations: MIC, minimum inhibitory concentration; MG, multi-gene; SV, pSK1-like structural variant.
[a] Includes all isolates classified as harbouring SV1, prototypical SK1 and SV3.
[b] Includes all isolates classified as SV5' and SV5 with a fragmented pSK1 *repA* sequence identified.
[c] Includes all isolates classified as SV2, SV4, SV5', SV5 and SV6 with a multi-gene deletion in pSK1 region 1.
* All five isolates representing SV1 carry *aph(3')-III*.

References

1. Jensen SO, Apisiridej S, Kwong SM, Yang YH, Skurray RA, Firth N. 2010. Analysis of the prototypical *Staphylococcus aureus* multiresistance plasmid pSK1. Plasmid 64:135-142.
2. Holden MTG, Lindsay JA, Corton C, Quail MA, Cockfield JD, Pathak S, Batra R, Parkhill J, Bentley SD, Edgeworth JD. 2010. Genome sequence of a recently emerged, highly transmissible, multi-antibiotic- and antiseptic-resistant variant of methicillin-resistant *Staphylococcus aureus*, sequence type 239 (TW). J Bacteriol 192:888-892.
3. Howden BP, Johnson PDR, Ward PB, Stinear TP, Davies JK. 2006. Isolates with low-level vancomycin resistance associated with persistent methicillin-resistant *Staphylococcus aureus* bacteremia. Antimicrob Agents Chemother 50:3039-3047.
4. Howden BP, Seemann T, Harrison PF, McEvoy CR, Stanton J-AL, Rand CJ, Mason CW, Jensen SO, Firth N, Davies JK, Johnson PDR, Stinear TP. 2010. Complete genome sequence of *Staphylococcus aureus* strain JKD6008, an ST239 clones of methicillin-resistant *Staphylococcus aureus* with intermediate level vancomycin resistance. J Bacteriol 192:5848-5849.
5. Baines SL, Holt KE, Schultz MB, Seemann T, Howden BO, Jensen SO, van Hal SJ, Coombs GW, Firth N, Powell DR, Stinear TP, Howden BP. 2015. Convergent adaptation in the dominant global hospital clone ST239 of methicillin-resistant Staphylococcus aureus. MBio 6:e00080.
6. Turnidge JD, Kotsanas D, Munckhof W, Roberts S, Bennett CM, Nimmo GR, Coombs GW, Murray RJ, Howden B, Johnson PD, Dowling K, Australia New Zealand Cooperative on Outcomes in Staphylococcal S. 2009. *Staphylococcus aureus* bacteraemia: a major cause of mortality in Australia and New Zealand. Med J Aust 191:368-73.
7. Holmes NE, Turnidge JD, Munckhof WJ, Robinson JO, Korman TM, O'Sullivan MVN, Anderson TL, Roberts SA, Gao W, Christiansen KJ, Coombs GW, Johnson PDR, Howden BP. 2011. Antibiotic choice may not explain poorer outcomes in patients with *Staphylococcus aureus* bacteremia and high vancomycin minimum inhibitory concentration. J Infect Dis 204:340-347.
8. Holmes NE, Robinson JO, van Hal SJ, Munckhof WJ, Athan E, Korman TM, Cheng AC, Turnidge JD, Johnson PDR, Howden BP, Vanessa study group obotASfIDCRN. 2018. Morbidity from in-hospital complications is greater than treatment failure in patients with Staphylococcus aureus bacteraemia. BMC Infect Dis 18:107.
9. Chen F-J, Lauderdale T-L, Wang L-S, Hsuang I-W. 2013. Complete genome sequence of *Staphylococcus aureus* Z172, a vancomycin-intermediate and daptomycin-nonsusceptible methicillin-resistant strain isolated in Taiwan. Genome Announc 1:e01011-13.
10. Costa MO, Beltrame CO, Ferreira FA, Botelho AM, Lima NC, Souza RC, de Almeida LG, Vasconcelos AT, Nicolas MF, Figueiredo AM. 2013. Complete Genome Sequence of a Variant of the Methicillin-Resistant Staphylococcus aureus ST239 Lineage, Strain BMB9393, Displaying Superior Ability To Accumulate ica-Independent Biofilm. Genome Announc 1.
11. Botelho AM, Costa MO, Beltrame CO, Ferreira FA, Cortes MF, Bandeira PT, Lima NC, Souza RC, Almeida LG, Vasconcelos AT, Nicolas MF, Figueiredo AM. 2016. Complete genome sequence of an agr-dysfunctional variant of the ST239 lineage of the methicillin-resistant Staphylococcus aureus strain GV69 from Brazil. Stand Genomic Sci 11:34.
12. Botelho AM, Costa MO, Beltrame CO, Ferreira FA, Lima NC, Costa BS, de Morais GL, Souza RC, Almeida LG, Vasconcelos AT, Nicolas MF, Figueiredo AM. 2016. Complete Genome Sequence of the MRSA Isolate HC1335 from ST239 Lineage Displaying a Truncated AgrC Histidine Kinase Receptor. Genome Biol Evol 8:3187-3192.
13. McClure JA, Zhang K. 2017. Complete Genome Sequence of the Methicillin-Resistant Staphylococcus aureus Colonizing Strain M92. Genome Announc 5.
14. Li Y, Cao B, Zhang Y, Zhou J, Yang B, Wang L. 2011. Complete genome sequence of *Staphylococcus aureus* T0131, an ST239-MRSA-SCC*mec* type III clones isolated in China. J Bacteriol 193:3411-3412.

15. Zhang X, Xu X, Yuan W, Hu Q, Shang W, Hu X, Tong Y, Rao X. 2014. Complete Genome Sequence of Staphylococcus aureus XN108, an ST239-MRSA-SCCmec III Strain with Intermediate Vancomycin Resistance Isolated in Mainland China. Genome Announc 2.
16. Alam MT, Petit RA, 3rd, Crispell EK, Thornton TA, Conneely KN, Jiang Y, Satola SW, Read TD. 2014. Dissecting vancomycin-intermediate resistance in staphylococcus aureus using genome-wide association. Genome Biol Evol 6:1174-85.
17. Castillo-Ramirez S, Corander J, Marttinen P, Aldeljawi M, Hanage WP, Westh H, Boye K, Bentley SD, Parkhill J, Holden MT, Feil EJ. 2012. Phylogeographic variation in recombination rates within a global clone of methicillin-resistant *Staphylococcus aureus*. Genome Biol 13:R126.
18. Harris SR, Feil EJ, Holden MTG, Quail MA, Nickerson EK, Chantratita N, Gardete S, Tavares A, Day N, Lindsay JA, Edgeworth JD, de Lencastre H, Parkhill J, Peacock SJ, Bentley SD. 2010. Evolution of MRSA during hospital transmission and intercontinental spread. Science 327:469-474.
19. Kong Z, Zhao P, Liu H, Yu X, Qin Y, Su Z, Wang S, Xu H, Chen J. 2016. Whole-Genome Sequencing for the Investigation of a Hospital Outbreak of MRSA in China. PLoS One 11:e0149844.
20. Moradigaravand D, Jamrozy D, Mostowy R, Anderson A, Nickerson EK, Thaipadungpanit J, Wuthiekanun V, Limmathurotsakul D, Tandhavanant S, Wikraiphat C, Wongsuvan G, Teerawattanasook N, Jutrakul Y, Srisurat N, Chaimanee P, Eoin West T, Blane B, Parkhill J, Chantratita N, Peacock SJ. 2017. Evolution of the Staphylococcus argenteus ST2250 Clone in Northeastern Thailand Is Linked with the Acquisition of Livestock-Associated Staphylococcal Genes. MBio 8.
21. Reuter S, Torok ME, Holden MT, Reynolds R, Raven KE, Blane B, Donker T, Bentley SD, Aanensen DM, Grundmann H, Feil EJ, Spratt BG, Parkhill J, Peacock SJ. 2016. Building a genomic framework for prospective MRSA surveillance in the United Kingdom and the Republic of Ireland. Genome Res 26:263-70.
22. Senghore M, Bayliss SC, Kwambana-Adams BA, Foster-Nyarko E, Manneh J, Dione M, Badji H, Ebruke C, Doughty EL, Thorpe HA, Jasinska AJ, Schmitt CA, Cramer JD, Turner TR, Weinstock G, Freimer NB, Pallen MJ, Feil EJ, Antonio M. 2016. Transmission of Staphylococcus aureus from Humans to Green Monkeys in The Gambia as Revealed by Whole-Genome Sequencing. Appl Environ Microbiol 82:5910-7.
23. Hsu LY, Harris SR, Chlebowicz MA, Lindsay JA, Koh TH, Krishnan P, Tan TY, Hon PY, Grubb WB, Bentley SD, Parkhill J, Peacock SJ, Holden MT. 2015. Evolutionary dynamics of methicillin-resistant Staphylococcus aureus within a healthcare system. Genome Biol 16:81.
24. Tong SY, Holden MT, Nickerson EK, Cooper BS, Koser CU, Cori A, Jombart T, Cauchemez S, Fraser C, Wuthiekanun V, Thaipadungpanit J, Hongsuwan M, Day NP, Limmathurotsakul D, Parkhill J, Peacock SJ. 2015. Genome sequencing defines phylogeny and spread of methicillin-resistant Staphylococcus aureus in a high transmission setting. Genome Res 25:111-8.
25. Baines SL, Howden BP, Heffernan H, Stinear TP, Carter GP, Seemann T, Kwong JC, Ritchie SR, Williamson DA. 2016. Rapid Emergence and Evolution of *Staphylococcus aureus* Clones Harboring *fusC*-Containing Staphylococcal Cassette Chromosome Elements. Antimicrob Agents Chemother 60:2359-65.
26. Carver TJ, Rutherford, K. M., Berriman, M., Rajandream, M., Barrell, B. G., Parkhill, J. 2005. ACT: the Artemis comparison tool. Bioinformatics Appl Notes 21:3422-3423.
27. Bankevich A, Nurk S, Antipov D, Gurevich AA, Dvorkin M, Kulikov AS, Lesin VM, Nikolenko SI, Pham S, Prjibelski AD, Pyshkin AV, Sirotkin AV, Vyahhi N, Tesler G, Alekseyev MA, Pevzner PA. 2012. SPAdes: a new genome assembly algorithm and its applications to single-cell sequencing. J Comput Biol 19:455-77.
28. Seemann T. 2014. Prokka: rapid prokaryotic genome annotation. Bioinformatics 30:2068-9.

29. Page AJ, Cummins CA, Hunt M, Wong VK, Reuter S, Holden MT, Fookes M, Falush D, Keane JA, Parkhill J. 2015. Roary: rapid large-scale prokaryote pan genome analysis. Bioinformatics 31:3691-3.
30. Nguyen LT, Schmidt HA, von Haeseler A, Minh BQ. 2015. IQ-TREE: a fast and effective stochastic algorithm for estimating maximum-likelihood phylogenies. Mol Biol Evol 32:268-74.
31. Didelot X, Wilson DJ. 2015. ClonalFrameML: efficient inference of recombination in whole bacterial genomes. PLoS Comput Biol 11:e1004041.
32. Bouckaert R, Heled J, Kuhnert D, Vaughan T, Wu CH, Xie D, Suchard MA, Rambaut A, Drummond AJ. 2014. BEAST 2: a software platform for Bayesian evolutionary analysis. PLoS Comput Biol 10:e1003537.
33. Gray RR, Tatem AJ, Johnson JA, Alekseyenko AV, Pybus OG, Suchard MA, Salemi M. 2011. Testing spatiotemporal hypothesis of bacterial evolution using methicillin-resistant *Staphylococcus aureus* ST239 genome-wide data within a bayesian framework. Mol Biol Evol 28:1593-1603.
34. Purcell S, Neale B, Todd-Brown K, Thomas L, Ferreira MA, Bender D, Maller J, Sklar P, de Bakker PI, Daly MJ, Sham PC. 2007. PLINK: a tool set for whole-genome association and population-based linkage analyses. Am J Hum Genet 81:559-575.
35. Jombart T, Ahmed I. 2011. adegenet 1.3-1: new tools for the analysis of genome-wide SNP data. Bioinformatics 27:3070-1.
36. Mahairas GG, Lyon BR, Skurray RA, Pattee PA. 1989. Genetic analysis of *Staphylococcus aureus* with Tn*4001*. J Bacteriol 171:3968-72.
37. Lyon BR, Gillespie MT, Skurray RA. 1987. Detection and characterization of IS256, an insertion sequence in *Staphylococcus aureus*. J Gen Microbiol 133:3031-8.
38. Partridge SR, Kwong SM, Firth N, Jensen SO. 2018. Mobile Genetic Elements Associated with Antimicrobial Resistance. Clin Microbiol Rev 31.
39. Jombart T, Devillard S, Balloux F. 2010. Discriminant analysis of principal components: a new method for the analysis of genetically structured populations. BMC Genet 11:94.
40. Hardy K, Sunnucks K, Gil H, Shabir S, Trampari E, Hawkey P, Webber M. 2018. Increased Usage of Antiseptics Is Associated with Reduced Susceptibility in Clinical Isolates of *Staphylococcus aureus*. MBio 9.
41. Paulsen IT, Brown MH, Littlejohn TG, Mitchell BA, Skurray RA. 1996. Multidrug resistance proteins QacA and QacB from *Staphylococcus aureus*: membrane topology and identification of residues involved in substrate specificity. Proc Natl Acad Sci U S A 93:3630-5.
42. Horner C, Mawer D, Wilcox M. 2012. Reduced susceptibility to chlorhexidine in staphylococci: is it increasing and does it matter? J Antimicrob Chemother 67:2547-59.

Supplementary Dataset

	ID	Year	Country	State (AUS)	Data Type	Sequence Platform	Sample/Genome Accession	Run Accession	Sequence Reference
1	**BPH0468**	2005	Australia	QLD	WGS Reads	Illumina MiSeq	SAMEA3212938	ERR732815	Baines. 2015. mBio. PMID: 25736880
2	**BPH0475**	2003	Australia	VIC	WGS Reads	Illumina MiSeq	SAMEA3212931	ERR732816	Baines. 2015. mBio. PMID: 25736880
3	**BPH0488**	2003	Australia	VIC	WGS Reads	Illumina MiSeq	SAMEA3212956	ERR732817	Baines. 2015. mBio. PMID: 25736880
4	**BPH0491**	2005	Australia	VIC	WGS Reads	Illumina MiSeq	SAMEA3212955	ERR732818	Baines. 2015. mBio. PMID: 25736880
5	**BPH0494**	2005	Australia	QLD	WGS Reads	Illumina MiSeq	SAMEA3212940	ERR732819	Baines. 2015. mBio. PMID: 25736880
6	**BPH0496**	2004	Australia	QLD	WGS Reads	Illumina MiSeq	SAMEA3212928	ERR732820	Baines. 2015. mBio. PMID: 25736880
7	**BPH0499**	2005	Australia	VIC	WGS Reads	Illumina MiSeq	SAMEA3212937	ERR732821	Baines. 2015. mBio. PMID: 25736880
8	**BPH0502**	2003	Australia	WA	WGS Reads	Illumina MiSeq	SAMEA3212919	ERR732822	Baines. 2015. mBio. PMID: 25736880
9	**BPH0503**	2003	Australia	VIC	WGS Reads	Illumina MiSeq	SAMEA3212967	ERR732823	Baines. 2015. mBio. PMID: 25736880
10	**BPH0508**	2006	Australia	QLD	WGS Reads	Illumina MiSeq	SAMEA3212893	ERR732824	Baines. 2015. mBio. PMID: 25736880
11	**BPH0509**	2005	Australia	QLD	WGS Reads	Illumina MiSeq	SAMEA3212911	ERR732825	Baines. 2015. mBio. PMID: 25736880
12	**BPH0510**	2005	Australia	NSW	WGS Reads	Illumina MiSeq	SAMEA3212957	ERR732826	Baines. 2015. mBio. PMID: 25736880
13	**BPH2002**	2002	Australia	VIC	WGS Reads	Illumina MiSeq	SAMEA3212904	ERR732827	Baines. 2015. mBio. PMID: 25736880
14	**BPH2003**	2001	Australia	VIC	WGS Reads	Illumina MiSeq	SAMEA3212913	ERR732828	Baines. 2015. mBio. PMID: 25736880
-	**pBPH2003 (BPH2003)**	-	-	-	Complete Genome	Pacific Biosciences RS-II	GCA_900607265	-	This Study
-	**pBPH2003 (BPH2003)**	-	-	-	WGS Long Reads	Pacific Biosciences RS-II	SAMEA5047398	ERR2858549	This Study
15	**BPH2004**	2001	Australia	VIC	WGS Reads	Illumina MiSeq	SAMEA3212949	ERR732829	Baines. 2015. mBio. PMID: 25736880
16	**BPH2005**	1999	Australia	VIC	WGS Reads	Illumina MiSeq	SAMEA3212961	ERR732830	Baines. 2015. mBio. PMID: 25736880
17	**BPH2006**	2001	Australia	VIC	WGS Reads	Illumina MiSeq	SAMEA3212925	ERR732831	Baines. 2015. mBio. PMID: 25736880
18	**BPH2007**	2001	Australia	VIC	WGS Reads	Illumina MiSeq	SAMEA3212952	ERR732832	Baines. 2015. mBio. PMID: 25736880
19	**BPH2008**	2000	Australia	VIC	WGS Reads	Illumina MiSeq	SAMEA3212918	ERR732833	Baines. 2015. mBio. PMID: 25736880
20	**BPH2009**	2000	Australia	VIC	WGS Reads	Illumina MiSeq	SAMEA3212948	ERR732834	Baines. 2015. mBio. PMID: 25736880
21	**BPH2010**	2001	Australia	VIC	WGS Reads	Illumina MiSeq	SAMEA3212899	ERR732835	Baines. 2015. mBio. PMID: 25736880
22	**BPH2011**	2001	Australia	VIC	WGS Reads	Illumina MiSeq	SAMEA3212962	ERR732836	Baines. 2015. mBio. PMID: 25736880
23	**BPH2012**	1999	Australia	VIC	WGS Reads	Illumina MiSeq	SAMEA3212958	ERR732837	Baines. 2015. mBio. PMID: 25736880
24	**BPH2013**	2004	Australia	VIC	WGS Reads	Illumina MiSeq	SAMEA3212934	ERR732838	Baines. 2015. mBio. PMID: 25736880
25	**BPH2014**	1999	Australia	VIC	WGS Reads	Illumina MiSeq	SAMEA3212969	ERR732839	Baines. 2015. mBio. PMID: 25736880
26	**BPH2015**	2004	Australia	VIC	WGS Reads	Illumina MiSeq	SAMEA3212954	ERR732840	Baines. 2015. mBio. PMID: 25736880
27	**BPH2016**	2005	Australia	VIC	WGS Reads	Illumina MiSeq	SAMEA3212908	ERR732841	Baines. 2015. mBio. PMID: 25736880
28	**BPH2017**	2005	Australia	VIC	WGS Reads	Illumina MiSeq	SAMEA3212936	ERR732842	Baines. 2015. mBio. PMID: 25736880
29	**BPH2018**	2004	Australia	VIC	WGS Reads	Illumina MiSeq	SAMEA3212942	ERR732843	Baines. 2015. mBio. PMID: 25736880
30	**BPH2019**	2005	Australia	VIC	WGS Reads	Illumina MiSeq	SAMEA3212951	ERR732844	Baines. 2015. mBio. PMID: 25736880
-	**SV3 (BPH2019)**	-	-	-	Complete Genome	Pacific Biosciences RS-II	GCA_900607255	-	This Study
-	**SV3 (BPH2019)**	-	-	-	WGS Long Reads	Pacific Biosciences RS-II	SAMEA5047399	ERR2858550	This Study
31	**BPH2021**	2004	Australia	VIC	WGS Reads	Illumina MiSeq	SAMEA3212903	ERR732845	Baines. 2015. mBio. PMID: 25736880
32	**BPH2022**	2005	Australia	VIC	WGS Reads	Illumina MiSeq	SAMEA3212945	ERR732846	Baines. 2015. mBio. PMID: 25736880
33	**BPH2023**	2006	Australia	VIC	WGS Reads	Illumina MiSeq	SAMEA3212915	ERR732847	Baines. 2015. mBio. PMID: 25736880
34	**BPH2024**	2007	Australia	VIC	WGS Reads	Illumina MiSeq	SAMEA3212944	ERR732848	Baines. 2015. mBio. PMID: 25736880
35	**BPH2029**	2009	Australia	VIC	WGS Reads	Illumina MiSeq	SAMEA3212905	ERR732852	Baines. 2015. mBio. PMID: 25736880
36	**BPH2031**	2008	Australia	VIC	WGS Reads	Illumina MiSeq	SAMEA3212965	ERR732853	Baines. 2015. mBio. PMID: 25736880
37	**BPH2032**	1998	Australia	VIC	WGS Reads	Illumina MiSeq	SAMEA3212926	ERR732854	Baines. 2015. mBio. PMID: 25736880
38	**BPH2034**	2008	Australia	VIC	WGS Reads	Illumina MiSeq	SAMEA3212917	ERR732856	Baines. 2015. mBio. PMID: 25736880
39	**BPH2054**	1980	Australia	VIC	WGS Reads	Illumina MiSeq	SAMEA3212883	ERR732860	Baines. 2015. mBio. PMID: 25736880
40	**BPH2055**	1980	Australia	VIC	WGS Reads	Illumina MiSeq	SAMEA3212885	ERR732861	Baines. 2015. mBio. PMID: 25736880
41	**BPH2056**	1981	Australia	VIC	WGS Reads	Illumina MiSeq	SAMEA3212902	ERR732862	Baines. 2015. mBio. PMID: 25736880
-	**SV1 (BPH2056)**	-	-	-	Complete Genome	Pacific Biosciences RS-II	GCA_900607305	-	This Study
-	**SV1 (BPH2056)**	-	-	-	WGS Long Reads	Pacific Biosciences RS-II	SAMEA5047400	ERR2858551	This Study
42	**BPH2057**	1981	Australia	VIC	WGS Reads	Illumina MiSeq	SAMEA3212889	ERR732863	Baines. 2015. mBio. PMID: 25736880

43	**BPH2059**	1981	Australia	VIC	WGS Reads	Illumina MiSeq	SAMEA3212897	ERR732864	Baines. 2015. mBio. PMID: 25736880
44	**BPH2068**	2009	Australia	VIC	WGS Reads	Illumina MiSeq	SAMEA3212906	ERR732865	Baines. 2015. mBio. PMID: 25736880
45	**BPH2070**	1998	Australia	VIC	WGS Reads	Illumina MiSeq	SAMEA3212890	ERR732866	Baines. 2015. mBio. PMID: 25736880
-	**SV2 (BPH2070)**	-	-	-	Complete Genome	Pacific Biosciences RS-II	GCA_900607275	-	This Study
-	**SV2 (BPH2070)**	-	-	-	WGS Long Reads	Pacific Biosciences RS-II	SAMEA5047401	ERR2858552	This Study
46	**BPH2071**	1998	Australia	VIC	WGS Reads	Illumina MiSeq	SAMEA3212888	ERR732867	Baines. 2015. mBio. PMID: 25736880
47	**BPH2072**	1982	Australia	NSW	WGS Reads	Illumina MiSeq	SAMEA3212947	ERR732868	Baines. 2015. mBio. PMID: 25736880
48	**BPH2073**	1982	Australia	NSW	WGS Reads	Illumina MiSeq	SAMEA3212927	ERR732869	Baines. 2015. mBio. PMID: 25736880
49	**BPH2075**	1982	Australia	NSW	WGS Reads	Illumina MiSeq	SAMEA3212896	ERR732870	Baines. 2015. mBio. PMID: 25736880
50	**BPH2077**	1982	Australia	NSW	WGS Reads	Illumina MiSeq	SAMEA3212932	ERR732871	Baines. 2015. mBio. PMID: 25736880
51	**BPH2078**	1982	Australia	NSW	WGS Reads	Illumina MiSeq	SAMEA3212950	ERR732872	Baines. 2015. mBio. PMID: 25736880
52	**BPH2079**	1982	Australia	NSW	WGS Reads	Illumina MiSeq	SAMEA3212922	ERR732873	Baines. 2015. mBio. PMID: 25736880
53	**BPH2080**	1982	Australia	NSW	WGS Reads	Illumina MiSeq	SAMEA3212929	ERR732874	Baines. 2015. mBio. PMID: 25736880
54	**BPH2081**	1982	Australia	NSW	WGS Reads	Illumina MiSeq	SAMEA3212941	ERR732875	Baines. 2015. mBio. PMID: 25736880
55	**BPH2082**	1982	Australia	NSW	WGS Reads	Illumina MiSeq	SAMEA3212920	ERR732876	Baines. 2015. mBio. PMID: 25736880
56	**BPH2083**	1982	Australia	NSW	WGS Reads	Illumina MiSeq	SAMEA3212886	ERR732877	Baines. 2015. mBio. PMID: 25736880
57	**BPH2084**	1982	Australia	NSW	WGS Reads	Illumina MiSeq	SAMEA3212892	ERR732878	Baines. 2015. mBio. PMID: 25736880
58	**BPH2085**	1982	Australia	NSW	WGS Reads	Illumina MiSeq	SAMEA3212907	ERR732879	Baines. 2015. mBio. PMID: 25736880
59	**BPH2086**	1982	Australia	NSW	WGS Reads	Illumina MiSeq	SAMEA3212900	ERR732880	Baines. 2015. mBio. PMID: 25736880
60	**BPH2088**	1982	Australia	NSW	WGS Reads	Illumina MiSeq	SAMEA3212968	ERR732881	Baines. 2015. mBio. PMID: 25736880
61	**BPH2090**	2012	Australia	VIC	WGS Reads	Illumina MiSeq	SAMEA3212943	ERR732882	Baines. 2015. mBio. PMID: 25736880
62	**BPH2094**	1998	Australia	NSW	WGS Reads	Ion Torrent PGM	SAMEA3212946	ERR732883	Baines. 2015. mBio. PMID: 25736880
63	**BPH2095**	2003	Australia	NSW	WGS Reads	Ion Torrent PGM	SAMEA3212887	ERR732884	Baines. 2015. mBio. PMID: 25736880
64	**BPH2096**	2005	Australia	NSW	WGS Reads	Ion Torrent PGM	SAMEA3212923	ERR732885	Baines. 2015. mBio. PMID: 25736880
65	**BPH2097**	2007	Australia	NSW	WGS Reads	Ion Torrent PGM	SAMEA3212939	ERR732886	Baines. 2015. mBio. PMID: 25736880
66	**BPH2098**	2007	Australia	NSW	WGS Reads	Ion Torrent PGM	SAMEA3212884	ERR732887	Baines. 2015. mBio. PMID: 25736880
67	**BPH2099**	2007	Australia	NSW	WGS Reads	Ion Torrent PGM	SAMEA3212933	ERR732888	Baines. 2015. mBio. PMID: 25736880
68	**JKD6000**	2002	Australia	VIC	WGS Reads	Illumina Genome Analyzer IIx	SAMEA3212909	ERR732889	Baines. 2015. mBio. PMID: 25736880
69	**JKD6004**	2002	Australia	QLD	WGS Reads	Illumina Genome Analyzer IIx	SAMEA3212901	ERR732891	Baines. 2015. mBio. PMID: 25736880
70	**JKD6008**	2006	New Zealand	-	Complete Genome	Roache 454 GS20, SOLiD	CP002120	-	Howden. 2010. JBact. PMID:20802046
71	**JKD6009**	2003	New Zealand	-	WGS Reads	Illumina Genome Analyzer IIx	SAMEA3212894	ERR732894	Baines. 2015. mBio. PMID: 25736880
-	**SV5 (JKD6009)**	-	-	-	Complete Genome	Pacific Biosciences RS-II	GCA_900607245	-	This Study
-	**SV5 (JKD6009)**	-	-	-	WGS Long Reads	Pacific Biosciences RS-II	SAMEA5047402	ERR2858553	This Study
72	**JKD6021**	2001	Australia	VIC	WGS Reads	Illumina Genome Analyzer IIx	SAMEA3212953	ERR732895	Baines. 2015. mBio. PMID: 25736880
73	**JKD6052**	2004	Australia	QLD	WGS Reads	Illumina Genome Analyzer IIx	SAMEA3212916	ERR732899	Baines. 2015. mBio. PMID: 25736880
74	**JKD6121**	2005	Australia	VIC	WGS Reads	Illumina Genome Analyzer IIx	SAMEA3212963	ERR732900	Baines. 2015. mBio. PMID: 25736880
75	**BPH2065**	1981	Australia	SA	WGS Reads	Illumina NextSeq 500	SAMEA5042664	ERR2855733	This Study
76	**BPH2066**	1981	Australia	SA	WGS Reads	Illumina NextSeq 500	SAMEA5042665	ERR2855734	This Study
77	**BPH2104**	1990	Australia	VIC	WGS Reads	Illumina NextSeq 500	SAMEA5042666	ERR2855735	This Study
78	**BPH2106**	1992	Australia	VIC	WGS Reads	Illumina NextSeq 500	SAMEA5042667	ERR2855736	This Study
79	**BPH2107**	1990	Australia	VIC	WGS Reads	Illumina NextSeq 500	SAMEA5042669	ERR2855737	This Study
80	**BPH2154**	1992	Australia	VIC	WGS Reads	Illumina NextSeq 500	SAMEA5042676	ERR2855744	This Study
81	**BPH2161**	1992	Australia	VIC	WGS Reads	Illumina NextSeq 500	SAMEA5042678	ERR2855746	This Study
82	**BPH2163**	1989	Australia	VIC	WGS Reads	Illumina NextSeq 500	SAMEA5042679	ERR2855747	This Study
83	**BPH2165**	1989	Australia	VIC	WGS Reads	Illumina NextSeq 500	SAMEA5042680	ERR2855748	This Study
84	**BPH2203**	1992	Australia	VIC	WGS Reads	Illumina NextSeq 500	SAMEA5042687	ERR2855755	This Study
85	**BPH2205**	1989	Australia	VIC	WGS Reads	Illumina NextSeq 500	SAMEA5042689	ERR2855757	This Study
86	**BPH2226**	1990	Australia	VIC	WGS Reads	Illumina NextSeq 500	SAMEA5042695	ERR2855763	This Study
87	**BPH2228**	1990	Australia	QLD	WGS Reads	Illumina NextSeq 500	SAMEA5042696	ERR2855764	This Study
88	**BPH2233**	1990	Australia	VIC	WGS Reads	Illumina NextSeq 500	SAMEA5042699	ERR2855767	This Study
89	**BPH2234**	1991	Australia	VIC	WGS Reads	Illumina NextSeq 500	SAMEA5042700	ERR2855768	This Study

90	**BPH2235**	1991	Australia	VIC	WGS Reads	Illumina NextSeq 500	SAMEA5042701	ERR2855769	This Study
91	**BPH2236**	1991	Australia	VIC	WGS Reads	Illumina NextSeq 500	SAMEA5042702	ERR2855770	This Study
92	**BPH2238**	1989	Australia	VIC	WGS Reads	Illumina NextSeq 500	SAMEA5042703	ERR2855771	This Study
93	**BPH2710**	2011	Australia	VIC	WGS Reads	Illumina MiSeq	SAMEA104317322	ERR2137022	Publication in process
94	**BPH2718**	2011	Australia	VIC	WGS Reads	Illumina MiSeq	SAMEA104317330	ERR2137030	Publication in process
95	**BPH2723**	2011	Australia	VIC	WGS Reads	Illumina MiSeq	SAMEA104317335	ERR2137035	Publication in process
96	**BPH2726**	2011	Australia	VIC	WGS Reads	Illumina MiSeq	SAMEA104317338	ERR2137038	Publication in process
97	**BPH2747**	2011	Australia	VIC	WGS Reads	Illumina MiSeq	SAMEA104317359	ERR2137059	Publication in process
98	**BPH2779**	2011	Australia	VIC	WGS Reads	Illumina HiSeq	SAMEA104317390	ERR2137090	Publication in process
99	**BPH2780**	2011	Australia	VIC	WGS Reads	Illumina HiSeq	SAMEA104317391	ERR2137091	Publication in process
100	**BPH2781**	2011	Australia	VIC	WGS Reads	Illumina HiSeq	SAMEA104317392	ERR2137092	Publication in process
101	**BPH2787**	2011	Australia	NSW	WGS Reads	Illumina MiSeq	SAMEA104317398	ERR2137098	Publication in process
102	**BPH2798**	2011	Australia	VIC	WGS Reads	Illumina HiSeq	SAMEA104317409	ERR2137109	Publication in process
103	**BPH2824**	2011	Australia	VIC	WGS Reads	Illumina HiSeq	SAMEA104317431	ERR2137131	Publication in process
104	**BPH2827**	2011	Australia	NSW	WGS Reads	Illumina MiSeq	SAMEA104317434	ERR2137134	Publication in process
105	**BPH2869**	2012	Australia	NSW	WGS Reads	Illumina MiSeq	SAMEA104317472	ERR2137172	Publication in process
-	**SV6 (BPH2869)**	-	-	-	Complete Genome	Pacific Biosciences RS-II	GCA_900607295	-	This Study
-	**SV6 (BPH2869)**	-	-	-	WGS Long Reads	Pacific Biosciences RS-II	SAMEA104317472	ERR2858554	This Study
106	**BPH2896**	2012	Australia	VIC	WGS Reads	Illumina MiSeq	SAMEA104317497	ERR2137197	Publication in process
107	**BPH2946**	2012	Australia	VIC	WGS Reads	Illumina MiSeq	SAMEA104317543	ERR2137243	Publication in process
108	**BPH2947**	2012	Australia	VIC	WGS Reads	Illumina MiSeq	SAMEA104317544	ERR2137244	Publication in process
109	**BPH3202**	2008	Australia	QLD	WGS Reads	Illumina NextSeq 500	SAMEA104317594	ERR2137294	Publication in process
110	**BPH3208**	2007	Australia	QLD	WGS Reads	Illumina NextSeq 500	SAMEA104317600	ERR2137300	Publication in process
111	**BPH3220**	2008	Australia	QLD	WGS Reads	Illumina NextSeq 500	SAMEA104317612	ERR2137312	Publication in process
112	**BPH3234**	2008	Australia	QLD	WGS Reads	Illumina NextSeq 500	SAMEA104317626	ERR2137326	Publication in process
113	**BPH3244**	2007	Australia	QLD	WGS Reads	Illumina NextSeq 500	SAMEA104317636	ERR2137336	Publication in process
-	**SV4 (BPH3244)**	-	-	-	Complete Genome	Pacific Biosciences RS-II	GCA_900607285	-	This Study
-	**SV4 (BPH3244)**	-	-	-	WGS Long Reads	Pacific Biosciences RS-II	SAMEA104317636	ERR2858555	This Study
114	**BPH3250**	2007	Australia	QLD	WGS Reads	Illumina NextSeq 500	SAMEA104317642	ERR2137342	Publication in process
115	**BPH3254**	2008	Australia	QLD	WGS Reads	Illumina NextSeq 500	SAMEA104317646	ERR2137346	Publication in process
116	**BPH3260**	2007	Australia	QLD	WGS Reads	Illumina NextSeq 500	SAMEA104317652	ERR2137352	Publication in process
117	**BPH3262**	2008	Australia	QLD	WGS Reads	Illumina NextSeq 500	SAMEA104317654	ERR2137354	Publication in process
118	**BPH3266**	2008	Australia	QLD	WGS Reads	Illumina NextSeq 500	SAMEA104317658	ERR2137358	Publication in process
119	**BPH3272**	2007	Australia	QLD	WGS Reads	Illumina NextSeq 500	SAMEA104317664	ERR2137364	Publication in process
120	**BPH3276**	2008	Australia	QLD	WGS Reads	Illumina NextSeq 500	SAMEA104317668	ERR2137368	Publication in process
121	**BPH3284**	2008	Australia	QLD	WGS Reads	Illumina NextSeq 500	SAMEA104317676	ERR2137376	Publication in process
122	**BPH3306**	2007	Australia	QLD	WGS Reads	Illumina NextSeq 500	SAMEA104317698	ERR2137398	Publication in process
123	**BPH3324**	2008	Australia	VIC	WGS Reads	Illumina NextSeq 500	SAMEA104317716	ERR2137416	Publication in process
124	**BPH3328**	2008	Australia	VIC	WGS Reads	Illumina NextSeq 500	SAMEA104317720	ERR2137420	Publication in process
125	**BPH3332**	2008	Australia	VIC	WGS Reads	Illumina NextSeq 500	SAMEA104317724	ERR2137424	Publication in process
126	**BPH3344**	2008	Australia	VIC	WGS Reads	Illumina NextSeq 500	SAMEA104317736	ERR2137436	Publication in process
127	**BPH3346**	2008	Australia	VIC	WGS Reads	Illumina NextSeq 500	SAMEA104317738	ERR2137438	Publication in process
128	**BPH3348**	2007	Australia	VIC	WGS Reads	Illumina NextSeq 500	SAMEA104317740	ERR2137440	Publication in process
129	**BPH3356**	2007	Australia	VIC	WGS Reads	Illumina NextSeq 500	SAMEA104317748	ERR2137448	Publication in process
130	**BPH3360**	2008	Australia	VIC	WGS Reads	Illumina NextSeq 500	SAMEA104317752	ERR2137452	Publication in process
131	**BPH3362**	2008	Australia	VIC	WGS Reads	Illumina NextSeq 500	SAMEA104317754	ERR2137454	Publication in process
132	**BPH3366**	2008	Australia	VIC	WGS Reads	Illumina NextSeq 500	SAMEA104317758	ERR2137458	Publication in process
133	**BPH3368**	2008	Australia	VIC	WGS Reads	Illumina NextSeq 500	SAMEA104317760	ERR2137460	Publication in process
134	**BPH3370**	2008	Australia	VIC	WGS Reads	Illumina NextSeq 500	SAMEA104317762	ERR2137462	Publication in process
135	**BPH3372**	2008	Australia	VIC	WGS Reads	Illumina NextSeq 500	SAMEA104317764	ERR2137464	Publication in process
136	**BPH3376**	2007	Australia	VIC	WGS Reads	Illumina NextSeq 500	SAMEA104317768	ERR2137468	Publication in process

137	**BPH3382**	2008	Australia	VIC	WGS Reads	Illumina NextSeq 500	SAMEA104317774	ERR2137474	Publication in process
138	**BPH3394**	2007	Australia	VIC	WGS Reads	Illumina NextSeq 500	SAMEA104317784	ERR2137484	Publication in process
139	**BPH3401**	2007	Australia	VIC	WGS Reads	Illumina NextSeq 500	SAMEA104317791	ERR2137491	Publication in process
140	**BPH3403**	2007	Australia	VIC	WGS Reads	Illumina NextSeq 500	SAMEA104317793	ERR2137493	Publication in process
141	**BPH3413**	2007	Australia	VIC	WGS Reads	Illumina NextSeq 500	SAMEA104317802	ERR2137502	Publication in process
142	**BPH3415**	2007	Australia	VIC	WGS Reads	Illumina NextSeq 500	SAMEA104317804	ERR2137504	Publication in process
143	**BPH3417**	2007	Australia	VIC	WGS Reads	Illumina NextSeq 500	SAMEA104317806	ERR2137506	Publication in process
144	**BPH3431**	2007	Australia	VIC	WGS Reads	Illumina NextSeq 500	SAMEA104317820	ERR2137520	Publication in process
145	**BPH3433**	2008	Australia	VIC	WGS Reads	Illumina NextSeq 500	SAMEA104317822	ERR2137522	Publication in process
146	**BPH3435**	2007	Australia	VIC	WGS Reads	Illumina NextSeq 500	SAMEA104317824	ERR2137524	Publication in process
147	**BPH3439**	2007	Australia	VIC	WGS Reads	Illumina NextSeq 500	SAMEA104317828	ERR2137528	Publication in process
148	**BPH3443**	2007	Australia	VIC	WGS Reads	Illumina NextSeq 500	SAMEA104317832	ERR2137532	Publication in process
149	**BPH3451**	2007	Australia	VIC	WGS Reads	Illumina NextSeq 500	SAMEA104317839	ERR2137539	Publication in process
150	**BPH3453**	2008	Australia	VIC	WGS Reads	Illumina NextSeq 500	SAMEA104317841	ERR2137541	Publication in process
151	**BPH3455**	2008	Australia	VIC	WGS Reads	Illumina NextSeq 500	SAMEA104317843	ERR2137543	Publication in process
152	**BPH3457**	2008	Australia	VIC	WGS Reads	Illumina NextSeq 500	SAMEA104317845	ERR2137545	Publication in process
153	**BPH3463**	2007	Australia	VIC	WGS Reads	Illumina NextSeq 500	SAMEA104317851	ERR2137551	Publication in process
154	**BPH3465**	2007	Australia	VIC	WGS Reads	Illumina NextSeq 500	SAMEA104317853	ERR2137553	Publication in process
155	**BPH3469**	2007	Australia	VIC	WGS Reads	Illumina NextSeq 500	SAMEA104317857	ERR2137557	Publication in process
156	**BPH3483**	2008	Australia	VIC	WGS Reads	Illumina NextSeq 500	SAMEA104317871	ERR2137571	Publication in process
157	**BPH3485**	2008	Australia	VIC	WGS Reads	Illumina NextSeq 500	SAMEA104317873	ERR2137573	Publication in process
158	**BPH3493**	2007	Australia	VIC	WGS Reads	Illumina NextSeq 500	SAMEA104317881	ERR2137581	Publication in process
159	**BPH3495**	2008	Australia	VIC	WGS Reads	Illumina NextSeq 500	SAMEA104317883	ERR2137583	Publication in process
160	**BPH3499**	2007	Australia	VIC	WGS Reads	Illumina NextSeq 500	SAMEA104317886	ERR2137586	Publication in process
161	**BPH3507**	2007	Australia	VIC	WGS Reads	Illumina NextSeq 500	SAMEA104317894	ERR2137594	Publication in process
162	**BPH3511**	2007	Australia	VIC	WGS Reads	Illumina NextSeq 500	SAMEA104317898	ERR2137598	Publication in process
163	**BPH3523**	2007	Australia	VIC	WGS Reads	Illumina NextSeq 500	SAMEA104317910	ERR2137610	Publication in process
164	**BPH3525**	2007	Australia	VIC	WGS Reads	Illumina NextSeq 500	SAMEA104317912	ERR2137612	Publication in process
165	**BPH3527**	2007	Australia	VIC	WGS Reads	Illumina NextSeq 500	SAMEA104317914	ERR2137614	Publication in process
166	**BPH3529**	2007	Australia	VIC	WGS Reads	Illumina NextSeq 500	SAMEA104317916	ERR2137616	Publication in process
167	**BPH3531**	2007	Australia	VIC	WGS Reads	Illumina NextSeq 500	SAMEA104317918	ERR2137618	Publication in process
168	**BPH3533**	2008	Australia	VIC	WGS Reads	Illumina NextSeq 500	SAMEA104317920	ERR2137620	Publication in process
169	**BPH3535**	2007	Australia	VIC	WGS Reads	Illumina NextSeq 500	SAMEA104317922	ERR2137622	Publication in process
170	**BPH3541**	2008	Australia	VIC	WGS Reads	Illumina NextSeq 500	SAMEA104317928	ERR2137628	Publication in process
171	**BPH3543**	2007	Australia	VIC	WGS Reads	Illumina NextSeq 500	SAMEA104317930	ERR2137630	Publication in process
172	**BPH3545**	2008	Australia	VIC	WGS Reads	Illumina NextSeq 500	SAMEA104317932	ERR2137632	Publication in process
173	**BPH3547**	2007	Australia	VIC	WGS Reads	Illumina NextSeq 500	SAMEA104317934	ERR2137634	Publication in process
174	**BPH3549**	2007	Australia	VIC	WGS Reads	Illumina NextSeq 500	SAMEA104317936	ERR2137636	Publication in process
175	**BPH3632**	2008	Australia	NSW	WGS Reads	Illumina NextSeq 500	SAMEA104325186	ERR2145145	Publication in process
176	**BPH3634**	2007	Australia	NSW	WGS Reads	Illumina NextSeq 500	SAMEA104325188	ERR2145147	Publication in process
177	**BPH3636**	2007	Australia	NSW	WGS Reads	Illumina NextSeq 500	SAMEA104325190	ERR2145149	Publication in process
178	**BPH3638**	2007	Australia	NSW	WGS Reads	Illumina NextSeq 500	SAMEA104325192	ERR2145151	Publication in process
179	**BPH3640**	2007	Australia	NSW	WGS Reads	Illumina NextSeq 500	SAMEA104325194	ERR2145153	Publication in process
180	**BPH3643**	2007	Australia	NSW	WGS Reads	Illumina NextSeq 500	SAMEA104325197	ERR2145156	Publication in process
181	**BPH3644**	2007	Australia	NSW	WGS Reads	Illumina NextSeq 500	SAMEA104325198	ERR2145157	Publication in process
182	**BPH3646**	2008	Australia	NSW	WGS Reads	Illumina NextSeq 500	SAMEA104325200	ERR2145159	Publication in process
183	**BPH3648**	2008	Australia	NSW	WGS Reads	Illumina NextSeq 500	SAMEA104325202	ERR2145161	Publication in process
184	**BPH3650**	2008	Australia	NSW	WGS Reads	Illumina NextSeq 500	SAMEA104325204	ERR2145163	Publication in process
185	**BPH3652**	2007	Australia	NSW	WGS Reads	Illumina NextSeq 500	SAMEA104325206	ERR2145165	Publication in process
186	**BPH3658**	2007	Australia	NSW	WGS Reads	Illumina NextSeq 500	SAMEA104325212	ERR2145171	Publication in process
187	**BPH3660**	2007	Australia	NSW	WGS Reads	Illumina NextSeq 500	SAMEA104325214	ERR2145173	Publication in process

188	**BPH3662**	2007	Australia	NSW	WGS Reads	Illumina NextSeq 500	SAMEA104325216	ERR2145175	Publication in process
189	**BPH3664**	2008	Australia	NSW	WGS Reads	Illumina NextSeq 500	SAMEA104325218	ERR2145177	Publication in process
190	**BPH3666**	2007	Australia	NSW	WGS Reads	Illumina NextSeq 500	SAMEA104325220	ERR2145179	Publication in process
191	**BPH3668**	2007	Australia	NSW	WGS Reads	Illumina NextSeq 500	SAMEA104325222	ERR2145181	Publication in process
192	**BPH3670**	2008	Australia	NSW	WGS Reads	Illumina NextSeq 500	SAMEA104325224	ERR2145183	Publication in process
193	**BPH3672**	2007	Australia	NSW	WGS Reads	Illumina NextSeq 500	SAMEA104325226	ERR2145185	Publication in process
194	**BPH3674**	2008	Australia	NSW	WGS Reads	Illumina NextSeq 500	SAMEA104325228	ERR2145187	Publication in process
195	**BPH3676**	2007	Australia	NSW	WGS Reads	Illumina NextSeq 500	SAMEA104325230	ERR2145189	Publication in process
196	**BPH3678**	2008	Australia	NSW	WGS Reads	Illumina NextSeq 500	SAMEA104325232	ERR2145191	Publication in process
197	**BPH3680**	2007	Australia	NSW	WGS Reads	Illumina NextSeq 500	SAMEA104325234	ERR2145193	Publication in process
198	**BPH3682**	2007	Australia	NSW	WGS Reads	Illumina NextSeq 500	SAMEA104325236	ERR2145195	Publication in process
199	**BPH3684**	2007	Australia	NSW	WGS Reads	Illumina NextSeq 500	SAMEA104325238	ERR2145197	Publication in process
200	**BPH3686**	2008	Australia	NSW	WGS Reads	Illumina NextSeq 500	SAMEA104325240	ERR2145199	Publication in process
201	**BPH3688**	2008	Australia	NSW	WGS Reads	Illumina NextSeq 500	SAMEA104325242	ERR2145201	Publication in process
202	**BPH3692**	2007	Australia	NSW	WGS Reads	Illumina NextSeq 500	SAMEA104325246	ERR2145205	Publication in process
203	**BPH3694**	2007	Australia	NSW	WGS Reads	Illumina NextSeq 500	SAMEA104325248	ERR2145207	Publication in process
204	**BPH3696**	2008	Australia	NSW	WGS Reads	Illumina NextSeq 500	SAMEA104325250	ERR2145209	Publication in process
205	**BPH3698**	2008	Australia	NSW	WGS Reads	Illumina NextSeq 500	SAMEA104325252	ERR2145211	Publication in process
206	**BPH3700**	2007	Australia	NSW	WGS Reads	Illumina NextSeq 500	SAMEA104325254	ERR2145213	Publication in process
207	**BPH3702**	2007	Australia	NSW	WGS Reads	Illumina NextSeq 500	SAMEA104325256	ERR2145215	Publication in process
208	**BPH3704**	2008	Australia	NSW	WGS Reads	Illumina NextSeq 500	SAMEA104325258	ERR2145217	Publication in process
209	**BPH3706**	2008	Australia	NSW	WGS Reads	Illumina NextSeq 500	SAMEA104325260	ERR2145219	Publication in process
210	**BPH3708**	2007	Australia	NSW	WGS Reads	Illumina NextSeq 500	SAMEA104325262	ERR2145221	Publication in process
211	**BPH3710**	2008	Australia	NSW	WGS Reads	Illumina NextSeq 500	SAMEA104325264	ERR2145223	Publication in process
212	**BPH3712**	2007	Australia	NSW	WGS Reads	Illumina NextSeq 500	SAMEA104325266	ERR2145225	Publication in process
**Additional WGS Data**									
213	**JKD6001**	2003	Australia	VIC	WGS Reads	Illumina Genome Analyzer IIx	SAMEA3212898	ERR732890	Baines. 2015. mBio. PMID: 25736880
214	**JKD6005**	2002	Australia	QLD	WGS Reads	Illumina Genome Analyzer IIx	SAMEA3212895	ERR732892	Baines. 2015. mBio. PMID: 25736880
215	**JKD6007**	2002	Australia	QLD	WGS Reads	Illumina MiSeq	SAMEA3212912	ERR732893	Baines. 2015. mBio. PMID: 25736880
216	**JKD6022**	2001	Australia	VIC	WGS Reads	Illumina MiSeq	SAMEA3212914	ERR732896	Baines. 2015. mBio. PMID: 25736880
217	**JKD6023**	2001	Australia	VIC	WGS Reads	Illumina Genome Analyzer IIx	SAMEA3212964	ERR732897	Baines. 2015. mBio. PMID: 25736880
218	**JKD6051**	2003	Australia	QLD	WGS Reads	Illumina Genome Analyzer IIx	SAMEA3212960	ERR732898	Baines. 2015. mBio. PMID: 25736880
219	**BPH2108**	1990	Australia	VIC	WGS Reads	Illumina NextSeq 500	SAMEA5042670	ERR2855738	This Study
220	**BPH2109**	1989	Australia	VIC	WGS Reads	Illumina NextSeq 500	SAMEA5042671	ERR2855739	This Study
221	**BPH2113**	1990	Australia	VIC	WGS Reads	Illumina NextSeq 500	SAMEA5042672	ERR2855740	This Study
222	**BPH2119**	1991	Australia	VIC	WGS Reads	Illumina NextSeq 500	SAMEA5042673	ERR2855741	This Study
223	**BPH2120**	1990	Australia	VIC	WGS Reads	Illumina NextSeq 500	SAMEA5042674	ERR2855742	This Study
224	**BPH2139**	1992	Australia	VIC	WGS Reads	Illumina NextSeq 500	SAMEA5042675	ERR2855743	This Study
225	**BPH2157**	1992	Australia	VIC	WGS Reads	Illumina NextSeq 500	SAMEA5042677	ERR2855745	This Study
226	**BPH2167**	1990	Australia	VIC	WGS Reads	Illumina NextSeq 500	SAMEA5042681	ERR2855749	This Study
227	**BPH2168**	1990	Australia	VIC	WGS Reads	Illumina NextSeq 500	SAMEA5042682	ERR2855750	This Study
228	**BPH2196**	1989	Australia	VIC	WGS Reads	Illumina NextSeq 500	SAMEA5042683	ERR2855751	This Study
229	**BPH2198**	1989	Australia	VIC	WGS Reads	Illumina NextSeq 500	SAMEA5042684	ERR2855752	This Study
230	**BPH2199**	1989	Australia	VIC	WGS Reads	Illumina NextSeq 500	SAMEA5042685	ERR2855753	This Study
231	**BPH2202**	1990	Australia	VIC	WGS Reads	Illumina NextSeq 500	SAMEA5042686	ERR2855754	This Study
232	**BPH2204**	1990	Australia	VIC	WGS Reads	Illumina NextSeq 500	SAMEA5042688	ERR2855756	This Study
233	**BPH2207**	1990	Australia	VIC	WGS Reads	Illumina NextSeq 500	SAMEA5042690	ERR2855758	This Study
234	**BPH2210**	1990	Australia	VIC	WGS Reads	Illumina NextSeq 500	SAMEA5042691	ERR2855759	This Study
235	**BPH2213**	1991	Australia	VIC	WGS Reads	Illumina NextSeq 500	SAMEA5042692	ERR2855760	This Study
236	**BPH2219**	1991	Australia	TAS	WGS Reads	Illumina NextSeq 500	SAMEA5042693	ERR2855761	This Study
237	**BPH2222**	1991	Australia	VIC	WGS Reads	Illumina NextSeq 500	SAMEA5042694	ERR2855762	This Study

238	**BPH2230**	1991	Australia	VIC	WGS Reads	Illumina NextSeq 500	SAMEA5042697	ERR2855765	This Study
239	**BPH2232**	1991	Australia	VIC	WGS Reads	Illumina NextSeq 500	SAMEA5042698	ERR2855766	This Study
240	**BPH2240**	1989	Australia	VIC	WGS Reads	Illumina NextSeq 500	SAMEA5042704	ERR2855772	This Study
241	**BPH2242**	1989	Australia	VIC	WGS Reads	Illumina NextSeq 500	SAMEA5042705	ERR2855773	This Study
242	**BPH2243**	1989	Australia	VIC	WGS Reads	Illumina NextSeq 500	SAMEA5042706	ERR2855774	This Study
243	**S20209-2015**	2015	Australia	NSW	WGS Reads	Illumina MiSeq	-	-	agargroup.org.au/agar-surveys
244	**S20305-2015**	2015	Australia	NSW	WGS Reads	Illumina MiSeq	-	-	agargroup.org.au/agar-surveys
245	**S20603-2015**	2015	Australia	NSW	WGS Reads	Illumina MiSeq	-	-	agargroup.org.au/agar-surveys
246	**S20607-2015**	2015	Australia	NSW	WGS Reads	Illumina MiSeq	-	-	agargroup.org.au/agar-surveys
247	**S20608-2015**	2015	Australia	NSW	WGS Reads	Illumina MiSeq	-	-	agargroup.org.au/agar-surveys
248	**S20610-2015**	2015	Australia	NSW	WGS Reads	Illumina MiSeq	-	-	agargroup.org.au/agar-surveys
249	**S20613-2015**	2015	Australia	NSW	WGS Reads	Illumina MiSeq	-	-	agargroup.org.au/agar-surveys
250	**S20617-2015**	2015	Australia	NSW	WGS Reads	Illumina MiSeq	-	-	agargroup.org.au/agar-surveys
251	**S20622-2015**	2015	Australia	NSW	WGS Reads	Illumina MiSeq	-	-	agargroup.org.au/agar-surveys
252	**S20623-2015**	2015	Australia	NSW	WGS Reads	Illumina MiSeq	-	-	agargroup.org.au/agar-surveys
253	**S20629-2015**	2015	Australia	NSW	WGS Reads	Illumina MiSeq	-	-	agargroup.org.au/agar-surveys
254	**S20826-2015**	2015	Australia	NSW	WGS Reads	Illumina MiSeq	-	-	agargroup.org.au/agar-surveys
255	**S23616-2015**	2015	Australia	NSW	WGS Reads	Illumina MiSeq	-	-	agargroup.org.au/agar-surveys
256	**S23619-2015**	2015	Australia	NSW	WGS Reads	Illumina MiSeq	-	-	agargroup.org.au/agar-surveys
257	**S31902-2015**	2015	Australia	VIC	WGS Reads	Illumina MiSeq	-	-	agargroup.org.au/agar-surveys
258	**S31904-2015**	2015	Australia	VIC	WGS Reads	Illumina MiSeq	-	-	agargroup.org.au/agar-surveys
259	**S31913-2015**	2015	Australia	VIC	WGS Reads	Illumina MiSeq	-	-	agargroup.org.au/agar-surveys
260	**S31918-2015**	2015	Australia	VIC	WGS Reads	Illumina MiSeq	-	-	agargroup.org.au/agar-surveys
261	**S32313-2015**	2015	Australia	VIC	WGS Reads	Illumina MiSeq	-	-	agargroup.org.au/agar-surveys
262	**S41210-2015**	2015	Australia	QLD	WGS Reads	Illumina MiSeq	-	-	agargroup.org.au/agar-surveys
263	**S51001-2015**	2015	Australia	NT	WGS Reads	Illumina MiSeq	-	-	agargroup.org.au/agar-surveys
264	**S51003-2015**	2015	Australia	NT	WGS Reads	Illumina MiSeq	-	-	agargroup.org.au/agar-surveys
265	**S51006-2015**	2015	Australia	NT	WGS Reads	Illumina MiSeq	-	-	agargroup.org.au/agar-surveys
266	**S51007-2015**	2015	Australia	NT	WGS Reads	Illumina MiSeq	-	-	agargroup.org.au/agar-surveys
267	**S51008-2015**	2015	Australia	NT	WGS Reads	Illumina MiSeq	-	-	agargroup.org.au/agar-surveys
268	**S51014-2015**	2015	Australia	NT	WGS Reads	Illumina MiSeq	-	-	agargroup.org.au/agar-surveys
269	**S51024-2015**	2015	Australia	NT	WGS Reads	Illumina MiSeq	-	-	agargroup.org.au/agar-surveys
270	**S51408-2015**	2015	Australia	SA	WGS Reads	Illumina MiSeq	-	-	agargroup.org.au/agar-surveys
271	**S51504-2015**	2015	Australia	SA	WGS Reads	Illumina MiSeq	-	-	agargroup.org.au/agar-surveys
272	**TW20**	2003	United Kingdom	-	Complete Genomes	-	FN433596, FN433597, FN43359[illegible]	-	Holden. 2010. JBact. PMID: 19948800
273	**Z172**	2010	Taiwan	-	Complete Genomes	Illumina/Solexa + PacBio	CP006838, CP006839, CP00684[illegible]	-	Chen. 2013. GenomeA. PMCID:PMC3853063
274	**T0131**	2006	China	-	Complete Genomes	Roche 454 / Solexa	CP002643	-	Li. 2011. JBact. PMID:21551295
275	**BMB9393**	1993	Brazil	-	Complete Genome	454 GS FLX Titanium	CP005288, CP005289	-	Costa. 2013. GenomeA. PMID:23929475
276	**XN108**	-	China	-	Complete Genomes	Ion Torrent PGM	CP007447	-	Zhang. 2014. GenomeA. PMID:25059856
277	**GV69**	1996	Brazil	-	Complete Genomes	454 GS FLX Titanium	CP009681; SAMN03144721	SRR2601051	Botelho. 2016. StandGenSci. PMID:27152133
278	**HC1335**	2001	Brazil	-	Complete Genomes	454 GS FLX Titanium	CP012012	-	Botelho. 2016. GenBiolEvol. PMID:27635055
279	**M92**	-	Canada	-	Complete Genomes	PacBio RS-II	CP015447	-	McClure. 2017. GenomeA. PMID:28596402
280	**2A8**	2001	Czech Republic	-	WGS Reads	Illumina HiSeq 2000	SAMEA1029549	ERR064912	Castillo-Ramirez. 2012. GB. PMID:23270620
281	**3HK**	2000	Czech Republic	-	WGS Reads	Illumina HiSeq 2000	SAMEA1029566	ERR064907	Castillo-Ramirez. 2012. GB. PMID:23270620
282	**AGT1**	1997	Argentina	-	WGS Reads	Illumina HiSeq 2000	SAMEA1029527	ERR064926	Castillo-Ramirez. 2012. GB. PMID:23270620
283	**AGT9**	1997	Argentina	-	WGS Reads	Illumina HiSeq 2000	SAMEA1029509	ERR064923	Castillo-Ramirez. 2012. GB. PMID:23270620
284	**AGT67**	1997	Argentina	-	WGS Reads	Illumina HiSeq 2000	SAMEA1029555	ERR064924	Castillo-Ramirez. 2012. GB. PMID:23270620
285	**AGT120**	1998	Argentina	-	WGS Reads	Illumina HiSeq 2000	SAMEA1029554	ERR064919	Castillo-Ramirez. 2012. GB. PMID:23270620
286	**ANS46**	1982	Australia	-	WGS Reads	Illumina HiSeq 2000	SAMEA1029537	ERR064898	Castillo-Ramirez. 2012. GB. PMID:23270620
287	**BK2421**	1996	United States	-	WGS Reads	Illumina HiSeq 2000	SAMEA1029510	ERR064899	Castillo-Ramirez. 2012. GB. PMID:23270620
288	**BRA2**	1997	Brazil	-	WGS Reads	Illumina HiSeq 2000	SAMEA1029514	ERR064914	Castillo-Ramirez. 2012. GB. PMID:23270620

289	**BRA36**	1997	Brazil	-	WGS Reads	Illumina HiSeq 2000	SAMEA1029559	ERR064917	Castillo-Ramirez. 2012. GB. PMID:23270620
290	**BZ48**	1997	Brazil	-	WGS Reads	Illumina HiSeq 2000	SAMEA1029508	ERR064918	Castillo-Ramirez. 2012. GB. PMID:23270620
291	**CHI59**	1998	China	-	WGS Reads	Illumina HiSeq 2000	SAMEA1029524	ERR064937	Castillo-Ramirez. 2012. GB. PMID:23270620
292	**CHI61**	1998	China	-	WGS Reads	Illumina HiSeq 2000	SAMEA1029538	ERR064938	Castillo-Ramirez. 2012. GB. PMID:23270620
293	**CHL1**	1997	Chile	-	WGS Reads	Illumina HiSeq 2000	SAMEA1029526	ERR064915	Castillo-Ramirez. 2012. GB. PMID:23270620
294	**CHL151**	1998	Chile	-	WGS Reads	Illumina HiSeq 2000	SAMEA1029539	ERR064916	Castillo-Ramirez. 2012. GB. PMID:23270620
295	**D71**	1996	Germany	-	WGS Reads	Illumina Genome Analyzer II	SAMEA862562	ERR024980	Castillo-Ramirez. 2012. GB. PMID:23270620
296	**D90**	1996	Germany	-	WGS Reads	Illumina Genome Analyzer II	SAMEA862585	ERR025801	Castillo-Ramirez. 2012. GB. PMID:23270620
297	**DEN907**	2001	Denmark	-	WGS Reads	Illumina HiSeq 2000	SAMEA1029548	ERR064948	Castillo-Ramirez. 2012. GB. PMID:23270620
298	**DEU2**	2009	Turkey	-	WGS Reads	Illumina Genome Analyzer II	SAMEA862577	ERR026652	Castillo-Ramirez. 2012. GB. PMID:23270620
299	**DEU3**	2009	Turkey	-	WGS Reads	Illumina Genome Analyzer II	SAMEA862574	ERR026653	Castillo-Ramirez. 2012. GB. PMID:23270620
300	**DEU5**	2009	Turkey	-	WGS Reads	Illumina Genome Analyzer II	SAMEA862580	ERR026655	Castillo-Ramirez. 2012. GB. PMID:23270620
301	**DEU6**	2009	Turkey	-	WGS Reads	Illumina Genome Analyzer II	SAMEA862581	ERR026656	Castillo-Ramirez. 2012. GB. PMID:23270620
302	**DEU8**	2009	Turkey	-	WGS Reads	Illumina Genome Analyzer II	SAMEA862579	ERR026658	Castillo-Ramirez. 2012. GB. PMID:23270620
303	**DEU10**	2009	Turkey	-	WGS Reads	Illumina Genome Analyzer II	SAMEA862583	ERR026649	Castillo-Ramirez. 2012. GB. PMID:23270620
304	**DEU11**	2009	Turkey	-	WGS Reads	Illumina Genome Analyzer II	SAMEA862554	ERR026650	Castillo-Ramirez. 2012. GB. PMID:23270620
305	**DEU12**	2009	Turkey	-	WGS Reads	Illumina Genome Analyzer II	SAMEA862553	ERR026651	Castillo-Ramirez. 2012. GB. PMID:23270620
306	**DEU14**	2008	Turkey	-	WGS Reads	Illumina Genome Analyzer II	SAMEA862555	ERR026664	Castillo-Ramirez. 2012. GB. PMID:23270620
307	**DEU15**	2008	Turkey	-	WGS Reads	Illumina Genome Analyzer II	SAMEA862558	ERR026665	Castillo-Ramirez. 2012. GB. PMID:23270620
308	**DEU16**	2008	Turkey	-	WGS Reads	Illumina Genome Analyzer II	SAMEA862557	ERR026666	Castillo-Ramirez. 2012. GB. PMID:23270620
309	**DEU17**	2008	Turkey	-	WGS Reads	Illumina Genome Analyzer II	SAMEA862559	ERR026667	Castillo-Ramirez. 2012. GB. PMID:23270620
310	**DEU19**	2008	Turkey	-	WGS Reads	Illumina Genome Analyzer II	SAMEA862561	ERR026668	Castillo-Ramirez. 2012. GB. PMID:23270620
311	**DEU20**	2008	Turkey	-	WGS Reads	Illumina Genome Analyzer II	SAMEA862560	ERR026669	Castillo-Ramirez. 2012. GB. PMID:23270620
312	**DEU23**	2008	Turkey	-	WGS Reads	Illumina Genome Analyzer II	SAMEA862541	ERR026662	Castillo-Ramirez. 2012. GB. PMID:23270620
313	**DEU25**	2008	Turkey	-	WGS Reads	Illumina Genome Analyzer II	SAMEA862542	ERR026663	Castillo-Ramirez. 2012. GB. PMID:23270620
314	**DEU27**	2007	Turkey	-	WGS Reads	Illumina Genome Analyzer II	SAMEA862536	ERR026677	Castillo-Ramirez. 2012. GB. PMID:23270620
315	**DEU28**	2007	Turkey	-	WGS Reads	Illumina Genome Analyzer II	SAMEA862537	ERR026678	Castillo-Ramirez. 2012. GB. PMID:23270620
316	**DEU29**	2007	Turkey	-	WGS Reads	Illumina Genome Analyzer II	SAMEA862538	ERR026679	Castillo-Ramirez. 2012. GB. PMID:23270620
317	**DEU30**	2007	Turkey	-	WGS Reads	Illumina Genome Analyzer II	SAMEA862531	ERR026680	Castillo-Ramirez. 2012. GB. PMID:23270620
318	**DEU35**	2007	Turkey	-	WGS Reads	Illumina Genome Analyzer II	SAMEA862662	ERR026683	Castillo-Ramirez. 2012. GB. PMID:23270620
319	**DEU36**	2007	Turkey	-	WGS Reads	Illumina Genome Analyzer II	SAMEA862661	ERR026684	Castillo-Ramirez. 2012. GB. PMID:23270620
320	**DEU37**	2007	Turkey	-	WGS Reads	Illumina Genome Analyzer II	SAMEA862660	ERR026674	Castillo-Ramirez. 2012. GB. PMID:23270620
321	**DEU38**	2006	Turkey	-	WGS Reads	Illumina Genome Analyzer II	SAMEA862667	ERR026675	Castillo-Ramirez. 2012. GB. PMID:23270620
322	**DEU39**	2006	Turkey	-	WGS Reads	Illumina Genome Analyzer II	SAMEA862666	ERR026676	Castillo-Ramirez. 2012. GB. PMID:23270620
323	**DEU41**	2006	Turkey	-	WGS Reads	Illumina Genome Analyzer II	SAMEA862664	ERR026690	Castillo-Ramirez. 2012. GB. PMID:23270620
324	**DEU42**	2006	Turkey	-	WGS Reads	Illumina Genome Analyzer II	SAMEA862655	ERR026691	Castillo-Ramirez. 2012. GB. PMID:23270620
325	**DEU43**	2006	Turkey	-	WGS Reads	Illumina Genome Analyzer II	SAMEA862654	ERR026692	Castillo-Ramirez. 2012. GB. PMID:23270620
326	**DEU46**	2006	Turkey	-	WGS Reads	Illumina Genome Analyzer II	SAMEA862570	ERR026694	Castillo-Ramirez. 2012. GB. PMID:23270620
327	**DEU47**	2006	Turkey	-	WGS Reads	Illumina Genome Analyzer II	SAMEA862573	ERR026695	Castillo-Ramirez. 2012. GB. PMID:23270620
328	**DEU49**	2006	Turkey	-	WGS Reads	Illumina Genome Analyzer II	SAMEA862567	ERR026697	Castillo-Ramirez. 2012. GB. PMID:23270620
329	**DEU50**	2006	Turkey	-	WGS Reads	Illumina Genome Analyzer II	SAMEA862566	ERR026687	Castillo-Ramirez. 2012. GB. PMID:23270620
330	**ES26**	1996	Spain	-	WGS Reads	Illumina Genome Analyzer II	SAMEA862530	ERR024981	Castillo-Ramirez. 2012. GB. PMID:23270620
331	**FFP103**	1990	Portugal	-	WGS Reads	Illumina HiSeq 2000	SAMEA1029533	ERR064932	Castillo-Ramirez. 2012. GB. PMID:23270620
332	**GRE4**	1998	Greece	-	WGS Reads	Illumina HiSeq 2000	SAMEA1029513	ERR064904	Castillo-Ramirez. 2012. GB. PMID:23270620
333	**GRE18**	1998	Greece	-	WGS Reads	Illumina HiSeq 2000	SAMEA1029546	ERR064902	Castillo-Ramirez. 2012. GB. PMID:23270620
334	**GRE108**	1998	Greece	-	WGS Reads	Illumina HiSeq 2000	SAMEA1029521	ERR064936	Castillo-Ramirez. 2012. GB. PMID:23270620
335	**GRE317**	1999	Greece	-	WGS Reads	Illumina HiSeq 2000	SAMEA1029564	ERR064903	Castillo-Ramirez. 2012. GB. PMID:23270620
336	**H202**	2006	Thailand	-	WGS Reads	Illumina Genome Analyzer II	SAMEA862584	ERR025802	Castillo-Ramirez. 2012. GB. PMID:23270620
337	**H211**	2006	Denmark	-	WGS Reads	Illumina Genome Analyzer II	SAMEA862528	ERR024972	Castillo-Ramirez. 2012. GB. PMID:23270620
338	**H216**	2006	Denmark	-	WGS Reads	Illumina Genome Analyzer II	SAMEA862587	ERR025803	Castillo-Ramirez. 2012. GB. PMID:23270620
339	**H24**	2005	Egypt	-	WGS Reads	Illumina Genome Analyzer II	SAMEA862527	ERR024973	Castillo-Ramirez. 2012. GB. PMID:23270620

340	**H482**	2007	Romania	-	WGS Reads	Illumina Genome Analyzer II	SAMEA862595	ERR024983	Castillo-Ramirez. 2012. GB. PMID:23270620
341	**HDG2**	1992	Portugal	-	WGS Reads	Illumina HiSeq 2000	SAMEA1029561	ERR064930	Castillo-Ramirez. 2012. GB. PMID:23270620
342	**HGSA9**	1997	Portugal	-	WGS Reads	Illumina HiSeq 2000	SAMEA1029534	ERR064921	Castillo-Ramirez. 2012. GB. PMID:23270620
343	**HGSA142**	2003	Portugal	-	WGS Reads	Illumina HiSeq 2000	SAMEA1029517	ERR064922	Castillo-Ramirez. 2012. GB. PMID:23270620
344	**HSA10**	1992	Portugal	-	WGS Reads	Illumina HiSeq 2000	SAMEA1029520	ERR064931	Castillo-Ramirez. 2012. GB. PMID:23270620
345	**HSA11**	1992	Portugal	-	WGS Reads	Illumina HiSeq 2000	SAMEA1029542	ERR064929	Castillo-Ramirez. 2012. GB. PMID:23270620
346	**HSJ216**	1997	Portugal	-	WGS Reads	Illumina HiSeq 2000	SAMEA1029550	ERR064920	Castillo-Ramirez. 2012. GB. PMID:23270620
347	**HU106**	1996	Hungary	-	WGS Reads	Illumina HiSeq 2000	SAMEA1029518	ERR064927	Castillo-Ramirez. 2012. GB. PMID:23270620
348	**HU109**	1996	Hungary	-	WGS Reads	Illumina HiSeq 2000	SAMEA1029567	ERR064909	Castillo-Ramirez. 2012. GB. PMID:23270620
349	**HU4**	2006	Turkey	-	WGS Reads	Illumina Genome Analyzer II	SAMEA862618	ERR026705	Castillo-Ramirez. 2012. GB. PMID:23270620
350	**HU5**	2006	Turkey	-	WGS Reads	Illumina Genome Analyzer II	SAMEA862625	ERR026706	Castillo-Ramirez. 2012. GB. PMID:23270620
351	**HU6**	2006	Turkey	-	WGS Reads	Illumina Genome Analyzer II	SAMEA862624	ERR026707	Castillo-Ramirez. 2012. GB. PMID:23270620
352	**HU7**	2006	Turkey	-	WGS Reads	Illumina Genome Analyzer II	SAMEA862623	ERR026708	Castillo-Ramirez. 2012. GB. PMID:23270620
353	**HU8**	2006	Turkey	-	WGS Reads	Illumina Genome Analyzer II	SAMEA862622	ERR026709	Castillo-Ramirez. 2012. GB. PMID:23270620
354	**HU9**	2007	Turkey	-	WGS Reads	Illumina Genome Analyzer II	SAMEA862627	ERR026710	Castillo-Ramirez. 2012. GB. PMID:23270620
355	**HU11**	2007	Turkey	-	WGS Reads	Illumina Genome Analyzer II	SAMEA862634	ERR026701	Castillo-Ramirez. 2012. GB. PMID:23270620
356	**HU13**	2007	Turkey	-	WGS Reads	Illumina Genome Analyzer II	SAMEA862636	ERR024668	Castillo-Ramirez. 2012. GB. PMID:23270620
357	**HU14**	2007	Turkey	-	WGS Reads	Illumina Genome Analyzer II	SAMEA862637	ERR024672	Castillo-Ramirez. 2012. GB. PMID:23270620
358	**HU15**	2007	Turkey	-	WGS Reads	Illumina Genome Analyzer II	SAMEA862630	ERR024673	Castillo-Ramirez. 2012. GB. PMID:23270620
359	**HU16**	2007	Turkey	-	WGS Reads	Illumina Genome Analyzer II	SAMEA862631	ERR024674	Castillo-Ramirez. 2012. GB. PMID:23270620
360	**HU17**	2007	Turkey	-	WGS Reads	Illumina Genome Analyzer II	SAMEA862632	ERR024675	Castillo-Ramirez. 2012. GB. PMID:23270620
361	**HU21**	2008	Turkey	-	WGS Reads	Illumina Genome Analyzer II	SAMEA862639	ERR024678	Castillo-Ramirez. 2012. GB. PMID:23270620
362	**HU23**	2009	Turkey	-	WGS Reads	Illumina Genome Analyzer II	SAMEA862643	ERR024679	Castillo-Ramirez. 2012. GB. PMID:23270620
363	**HU25**	1993	Brazil	-	WGS Reads	Illumina HiSeq 2000	SAMEA1029569	ERR024670	Castillo-Ramirez. 2012. GB. PMID:23270620
364	**HU26**	2009	Turkey	-	WGS Reads	Illumina Genome Analyzer II	SAMEA862644	ERR024671	Castillo-Ramirez. 2012. GB. PMID:23270620
365	**HUR18**	1997	Hungary	-	WGS Reads	Illumina HiSeq 2000	SAMEA1029551	ERR064910	Castillo-Ramirez. 2012. GB. PMID:23270620
366	**HUSA304**	1993	Hungary	-	WGS Reads	Illumina HiSeq 2000	SAMEA1029531	ERR064928	Castillo-Ramirez. 2012. GB. PMID:23270620
367	**ICP5011**	1993	Portugal	-	WGS Reads	Illumina HiSeq 2000	SAMEA1029545	ERR064933	Castillo-Ramirez. 2012. GB. PMID:23270620
368	**ICP5014**	1993	Portugal	-	WGS Reads	Illumina HiSeq 2000	SAMEA1029543	ERR064934	Castillo-Ramirez. 2012. GB. PMID:23270620
369	**ICP5062**	1993	Portugal	-	WGS Reads	Illumina HiSeq 2000	SAMEA1029562	ERR064935	Castillo-Ramirez. 2012. GB. PMID:23270620
370	**IU1**	2006	Turkey	-	WGS Reads	Illumina Genome Analyzer II	SAMEA862647	ERR024681	Castillo-Ramirez. 2012. GB. PMID:23270620
371	**IU2**	2006	Turkey	-	WGS Reads	Illumina Genome Analyzer II	SAMEA862646	ERR024685	Castillo-Ramirez. 2012. GB. PMID:23270620
372	**IU4**	2006	Turkey	-	WGS Reads	Illumina Genome Analyzer II	SAMEA862648	ERR024687	Castillo-Ramirez. 2012. GB. PMID:23270620
373	**IU6**	2006	Turkey	-	WGS Reads	Illumina Genome Analyzer II	SAMEA862651	ERR024688	Castillo-Ramirez. 2012. GB. PMID:23270620
374	**IU7**	2007	Turkey	-	WGS Reads	Illumina Genome Analyzer II	SAMEA862650	ERR024689	Castillo-Ramirez. 2012. GB. PMID:23270620
375	**IU9**	2007	Turkey	-	WGS Reads	Illumina Genome Analyzer II	SAMEA862617	ERR024691	Castillo-Ramirez. 2012. GB. PMID:23270620
376	**IU10**	2007	Turkey	-	WGS Reads	Illumina Genome Analyzer II	SAMEA862652	ERR024692	Castillo-Ramirez. 2012. GB. PMID:23270620
377	**IU11**	2007	Turkey	-	WGS Reads	Illumina Genome Analyzer II	SAMEA862653	ERR024682	Castillo-Ramirez. 2012. GB. PMID:23270620
378	**IU12**	2007	Turkey	-	WGS Reads	Illumina Genome Analyzer II	SAMEA862658	ERR024683	Castillo-Ramirez. 2012. GB. PMID:23270620
379	**IU13**	2007	Turkey	-	WGS Reads	Illumina Genome Analyzer II	SAMEA862659	ERR024684	Castillo-Ramirez. 2012. GB. PMID:23270620
380	**IU15**	2007	Turkey	-	WGS Reads	Illumina Genome Analyzer II	SAMEA862657	ERR024974	Castillo-Ramirez. 2012. GB. PMID:23270620
381	**LHH1**	1994	United States	-	WGS Reads	Illumina HiSeq 2000	SAMEA1029523	ERR064900	Castillo-Ramirez. 2012. GB. PMID:23270620
382	**LIT68**	1996	Lithuania	-	WGS Reads	Illumina Genome Analyzer II	SAMEA862586	ERR025804	Castillo-Ramirez. 2012. GB. PMID:23270620
383	**LIT76**	1996	Lithuania	-	WGS Reads	Illumina Genome Analyzer II	SAMEA862593	ERR025805	Castillo-Ramirez. 2012. GB. PMID:23270620
384	**LIT89**	1996	Lithuania	-	WGS Reads	Illumina Genome Analyzer II	SAMEA862592	ERR025796	Castillo-Ramirez. 2012. GB. PMID:23270620
385	**M116**	2004	Vietnam	-	WGS Reads	Illumina Genome Analyzer II	SAMEA862545	ERR024988	Castillo-Ramirez. 2012. GB. PMID:23270620
386	**M278**	2005	Portugal	-	WGS Reads	Illumina Genome Analyzer II	SAMEA862548	ERR024991	Castillo-Ramirez. 2012. GB. PMID:23270620
387	**M418**	2006	India	-	WGS Reads	Illumina Genome Analyzer II	SAMEA862549	ERR024992	Castillo-Ramirez. 2012. GB. PMID:23270620
388	**M592**	2006	Syria	-	WGS Reads	Illumina Genome Analyzer II	SAMEA862550	ERR024993	Castillo-Ramirez. 2012. GB. PMID:23270620
389	**M705**	2007	Thailand	-	WGS Reads	Illumina Genome Analyzer II	SAMEA862613	ERR024958	Castillo-Ramirez. 2012. GB. PMID:23270620
390	**M996**	2008	China	-	WGS Reads	Illumina Genome Analyzer II	SAMEA862551	ERR024994	Castillo-Ramirez. 2012. GB. PMID:23270620

391	**M1229**	2009	Denmark	-	WGS Reads	Illumina Genome Analyzer II	SAMEA862546	ERR024989	Castillo-Ramirez. 2012. GB. PMID:23270620
392	**MAL1**	1996	Malaysia	-	WGS Reads	Illumina Genome Analyzer II	SAMEA862552	ERR024984	Castillo-Ramirez. 2012. GB. PMID:23270620
393	**MAL3**	1996	Malaysia	-	WGS Reads	Illumina Genome Analyzer II	SAMEA862609	ERR024964	Castillo-Ramirez. 2012. GB. PMID:23270620
394	**MAL9**	1996	Malaysia	-	WGS Reads	Illumina Genome Analyzer II	SAMEA862607	ERR024966	Castillo-Ramirez. 2012. GB. PMID:23270620
395	**MAL11**	1996	Malaysia	-	WGS Reads	Illumina Genome Analyzer II	SAMEA862543	ERR024985	Castillo-Ramirez. 2012. GB. PMID:23270620
396	**MAL119**	1996	Malaysia	-	WGS Reads	Illumina Genome Analyzer II	SAMEA862611	ERR024962	Castillo-Ramirez. 2012. GB. PMID:23270620
397	**MAL215**	1996	Malaysia	-	WGS Reads	Illumina Genome Analyzer II	SAMEA862608	ERR024963	Castillo-Ramirez. 2012. GB. PMID:23270620
398	**MU1**	2008	Turkey	-	WGS Reads	Illumina Genome Analyzer II	SAMEA862569	ERR026688	Castillo-Ramirez. 2012. GB. PMID:23270620
399	**MU3**	2008	Turkey	-	WGS Reads	Illumina Genome Analyzer II	SAMEA862565	ERR026003	Castillo-Ramirez. 2012. GB. PMID:23270620
400	**MU4**	2008	Turkey	-	WGS Reads	Illumina Genome Analyzer II	SAMEA862564	ERR026007	Castillo-Ramirez. 2012. GB. PMID:23270620
401	**MU5**	2008	Turkey	-	WGS Reads	Illumina Genome Analyzer II	SAMEA862604	ERR026008	Castillo-Ramirez. 2012. GB. PMID:23270620
402	**MU6**	2008	Turkey	-	WGS Reads	Illumina Genome Analyzer II	SAMEA862605	ERR026009	Castillo-Ramirez. 2012. GB. PMID:23270620
403	**MU7**	2007	Turkey	-	WGS Reads	Illumina Genome Analyzer II	SAMEA862602	ERR026010	Castillo-Ramirez. 2012. GB. PMID:23270620
404	**MU9**	2007	Turkey	-	WGS Reads	Illumina Genome Analyzer II	SAMEA862603	ERR026011	Castillo-Ramirez. 2012. GB. PMID:23270620
405	**MU10**	2006	Turkey	-	WGS Reads	Illumina Genome Analyzer II	SAMEA862600	ERR026012	Castillo-Ramirez. 2012. GB. PMID:23270620
406	**MU20**	2009	Turkey	-	WGS Reads	Illumina Genome Analyzer II	SAMEA862620	ERR026703	Castillo-Ramirez. 2012. GB. PMID:23270620
407	**Na21**	2006	Sri Lanka	-	WGS Reads	Illumina Genome Analyzer II	SAMEA862544	ERR024986	Castillo-Ramirez. 2012. GB. PMID:23270620
408	**NA32**	2006	Denmark	-	WGS Reads	Illumina Genome Analyzer II	SAMEA862615	ERR024967	Castillo-Ramirez. 2012. GB. PMID:23270620
409	**P32**	1996	Poland	-	WGS Reads	Illumina Genome Analyzer II	SAMEA862589	ERR025795	Castillo-Ramirez. 2012. GB. PMID:23270620
410	**R35**	1987	United States	-	WGS Reads	Illumina HiSeq 2000	SAMEA1029532	ERR064901	Castillo-Ramirez. 2012. GB. PMID:23270620
411	**RA3**	1996	Argentina	-	WGS Reads	Illumina Genome Analyzer II	SAMEA862588	ERR025798	Castillo-Ramirez. 2012. GB. PMID:23270620
412	**RA6**	1996	Argentina	-	WGS Reads	Illumina Genome Analyzer II	SAMEA862591	ERR025799	Castillo-Ramirez. 2012. GB. PMID:23270620
413	**RA7**	1996	Argentina	-	WGS Reads	Illumina Genome Analyzer II	SAMEA862616	ERR024968	Castillo-Ramirez. 2012. GB. PMID:23270620
414	**S2**	2006	Thailand	-	WGS Reads	Illumina HiSeq 2000	SAMEA1029563	ERR064953	Castillo-Ramirez. 2012. GB. PMID:23270620
415	**S7**	2006	Thailand	-	WGS Reads	Illumina HiSeq 2000	SAMEA1029556	ERR064946	Castillo-Ramirez. 2012. GB. PMID:23270620
416	**S21**	2006	Thailand	-	WGS Reads	Illumina HiSeq 2000	SAMEA1029536	ERR064958	Castillo-Ramirez. 2012. GB. PMID:23270620
417	**S24**	2006	Thailand	-	WGS Reads	Illumina HiSeq 2000	SAMEA1029553	ERR064957	Castillo-Ramirez. 2012. GB. PMID:23270620
418	**S25**	2006	Thailand	-	WGS Reads	Illumina HiSeq 2000	SAMEA1029535	ERR064951	Castillo-Ramirez. 2012. GB. PMID:23270620
419	**S26**	2006	Thailand	-	WGS Reads	Illumina HiSeq 2000	SAMEA1029511	ERR064949	Castillo-Ramirez. 2012. GB. PMID:23270620
420	**S38**	2006	Thailand	-	WGS Reads	Illumina HiSeq 2000	SAMEA1029568	ERR064947	Castillo-Ramirez. 2012. GB. PMID:23270620
421	**S39**	2006	Thailand	-	WGS Reads	Illumina HiSeq 2000	SAMEA1029558	ERR064959	Castillo-Ramirez. 2012. GB. PMID:23270620
422	**S40**	2006	Thailand	-	WGS Reads	Illumina HiSeq 2000	SAMEA1029528	ERR064944	Castillo-Ramirez. 2012. GB. PMID:23270620
423	**S42**	2006	Thailand	-	WGS Reads	Illumina HiSeq 2000	SAMEA1029515	ERR064955	Castillo-Ramirez. 2012. GB. PMID:23270620
424	**S71**	2007	Thailand	-	WGS Reads	Illumina HiSeq 2000	SAMEA1029541	ERR064943	Castillo-Ramirez. 2012. GB. PMID:23270620
425	**S78**	2007	Thailand	-	WGS Reads	Illumina HiSeq 2000	SAMEA1029525	ERR064954	Castillo-Ramirez. 2012. GB. PMID:23270620
426	**S81**	2007	Thailand	-	WGS Reads	Illumina HiSeq 2000	SAMEA1029565	ERR064956	Castillo-Ramirez. 2012. GB. PMID:23270620
427	**S85**	2007	Thailand	-	WGS Reads	Illumina HiSeq 2000	SAMEA1029529	ERR064939	Castillo-Ramirez. 2012. GB. PMID:23270620
428	**S87**	2007	Thailand	-	WGS Reads	Illumina HiSeq 2000	SAMEA1029512	ERR064941	Castillo-Ramirez. 2012. GB. PMID:23270620
429	**S93**	2007	Thailand	-	WGS Reads	Illumina HiSeq 2000	SAMEA1029560	ERR064942	Castillo-Ramirez. 2012. GB. PMID:23270620
430	**S97**	2007	Thailand	-	WGS Reads	Illumina HiSeq 2000	SAMEA1029522	ERR064950	Castillo-Ramirez. 2012. GB. PMID:23270620
431	**S102**	2007	Thailand	-	WGS Reads	Illumina HiSeq 2000	SAMEA1029519	ERR064945	Castillo-Ramirez. 2012. GB. PMID:23270620
432	**S106**	2007	Thailand	-	WGS Reads	Illumina HiSeq 2000	SAMEA1029547	ERR064952	Castillo-Ramirez. 2012. GB. PMID:23270620
433	**S130**	2007	Thailand	-	WGS Reads	Illumina HiSeq 2000	SAMEA1029516	ERR064940	Castillo-Ramirez. 2012. GB. PMID:23270620
434	**TUR1**	1996	Turkey	-	WGS Reads	Illumina HiSeq 2000	SAMEA1029552	ERR064906	Castillo-Ramirez. 2012. GB. PMID:23270620
435	**TUR9**	1995	Turkey	-	WGS Reads	Illumina HiSeq 2000	SAMEA1029530	ERR064905	Castillo-Ramirez. 2012. GB. PMID:23270620
436	**TUR27**	1996	Turkey	-	WGS Reads	Illumina HiSeq 2000	SAMEA1029557	ERR064908	Castillo-Ramirez. 2012. GB. PMID:23270620
437	**UK102**	1996	United Kingdom	-	WGS Reads	Illumina Genome Analyzer II	SAMEA862590	ERR025800	Castillo-Ramirez. 2012. GB. PMID:23270620
438	**URU34**	1997	Uruguay	-	WGS Reads	Illumina HiSeq 2000	SAMEA1029544	ERR064925	Castillo-Ramirez. 2012. GB. PMID:23270620
439	**URU110**	1998	Uruguay	-	WGS Reads	Illumina HiSeq 2000	SAMEA1029540	ERR064911	Castillo-Ramirez. 2012. GB. PMID:23270620
440	**SR2852**	2005	United States	-	WGS Reads	Illumina HiSeq 2000	SAMN02649763	SRR1177896	Alam. GenomeBiolEvol. 2014. PMID:24787619
441	**031_01**	2001	Singapore	-	WGS Reads	Illumina Genome Analyzer II	SAMEA800273	ERR029457	Hsu. 2015. GenBiol. PMID:25903077

442	**362_05**	2005	Singapore	-	WGS Reads	Illumina Genome Analyzer II	SAMEA800274	ERR029458	Hsu. 2015. GenBiol. PMID:25903077
443	**058_08**	2008	Singapore	-	WGS Reads	Illumina Genome Analyzer II	SAMEA800253	ERR029464	Hsu. 2015. GenBiol. PMID:25903077
444	**352_05**	2005	Singapore	-	WGS Reads	Illumina Genome Analyzer II	SAMEA800280	ERR029467	Hsu. 2015. GenBiol. PMID:25903077
445	**131_09**	2009	Singapore	-	WGS Reads	Illumina Genome Analyzer II	SAMEA800276	ERR029469	Hsu. 2015. GenBiol. PMID:25903077
446	**009_10**	2010	Singapore	-	WGS Reads	Illumina Genome Analyzer II	SAMEA800287	ERR029470	Hsu. 2015. GenBiol. PMID:25903077
447	**134_09**	2009	Singapore	-	WGS Reads	Illumina Genome Analyzer II	SAMEA800277	ERR029472	Hsu. 2015. GenBiol. PMID:25903077
448	**135_09**	2009	Singapore	-	WGS Reads	Illumina Genome Analyzer II	SAMEA800278	ERR029473	Hsu. 2015. GenBiol. PMID:25903077
449	**049_05**	2005	Singapore	-	WGS Reads	Illumina Genome Analyzer II	SAMEA800292	ERR029476	Hsu. 2015. GenBiol. PMID:25903077
450	**CGH1_08**	2008	Singapore	-	WGS Reads	Illumina Genome Analyzer II	SAMEA800297	ERR030155	Hsu. 2015. GenBiol. PMID:25903077
451	**CGH1_10**	2010	Singapore	-	WGS Reads	Illumina Genome Analyzer II	SAMEA800177	ERR030157	Hsu. 2015. GenBiol. PMID:25903077
452	**CGH2_08**	2008	Singapore	-	WGS Reads	Illumina Genome Analyzer II	SAMEA800181	ERR030159	Hsu. 2015. GenBiol. PMID:25903077
453	**CGH3_10**	2010	Singapore	-	WGS Reads	Illumina Genome Analyzer II	SAMEA800213	ERR030170	Hsu. 2015. GenBiol. PMID:25903077
454	**CGH6_06**	2006	Singapore	-	WGS Reads	Illumina Genome Analyzer II	SAMEA800382	ERR030173	Hsu. 2015. GenBiol. PMID:25903077
455	**CGH4_09**	2009	Singapore	-	WGS Reads	Illumina Genome Analyzer II	SAMEA800215	ERR030176	Hsu. 2015. GenBiol. PMID:25903077
456	**CGH4_10**	2010	Singapore	-	WGS Reads	Illumina Genome Analyzer II	SAMEA800216	ERR030177	Hsu. 2015. GenBiol. PMID:25903077
457	**CGH5_09**	2009	Singapore	-	WGS Reads	Illumina Genome Analyzer II	SAMEA800383	ERR030182	Hsu. 2015. GenBiol. PMID:25903077
458	**012_00**	2000	Singapore	-	WGS Reads	Illumina Genome Analyzer II	SAMEA800207	ERR030186	Hsu. 2015. GenBiol. PMID:25903077
459	**011_00**	2000	Singapore	-	WGS Reads	Illumina Genome Analyzer II	SAMEA800205	ERR030191	Hsu. 2015. GenBiol. PMID:25903077
460	**003_06**	2006	Singapore	-	WGS Reads	Illumina Genome Analyzer II	SAMEA800206	ERR030192	Hsu. 2015. GenBiol. PMID:25903077
461	**106_06**	2006	Singapore	-	WGS Reads	Illumina Genome Analyzer II	SAMEA800200	ERR030194	Hsu. 2015. GenBiol. PMID:25903077
462	**100_06**	2006	Singapore	-	WGS Reads	Illumina Genome Analyzer II	SAMEA800201	ERR030195	Hsu. 2015. GenBiol. PMID:25903077
463	**106_07**	2007	Singapore	-	WGS Reads	Illumina Genome Analyzer II	SAMEA800360	ERR030200	Hsu. 2015. GenBiol. PMID:25903077
464	**339_07**	2007	Singapore	-	WGS Reads	Illumina Genome Analyzer II	SAMEA800357	ERR030201	Hsu. 2015. GenBiol. PMID:25903077
465	**157_04**	2004	Singapore	-	WGS Reads	Illumina Genome Analyzer II	SAMEA800333	ERR030202	Hsu. 2015. GenBiol. PMID:25903077
466	**019_01**	2001	Singapore	-	WGS Reads	Illumina Genome Analyzer II	SAMEA800332	ERR030203	Hsu. 2015. GenBiol. PMID:25903077
467	**002_00**	2000	Singapore	-	WGS Reads	Illumina Genome Analyzer II	SAMEA800331	ERR030204	Hsu. 2015. GenBiol. PMID:25903077
468	**020_01**	2001	Singapore	-	WGS Reads	Illumina Genome Analyzer II	SAMEA800329	ERR030205	Hsu. 2015. GenBiol. PMID:25903077
469	**101_07**	2007	Singapore	-	WGS Reads	Illumina Genome Analyzer II	SAMEA800327	ERR030209	Hsu. 2015. GenBiol. PMID:25903077
470	**030_05**	2005	Singapore	-	WGS Reads	Illumina Genome Analyzer II	SAMEA800258	ERR030213	Hsu. 2015. GenBiol. PMID:25903077
471	**255_04**	2004	Singapore	-	WGS Reads	Illumina Genome Analyzer II	SAMEA800353	ERR030216	Hsu. 2015. GenBiol. PMID:25903077
472	**259_04**	2004	Singapore	-	WGS Reads	Illumina Genome Analyzer II	SAMEA800354	ERR030217	Hsu. 2015. GenBiol. PMID:25903077
473	**263_04**	2004	Singapore	-	WGS Reads	Illumina Genome Analyzer II	SAMEA800352	ERR030219	Hsu. 2015. GenBiol. PMID:25903077
474	**264_04**	2004	Singapore	-	WGS Reads	Illumina Genome Analyzer II	SAMEA800261	ERR030220	Hsu. 2015. GenBiol. PMID:25903077
475	**028_01**	2001	Singapore	-	WGS Reads	Illumina Genome Analyzer II	SAMEA800260	ERR030221	Hsu. 2015. GenBiol. PMID:25903077
476	**069_02**	2002	Singapore	-	WGS Reads	Illumina Genome Analyzer II	SAMEA800271	ERR030224	Hsu. 2015. GenBiol. PMID:25903077
477	**006_00**	2000	Singapore	-	WGS Reads	Illumina Genome Analyzer II	SAMEA800285	ERR030228	Hsu. 2015. GenBiol. PMID:25903077
478	**067_02**	2002	Singapore	-	WGS Reads	Illumina Genome Analyzer II	SAMEA800265	ERR030232	Hsu. 2015. GenBiol. PMID:25903077
479	**068_02**	2002	Singapore	-	WGS Reads	Illumina Genome Analyzer II	SAMEA800272	ERR030233	Hsu. 2015. GenBiol. PMID:25903077
480	**071_03**	2003	Singapore	-	WGS Reads	Illumina Genome Analyzer II	SAMEA800269	ERR030235	Hsu. 2015. GenBiol. PMID:25903077
481	**078_05**	2005	Singapore	-	WGS Reads	Illumina Genome Analyzer II	SAMEA800264	ERR030238	Hsu. 2015. GenBiol. PMID:25903077
482	**086_03**	2003	Singapore	-	WGS Reads	Illumina Genome Analyzer II	SAMEA800263	ERR030239	Hsu. 2015. GenBiol. PMID:25903077
483	**009_00**	2000	Singapore	-	WGS Reads	Illumina Genome Analyzer II	SAMEA800336	ERR030240	Hsu. 2015. GenBiol. PMID:25903077
484	**TTSH1_01**	2001	Singapore	-	WGS Reads	Illumina Genome Analyzer II	SAMEA800251	ERR030242	Hsu. 2015. GenBiol. PMID:25903077
485	**TTSH2_07**	2007	Singapore	-	WGS Reads	Illumina Genome Analyzer II	SAMEA800229	ERR030249	Hsu. 2015. GenBiol. PMID:25903077
486	**TTSH1_10**	2010	Singapore	-	WGS Reads	Illumina Genome Analyzer II	SAMEA800219	ERR030252	Hsu. 2015. GenBiol. PMID:25903077
487	**TTSH2_03**	2003	Singapore	-	WGS Reads	Illumina Genome Analyzer II	SAMEA800226	ERR030255	Hsu. 2015. GenBiol. PMID:25903077
488	**TTSH2_08**	2008	Singapore	-	WGS Reads	Illumina Genome Analyzer II	SAMEA800230	ERR030259	Hsu. 2015. GenBiol. PMID:25903077
489	**TTSH3_07**	2007	Singapore	-	WGS Reads	Illumina Genome Analyzer II	SAMEA800196	ERR030260	Hsu. 2015. GenBiol. PMID:25903077
490	**TTSH3_08**	2008	Singapore	-	WGS Reads	Illumina Genome Analyzer II	SAMEA800232	ERR030261	Hsu. 2015. GenBiol. PMID:25903077
491	**TTSH3_03**	2003	Singapore	-	WGS Reads	Illumina Genome Analyzer II	SAMEA800234	ERR030266	Hsu. 2015. GenBiol. PMID:25903077
492	**TTSH4_02**	2002	Singapore	-	WGS Reads	Illumina Genome Analyzer II	SAMEA800293	ERR030276	Hsu. 2015. GenBiol. PMID:25903077

493	**TTSH4_03**	2003	Singapore	-	WGS Reads	Illumina Genome Analyzer II	SAMEA800372	ERR030277	Hsu. 2015. GenBiol. PMID:25903077
494	**TTSH5_02**	2002	Singapore	-	WGS Reads	Illumina Genome Analyzer II	SAMEA800345	ERR031666	Hsu. 2015. GenBiol. PMID:25903077
495	**TTSH5_06**	2006	Singapore	-	WGS Reads	Illumina Genome Analyzer II	SAMEA800346	ERR031670	Hsu. 2015. GenBiol. PMID:25903077
496	**WAU_8**	1982	Singapore	-	WGS Reads	Illumina Genome Analyzer II	SAMEA1021823	ERR053014	Hsu. 2015. GenBiol. PMID:25903077
497	**WAU_9**	1985	Singapore	-	WGS Reads	Illumina Genome Analyzer II	SAMEA1021807	ERR053015	Hsu. 2015. GenBiol. PMID:25903077
498	**WAU_10**	1985	Singapore	-	WGS Reads	Illumina Genome Analyzer II	SAMEA1021856	ERR053016	Hsu. 2015. GenBiol. PMID:25903077
499	**WAU_16**	1996	Singapore	-	WGS Reads	Illumina Genome Analyzer II	SAMEA1022012	ERR053022	Hsu. 2015. GenBiol. PMID:25903077
500	**WAU_17**	1996	Singapore	-	WGS Reads	Illumina Genome Analyzer II	SAMEA1021973	ERR053023	Hsu. 2015. GenBiol. PMID:25903077
501	**WAU_18**	1996	Singapore	-	WGS Reads	Illumina Genome Analyzer II	SAMEA1021952	ERR053024	Hsu. 2015. GenBiol. PMID:25903077
502	**WAU_19**	1996	Singapore	-	WGS Reads	Illumina Genome Analyzer II	SAMEA1021934	ERR053025	Hsu. 2015. GenBiol. PMID:25903077
503	**WAU_21**	1996	Singapore	-	WGS Reads	Illumina Genome Analyzer II	SAMEA1021910	ERR053027	Hsu. 2015. GenBiol. PMID:25903077
504	**WAU_26**	1997	Singapore	-	WGS Reads	Illumina Genome Analyzer II	SAMEA1021849	ERR053032	Hsu. 2015. GenBiol. PMID:25903077
505	**WAU_29**	1997	Singapore	-	WGS Reads	Illumina Genome Analyzer II	SAMEA1021995	ERR053035	Hsu. 2015. GenBiol. PMID:25903077
506	**WAU_31**	1997	Singapore	-	WGS Reads	Illumina Genome Analyzer II	SAMEA1021970	ERR053037	Hsu. 2015. GenBiol. PMID:25903077
507	**WAU_34**	1997	Singapore	-	WGS Reads	Illumina Genome Analyzer II	SAMEA1021933	ERR053040	Hsu. 2015. GenBiol. PMID:25903077
508	**WAU_39**	1997	Singapore	-	WGS Reads	Illumina Genome Analyzer II	SAMEA1021951	ERR053044	Hsu. 2015. GenBiol. PMID:25903077
509	**T9_N3**	2008	Thailand	-	WGS Reads	Illumina Genome Analyzer II	SAMEA958822	ERR023888	Tong. GenRes. 2015. PMID:25491771
510	**T234_W2**	2008	Thailand	-	WGS Reads	Illumina Genome Analyzer II	SAMEA958806	ERR023872	Tong. GenRes. 2015. PMID:25491771
511	**T71_N2**	2008	Thailand	-	WGS Reads	Illumina Genome Analyzer II	SAMEA958988	ERR033836	Tong. GenRes. 2015. PMID:25491771
512	**T99_N5**	2008	Thailand	-	WGS Reads	Illumina Genome Analyzer II	SAMEA958963	ERR033828	Tong. GenRes. 2015. PMID:25491771
513	**T12_N1**	2008	Thailand	-	WGS Reads	Illumina Genome Analyzer II	SAMEA958848	ERR023884	Tong. GenRes. 2015. PMID:25491771
514	**T271_C1**	2008	Thailand	-	WGS Reads	Illumina Genome Analyzer II	SAMEA958810	ERR023867	Tong. GenRes. 2015. PMID:25491771
515	**T35_N2**	2008	Thailand	-	WGS Reads	Illumina Genome Analyzer II	SAMEA958775	ERR033823	Tong. GenRes. 2015. PMID:25491771
516	**T95_N2**	2008	Thailand	-	WGS Reads	Illumina Genome Analyzer II	SAMEA958962	ERR033833	Tong. GenRes. 2015. PMID:25491771
517	**T192_T3**	2008	Thailand	-	WGS Reads	Illumina Genome Analyzer II	SAMEA958900	ERR023907	Tong. GenRes. 2015. PMID:25491771
518	**T303_N3**	2008	Thailand	-	WGS Reads	Illumina Genome Analyzer II	SAMEA958836	ERR033080	Tong. GenRes. 2015. PMID:25491771
519	**SA13002**	2012	China	-	WGS Reads	Illumina MiSeq	SAMN03856026	SRR2097047	Kong. 2016. PLoS One. PMID:26950298
520	**SA13005**	2013	China	-	WGS Reads	Illumina MiSeq	SAMN03878399	SRR2106469	Kong. 2016. PLoS One. PMID:26950298
521	**SA13007**	2012	China	-	WGS Reads	Illumina MiSeq	SAMN03878400	SRR2106470	Kong. 2016. PLoS One. PMID:26950298
522	**SA13009**	2012	China	-	WGS Reads	Illumina MiSeq	SAMN03878401	SRR2106471	Kong. 2016. PLoS One. PMID:26950298
523	**SA13012**	2012	China	-	WGS Reads	Illumina MiSeq	SAMN03878402	SRR2106472	Kong. 2016. PLoS One. PMID:26950298
524	**SA13023**	2013	China	-	WGS Reads	Illumina MiSeq	SAMN03878403	SRR2106473	Kong. 2016. PLoS One. PMID:26950298
525	**BSAC_st3021**	2010	England	-	WGS Reads	Illumina HiSeq 2000	SAMEA1464569	ERR124493	Reuter. GenRes. 2016. PMID:26672018
526	**BSAC_st3046**	2010	England	-	WGS Reads	Illumina HiSeq 2000	SAMEA1464296	ERR124495	Reuter. GenRes. 2016. PMID:26672018
527	**BSAC_st697**	2003	England	-	WGS Reads	Illumina HiSeq 2000	SAMEA1464583	ERR129279	Reuter. GenRes. 2016. PMID:26672018
528	**D8**	2007	Gambia	-	WGS Reads	Illumina MiSeq	SAMEA3727072	ERR1213802	Senghore. AppEnvMicro. 2016. PMID:27474712
529	**DS_009**	2011	Thailand	-	WGS Reads	Illumina HiSeq 2000	SAMEA3448838	ERR1069915	Moradigaravand. mBio. 2017. PMID: 28679748
530	**DS_014**	2011	Thailand	-	WGS Reads	Illumina HiSeq 2000	SAMEA3448843	ERR1069920	Moradigaravand. mBio. 2017. PMID: 28679748
531	**US_002**	2011	Thailand	-	WGS Reads	Illumina HiSeq 2000	SAMEA3448943	ERR1070030	Moradigaravand. mBio. 2017. PMID: 28679748
532	**US_008**	2011	Thailand	-	WGS Reads	Illumina HiSeq 2000	SAMEA3448948	ERR1070035	Moradigaravand. mBio. 2017. PMID: 28679748
533	**US_030**	2011	Thailand	-	WGS Reads	Illumina HiSeq 2000	SAMEA3448965	ERR1070052	Moradigaravand. mBio. 2017. PMID: 28679748
534	**US_017**	2011	Thailand	-	WGS Reads	Illumina HiSeq 2000	SAMEA3448955	ERR1070042	Moradigaravand. mBio. 2017. PMID: 28679748

Figure S1

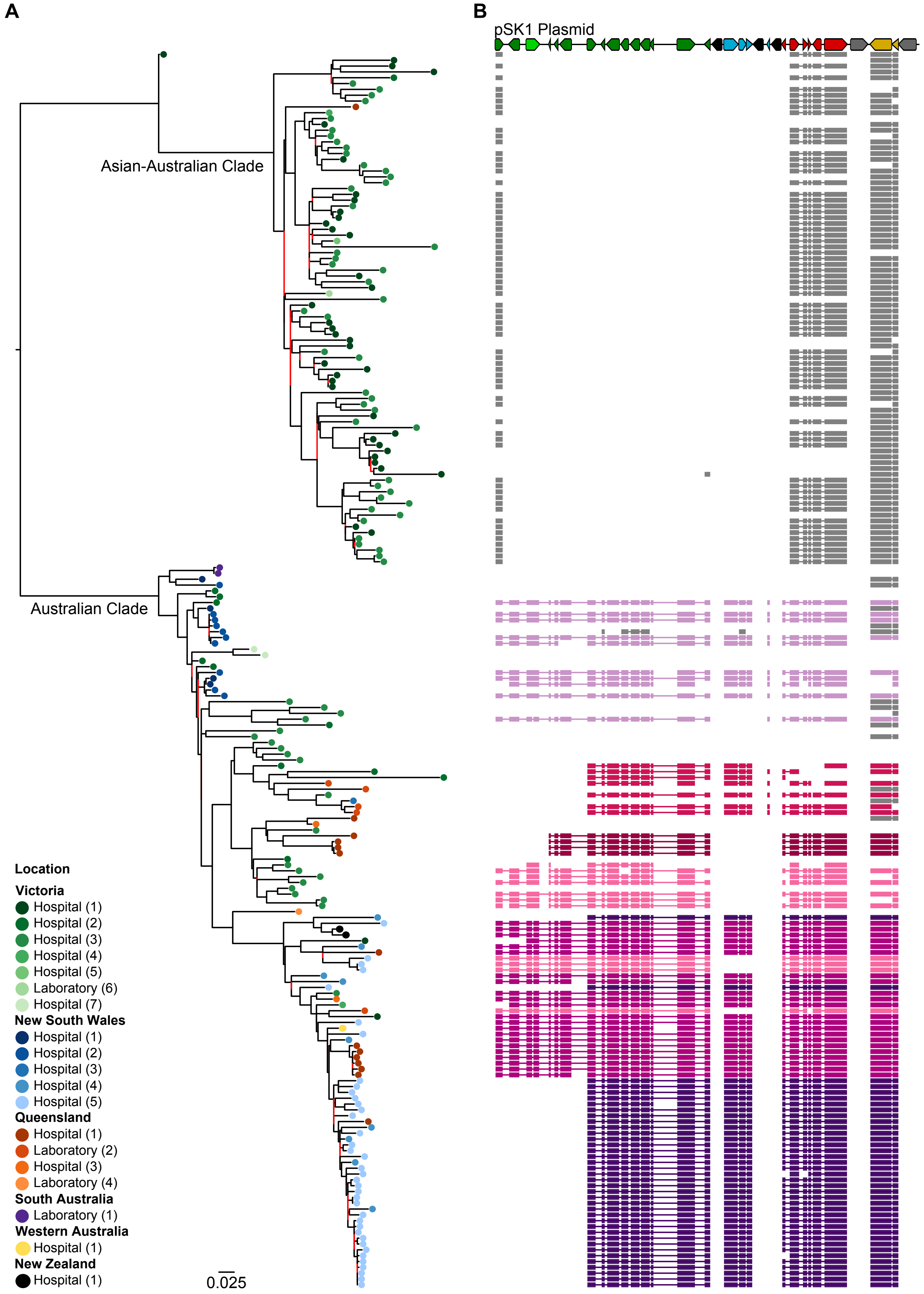

Supplementary Figure S1. pSK1 plasmid gene presence and synteny. (A) Maximum likelihood phylogenetic tree inferred from 3,883 core genome SNPs illustrates the population structure of ST239 *S. aureus* in Australia. Tips are coloured based on location (refer to key). Branches with < 70% bootstrap support are coloured red. (B) Coloured blocks represent the identification of a pSK1 gene orthologue, using a 95% amino acid homology threshold (excluding insertion sequences). Box length is reflective of gene length and ordered based on pSK1 (Figure 2A). Boxes are linked if orthologues were found to be syntenic. Coloured boxes reflect the six identified patterns.

Figure S2

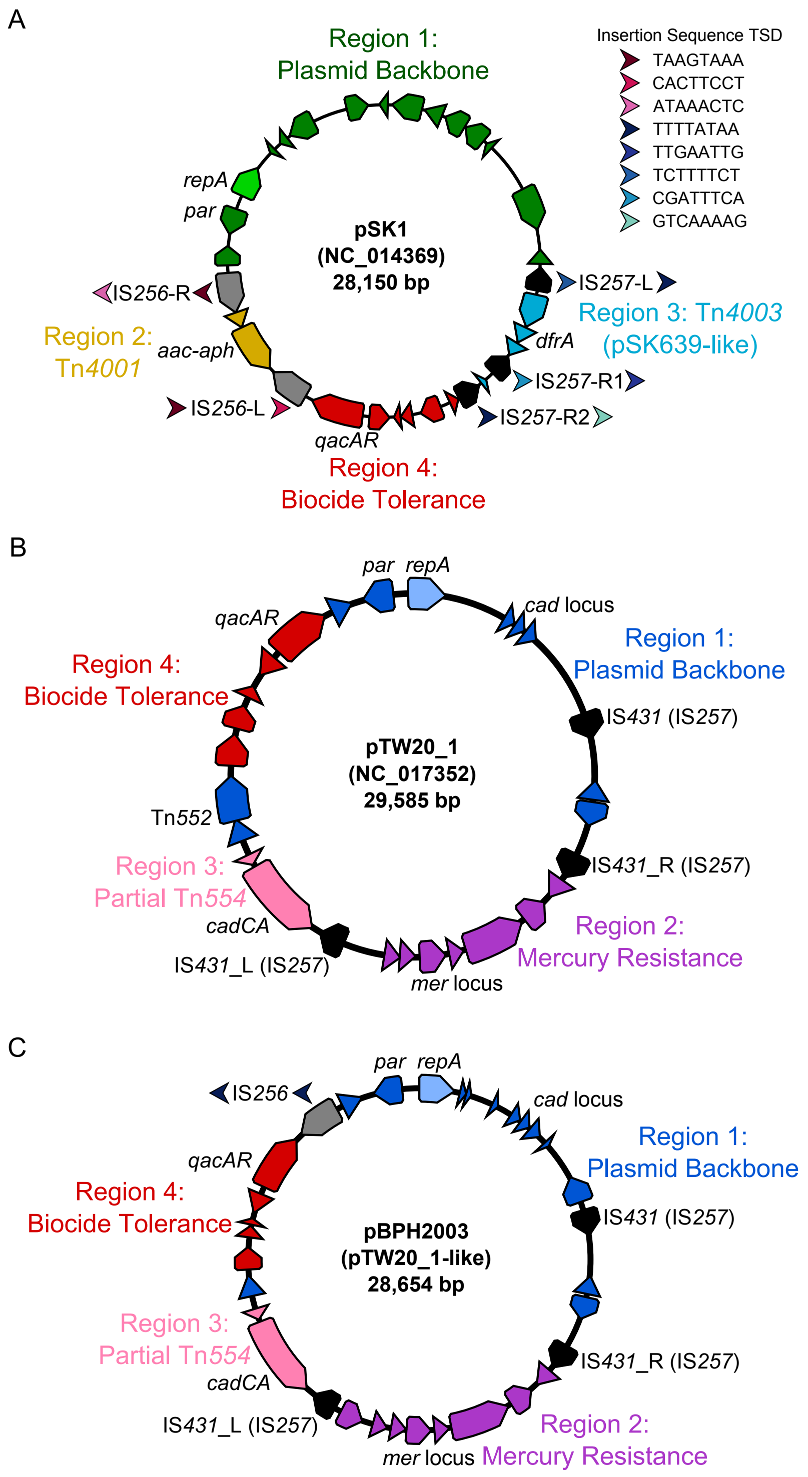

Supplementary Figure S2. Structure of pSK1, pTW20_1 and pBPH2003 plasmids. Plasmid genes have been coloured based on defined plasmid regions. Insertion sequences (IS) are coloured grey (IS256) and black (IS257), with target site duplications (TSD) illustrated: arrows indicate upstream/downstream sequences, orientation, and are coloured to represent unique sequences (refer to key). (A) pSK1; sequence and annotations are that previously published, accession NC_014369. (B) pTW20_1; sequence and annotations are that previously published, accession NC_017352. (C) pBPH2003; a pTW20_1-like plasmid representative of that harboured in isoaltes from the Asian-Australian ST239 clade.

Supplementary Figure 3

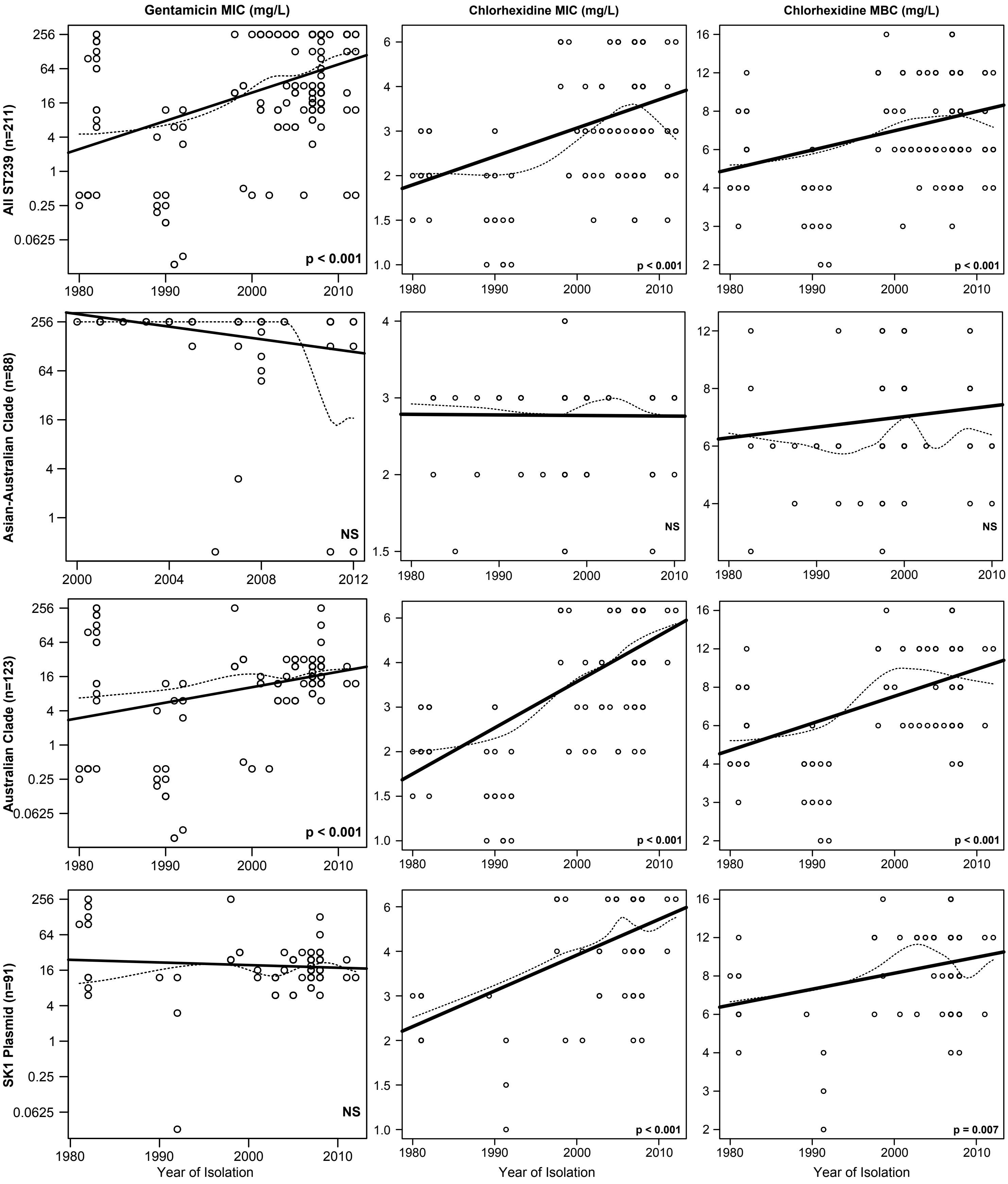

Supplementary Figure S3. Modelling temporal-association in phenotypic susceptibility data. Graphs depict linear models developed to explore the potential association between gentamicin MIC, and chlorhexidine MIC and MBC with the year in which isolates were recovered. The dotted lines indicate the smoothed mean MIC and the bold lines indicate the fitted linear model. Four populations were tested (from top to bottom): (i) All ST239 MRSA (n = 211), (ii) the Asian-Australian clade (n = 88), (iii) the Australian clade (n = 123), and (iv) the pSK1-like plasmid harbouring population (n = 91). Note: reference JKD6008 (Australian clade) was not tested.

Supplementary Figure 4

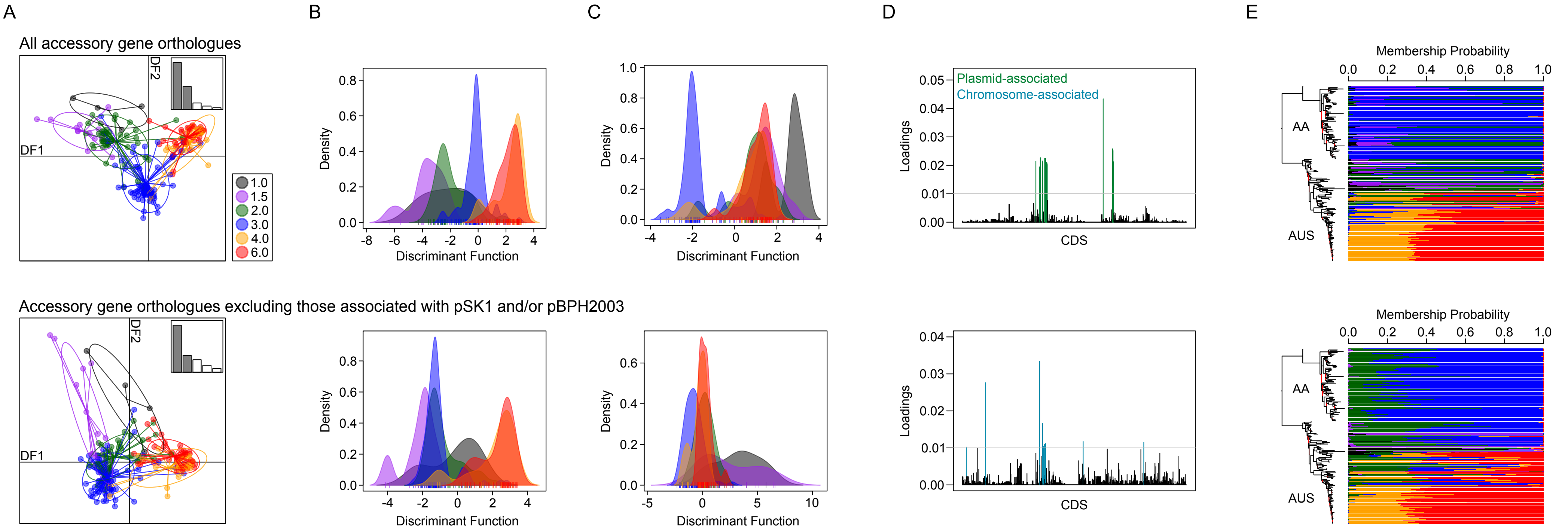

Supplementary Figure S4. Exploration of gene presence/absence signatures associated with phenotypic chlorhexidine tolerance. The graphs illustrate the findings from two Discriminant Analysis of Principle Components (DAPC) models, used to investigate genetic signatures in the ST239 MRSA population associated with chlorhexidine MIC. The first model (top panel) examined all accessory gene orthologues (clustered at 95% amino acid homology), and in the second model (bottom panel) gene orthologues associated with either pSK1 or pBPH2003 (representative of the pTW20_1-like plasmid recovered from the Asian-Australian clade) were excluded. The prior hypothesised sub-divisions represent the phenotypic CHX MIC values for the population, as indicated in the key. (A) Scatter plots illustrate the clustering of isolates across the two most discriminant functions. The density plots illustrate the same data in a one-dimensional format for the first (B) and second (C) most discriminant functions. (D) Loading plots illustrate the contributions of all gene orthologues to the DAPC models, with the most highly contributing variables (> 0.01) coloured based on whether they represent a plasmid-associated (green) or chromosomal gene orthologues (blue). (E) Membership graphs illustrate the membership profiles for all isolates, aligned to a maximum likelihood phylogenetic tree for the ST239 population. The probability index indicates the likelihood of assigning an isolate back to one of the hypothesised sub-divisions.

Figure S5

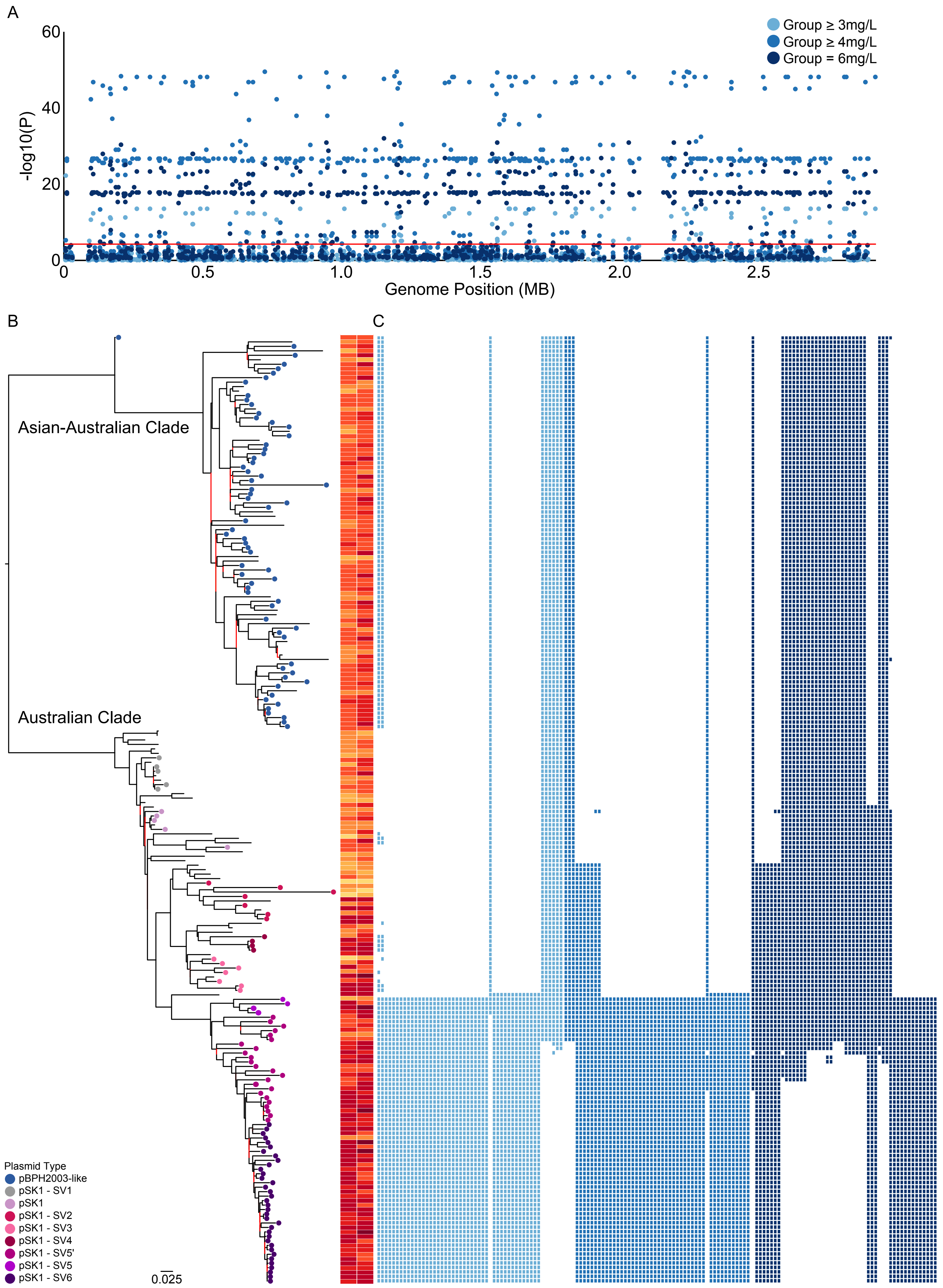

Supplementary Figure S5. Exploration of mutations associated with phenotypic chlorhexidine tolerance. (A) Manhattan plot illustrates the results of three Genome Wide Association Studies (GWAS) conducted to identify core genome SNPs associated with CHX MIC, tested at three thresholds: MIC > 3 mg/L, > 4 mg/L or = 6 mg/L. Red line indicates the threshold for significance (with Bonferroni correction). (B) Maximum likelihood phylogenetic tree is that from Figure S1, and adjacent are the phenotypic CHX MIC and MBC values (coloured yellow to red with increasing value). (C) The presence (blue) or absence (white) of the 50 most significantly associated mutations detected at each phenotypic threshold are illustrated.
